# Supplementary material for: Derivatives Incorporating Acridine, Pyrrole, and Thiazolidine Rings as Promising Antitumor Agents
Source: Molecules. 2023 Sep 14;28(18):6616. doi: 10.3390/molecules28186616 (PMC10537105; doi:10.3390/molecules28186616)
Supplement: Supplementary file 1 [file molecules-28-06616-s001.zip › molecules-2605934-supplementary.pdf]

Derivatives Incorporating Acridine, Pyrrole, and Thiazolidine Rings as Promising Antitumor Agents

Monika Garberová [1], Ivan Potočňák [1], Monika Tvrdoňová [1], Monika Majirská [2], Martina Bago-Pilátová [2], Slávka Bekešová [3], Andrej Kováč [4], Peter Takáč [4], Krutika Khiratkar [5], Zuzana Kudličková [1,*], Ján Elečko [1] and Mária Vilková [1,*]

1 Institute of Chemistry, Faculty of Science, Pavol Jozef Šafárik University, Moyzesova 11, 040 01 Košice, Slovakia; monika.garberova@student.upjs.sk (M.G.); ivan.potocnak@upjs.sk (I.P.); monika.tvrdonova@upjs.sk (M.T.); jan.elecko@upjs.sk (J.E.)
2 Department of Pharmacology, Faculty of Medicine, Pavol Jozef Šafárik University, Trieda SNP 1, 040 01 Košice, Slovakia; monika.majirska@student.upjs.sk (M.M.); martina.pilatova@upjs.sk (M.B.-P.)
3 Thermo Fisher Scientific, Mlynské Nivy 5, 821 09 Bratislava, Slovakia; slavka.bekesova@thermofisher.com
4 Department of Pharmacology and Toxicology, University of Veterinary Medicine and Pharmacy, Komenského 73, 041 81 Košice, Slovakia; rej.kovac@uvlf.sk (A.K.); peter.takac@uvlf.sk (P.T.)
5 Institute of Neuroimmunology, Slovak Academy of Sciences, Dúbravská Cesta 9, 845 10 Bratislava, Slovakia; krutika.khiratkar@savba.sk
* Correspondence: zuzana.kudlickova@upjs.sk (Z.K.); maria.vilkova@upjs.sk (M.V.)

Content

1 Figures and Tables related to the Results and Discussion section 3

2 NMR and IR spectra of synthesized derivatives 9

2.1 3-{4'-Cyano-5'-oxo-1',5'-dihydro-10*H*-spiro[acridine-9,2'-pyrrol]-1'-yl}-1-phenylthiourea (**6a**) 9

2.2 3-{4'-Cyano-5'-oxo-1',5'-dihydro-10H-spiro[acridine-9,2'-pyrrol]-1'-yl}-1-(4-methoxyphenyl)thiourea (**6b**) 12

2.3 3-{4'-Cyano-5'-oxo-1',5'-dihydro-10H-spiro[acridine-9,2'-pyrrol]-1'-yl}-1-(3-methoxyphenyl)thiourea (**6c**) 15

2.4 3-{4'-Cyano-5'-oxo-1',5'-dihydro-10*H*-spiro[acridine-9,2'-pyrrol]-1'-yl}-1-(4-fluorophenyl)thiourea (**6d**) 18

2.5 3-{4'-Cyano-5'-oxo-1',5'-dihydro-10*H*-spiro[acridine-9,2'-pyrrol]-1'-yl}-1-(4-nitrophenyl)thiourea (**6e**) 21

2.6 5'-Oxo-1'-{[(2*Z*)-4-oxo-3-phenyl-1,3-thiazolidin-2-ylidene]amino}-1',5'-dihydro-10*H*-spiro[acridine-9,2'-pyrrole]-4'-carbonitrile (**7a**) 24

2.7 1'-{[(2*Z*)-3-(4-Methoxyphenyl)-4-oxo-1,3-thiazolidin-2-ylidene]amino}-5'-oxo-1',5'-dihydro-10*H*-spiro[acridine-9,2'-pyrrole]-4'-carbonitrile (**7b**) 27

2.8 1'-{[(2*Z*)-3-(3-Methoxyphenyl)-4-oxo-1,3-thiazolidin-2-ylidene]amino}-5'-oxo-1',5'-dihydro-10*H*-spiro[acridine-9,2'-pyrrole]-4'-carbonitrile (**7c**) 30

2.9 1'-{[(2*Z*)-3-(4-Fluorophenyl)-4-oxo-1,3-thiazolidin-2-ylidene]amino}-5'-oxo-1',5'-dihydro-10*H*-spiro[acridine-9,2'-pyrrole]-4'-carbonitrile (**7d**) 33

2.10 1'-{[(2*Z*)-3-(4-Nitrophenyl)-4-oxo-1,3-thiazolidin-2-ylidene]amino}-5'-oxo-1',5'-dihydro-10*H*-spiro[acridine-9,2'-pyrrole]-4'-carbonitrile (**7e**) 36

2.11 Ethyl 2-[(2*Z*,5*Z*)-2-({4'-cyano-5'-oxo-1',5'-dihydro-10*H*-spiro[acridine-9,2'-pyrrol]-1'-yl}imino)-4-oxo-3-phenyl-1,3-thiazolidin-5-ylidene]acetate (**8a**) 39

2.12 Ethyl 2-[(2*Z*,5*Z*)-2-({4'-cyano-5'-oxo-1',5'-dihydro-10*H*-spiro[acridine-9,2'-pyrrol]-1'-yl}imino)-3-(4-methoxyphenyl)-4-oxo-1,3-thiazolidin-5-ylidene]acetate (**8b**) 42

2.13 Ethyl 2-[(2*Z*,5*Z*)-2-({4'-cyano-5'-oxo-1',5'-dihydro-10*H*-spiro[acridine-9,2'-pyrrol]-1'-yl}imino)-3-(3-methoxyphenyl)-4-oxo-1,3-thiazolidin-5-ylidene]acetate (**8c**) 45

2.14 Ethyl 2-[(2*Z*,5*Z*)-2-({4'-cyano-5'-oxo-1',5'-dihydro-10*H*-spiro[acridine-9,2'-pyrrol]-1'-yl}imino)-3-(4-fluorophenyl)-4-oxo-1,3-thiazolidin-5-ylidene]acetate (**8d**) 48

2.15 Ethyl 2-[(2*Z*,5*Z*)-2-({4'-cyano-5'-oxo-1',5'-dihydro-10*H*-spiro[acridine-9,2'-pyrrol]-1'-yl}imino)-3-(4-nitrophenyl)-4-oxo-1,3-thiazolidin-5-ylidene]acetate (**8e**) 51

2.16 1'-{[(2*Z*)-5-Methyl-4-oxo-3-phenyl-1,3-thiazolidin-2-ylidene]amino}-5'-oxo-1',5'-dihydro-10*H*-spiro[acridine-9,2'-pyrrole]-4'-carbonitrile (**9a**) 54

2.17 1'-{[(2*Z*)-3-(4-Methoxyphenyl)-5-methyl-4-oxo-1,3-thiazolidin-2-ylidene]amino}-5'-oxo-1',5'-dihydro-10*H*-spiro[acridine-9,2'-pyrrole]-4'-carbonitrile (**9b**) 57

2.18 1'-{[(2Z)-3-(3-Methoxyphenyl)-5-methyl-4-oxo-1,3-thiazolidin-2-ylidene]amino}-5'-oxo-1',5'-dihydro-10H-spiro[acridine-9,2'-pyrrole]-4'-carbonitrile (**9c**) 60

2.19 1'-{[(2*Z*)-3-(4-Fluorophenyl)-5-methyl-4-oxo-1,3-thiazolidin-2-ylidene]amino}-5'-oxo-1',5'-dihydro-10*H*-spiro[acridine-9,2'-pyrrole]-4'-carbonitrile (**9d**) 63

2.20 1'-{[(2*Z*)-5-Methyl-3-(4-nitrophenyl)-4-oxo-1,3-thiazolidin-2-ylidene]amino}-5'-oxo-1',5'-dihydro-10*H*-spiro[acridine-9,2'-pyrrole]-4'-carbonitrile (**9e**) 66

2.21 5'-Oxo-1'-{[(2*Z*)-4-oxo-3-phenyl-5-propyl-1,3-thiazolidin-2-ylidene]amino}-1',5'-dihydro-10*H*-spiro[acridine-9,2'-pyrrole]-4'-carbonitrile (**10a**) 69

2.22 1'-{[(2*Z*)-3-(4-Methoxyphenyl)-4-oxo-5-propyl-1,3-thiazolidin-2-ylidene]amino}-5'-oxo-1',5'-dihydro-10*H*-spiro[acridine-9,2'-pyrrole]-4'-carbonitrile (**10b**) 72

2.23 1'-{[(2Z)-3-(3-Methoxyphenyl)-4-oxo-5-propyl-1,3-thiazolidin-2-ylidene]amino}-5'-oxo-1',5'-dihydro-10H-spiro[acridine-9,2'-pyrrole]-4'-carbonitrile (**10c**) 75

2.24 1'-{[(2*Z*)-3-(4-Fluorophenyl)-4-oxo-5-propyl-1,3-thiazolidin-2-ylidene]amino}-5'-oxo-1',5'-dihydro-10*H*-spiro[acridine-9,2'-pyrrole]-4'-carbonitrile (**10d**) 78

2.25 1'-{[(2*Z*)-3-(4-Nitrophenyl)-4-oxo-5-propyl-1,3-thiazolidin-2-ylidene]amino}-5'-oxo-1',5'-dihydro-10*H*-spiro[acridine-9,2'-pyrrole]-4'-carbonitrile (**10e**) 81

1 Figures and Tables related to the Results and Discussion section

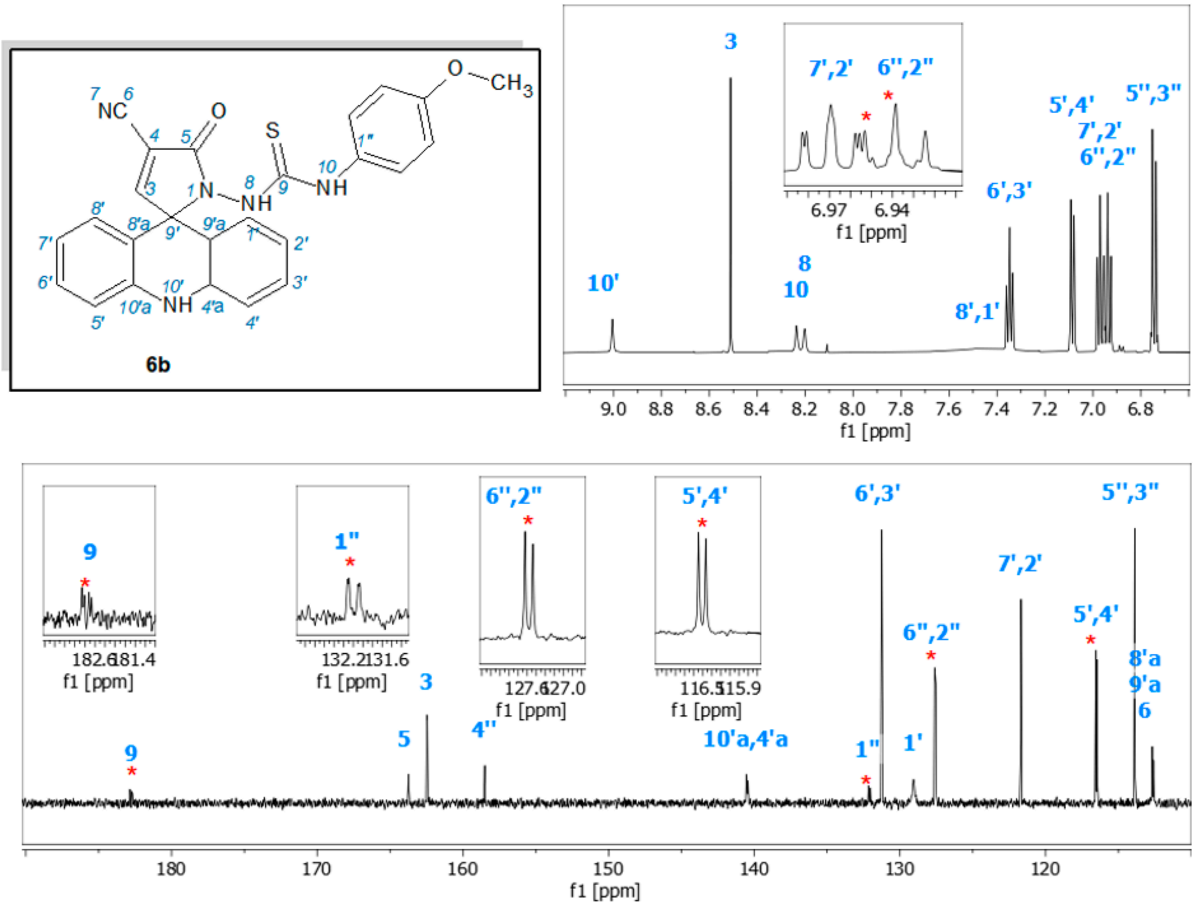

Figure S1. ^{1}H (600 MHz, acetone-d_6) and ^{13}C NMR (150 MHz, acetone-d_6) spectra of derivative **6b**. Red stars indicate doubled signals.

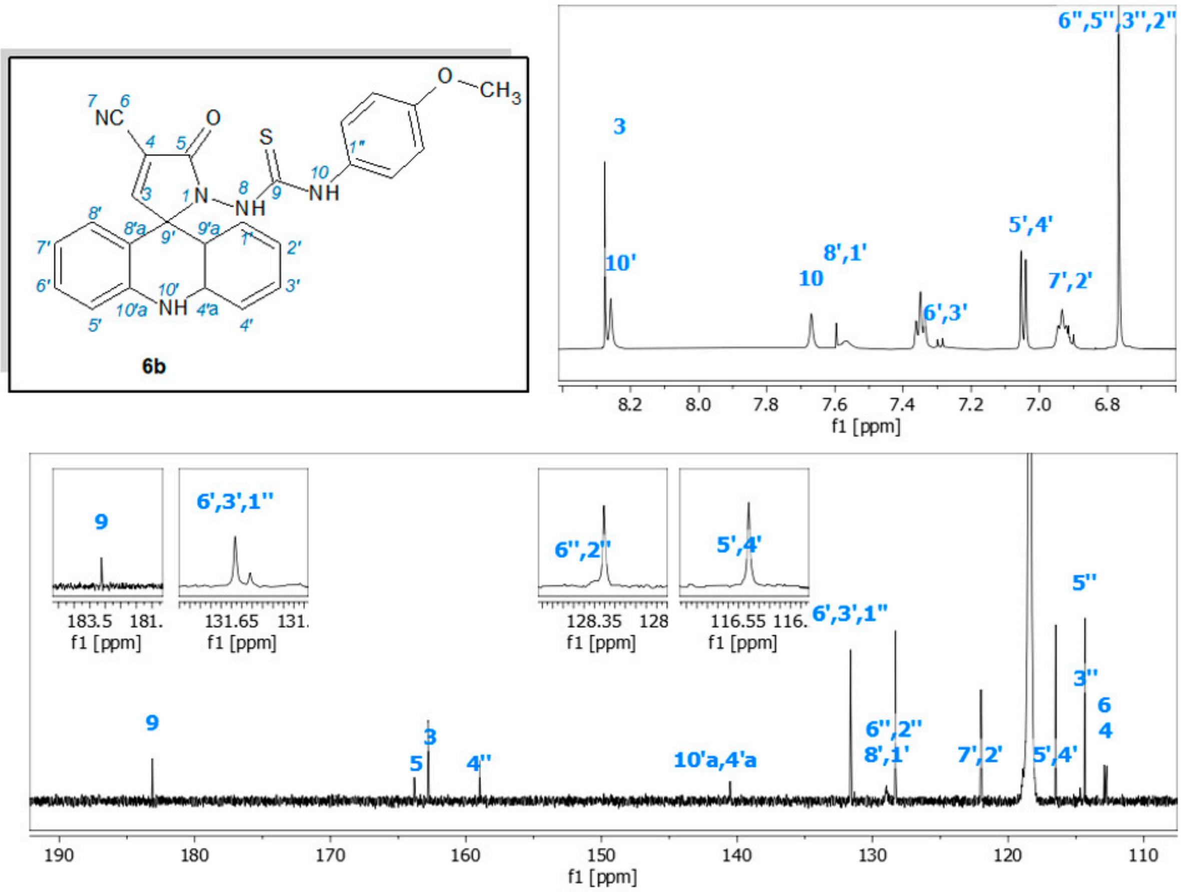

Figure S2. ^{1}H (600 MHz, acetonitrile-d_3) and ^{13}C NMR (150 MHz, acetonitrile-d_3) spectra of derivative **6b**.

Table S1. Reaction times and yields of reactions of spiro compounds **6a**–**e** with bifunctional reagents.

Product	R	R^1	Reagent	Yield [%]	Reaction time [h]
**7a**	Ph	H	MBA	79	2
**7b**	(4-OMe)Ph	H	MBA	66	2,5
**7c**	(3-OMe)Ph	H	MBA	83	2,5
**7d**	(4-F)Ph	H	MBA	70	2
**7e**	(4-NO_2)Ph	H	MBA	41	3
**8a**	Ph	=CH-COOEt	DEAD	75	7
**8b**	(4-OMe)Ph	=CH-COOEt	DEAD	57	7
**8c**	(3-OMe)Ph	=CH-COOEt	DEAD	66	7
**8d**	(4-F)Ph	=CH-COOEt	DEAD	60	7
**8e**	(4-NO_2)Ph	=CH-COOEt	DEAD	60	7
**9a**	Ph	Me	EBP	77	3,5
**9b**	(4-OMe)Ph	Me	EBP	64	3
**9c**	(3-OMe)Ph	Me	EBP	64	2,5
**9d**	(4-F)Ph	Me	EBP	73	2
**9e**	(4-NO_2)Ph	Me	EBP	38	4
**10a**	Ph	Pr	EBV	69	24
**10b**	(4-OMe)Ph	Pr	EBV	46	48
**10c**	(3-OMe)Ph	Pr	EBV	71	24
**10d**	(4-F)Ph	Pr	EBV	46	48

**10e**	(4-NO_2)Ph	Pr	EBV	60	48

Table S2. HR MS data for compounds **6a**–**e**, **7a**–**e**, **8a**–**e**, **9a**–**e** and **10a**–**e**.

		Name	Formula	Exact mass [g.mol^{-1}]	Measured mass [g.mol^{-1}]	m/z unit	Mass error [ppm]
**6a**	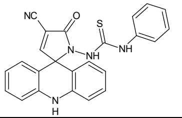	*3-{4'-cyano-5'-oxo-1',5'-dihydro-10H-spiro[acridine-9,2'-pyrrol]-1'-yl}-1-phenylthiourea*	$C_{24}H_{17}N_5OS$	424.12266	424.12300	3.40×10^{-4}	0.80165
**6b**	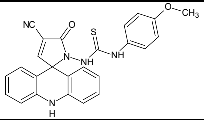	*3-{4'-cyano-5'-oxo-1',5'-dihydro-10H-spiro[acridine-9,2'-pyrrol]-1'-yl}-1-(4-methoxyphenyl)thiourea*	$C_{25}H_{19}N_5O_2S$	454.13322	454.13420	9.80×10^{-4}	2.15796
**6c**	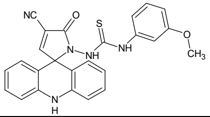	*3-{4'-cyano-5'-oxo-1',5'-dihydro-10H-spiro[acridine-9,2'-pyrrol]-1'-yl}-1-(3-methoxyphenyl)thiourea*	$C_{25}H_{19}N_5O_2S$	454.13322	454.13390	6.80×10^{-4}	1.49736
**6d**	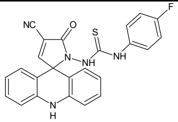	*3-{4'-cyano-5'-oxo-1',5'-dihydro-10H-spiro[acridine-9,2'-pyrrol]-1'-yl}-1-(4-fluorophenyl)thiourea*	$C_{24}H_{16}FN_5OS$	442.11324	442.11340	1.60×10^{-4}	0.36190
**6e**	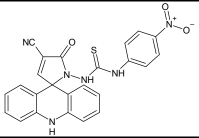	*3-{4'-cyano-5'-oxo-1',5'-dihydro-10H-spiro[acridine-9,2'-pyrrol]-1'-yl}-1-(4-nitrophenyl)thiourea*	$C_{24}H_{16}N_6O_3S$	469.10774	469.10790	1.60×10^{-4}	0.34107
**7a**	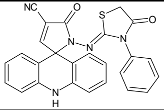	*5'-oxo-1'-{[(2Z)-4-oxo-3-phenyl-1,3-thiazolidin-2-ylidene]amino}-1',5'-dihydro-10H-spiro[acridine-9,2'-pyrrole]-4'-carbonitrile*	$C_{26}H_{17}N_5O_2S$	464.11757	464.11790	3.30×10^{-4}	0.71103
**7b**	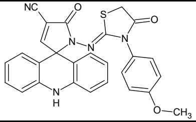	*1'-{[(2Z)-3-(4-methoxyphenyl)-4-oxo-1,3-thiazolidin-2-ylidene]amino}-5'-oxo-1',5'-dihydro-10H-spiro[acridine-9,2'-pyrrole]-4'-carbonitrile*	$C_{27}H_{19}N_5O_3S$	494.12814	494.12880	6.60×10^{-4}	1.33569
**7c**	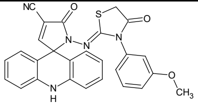	*1'-{[(2Z)-3-(3-methoxyphenyl)-4-oxo-1,3-thiazolidin-2-ylidene]amino}-5'-oxo-1',5'-dihydro-10H-spiro[acridine-9,2'-pyrrole]-4'-carbonitrile*	$C_{27}H_{19}N_5O_3S$	494.12814	494.12880	6.60×10^{-4}	1.33569
**7d**	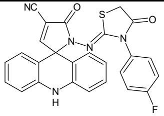	*1'-{[(2Z)-3-(4-fluorophenyl)-4-oxo-1,3-thiazolidin-2-ylidene]amino}-5'-oxo-1',5'-dihydro-10H-spiro[acridine-9,2'-pyrrole]-4'-carbonitrile*	$C_{26}H_{16}FN_5O_2S$	482.10815	482.10870	5.50×10^{-4}	1.14082
**7e**	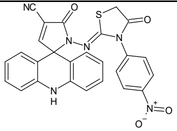	*1'-{[(2Z)-3-(4-nitrophenyl)-4-oxo-1,3-thiazolidin-2-ylidene]amino}-5'-oxo-1',5'-dihydro-10H-spiro[acridine-9,2'-pyrrole]-4'-carbonitrile*	$C_{26}H_{16}N_6O_4S$	509.10265	509.10330	6.50×10^{-4}	1.27676

**8a**	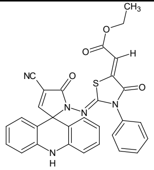	*ethyl 2-[(2Z,5Z)-2-({4'-cyano-5'-oxo-1',5'-dihydro-10H-spiro[acridine-9,2'-pyrrol]-1'-yl}imino)-4-oxo-3-phenyl-1,3-thiazolidin-5-ylidene]acetate*	$C_{30}H_{21}N_5O_4S$	548.13870	548.13920	5.00×10^{-4}	0.91218
**8b**	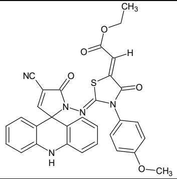	*ethyl 2-[(2Z,5Z)-2-({4'-cyano-5'-oxo-1',5'-dihydro-10H-spiro[acridine-9,2'-pyrrol]-1'-yl}imino)-3-(4-methoxyphenyl)-4-oxo-1,3-thiazolidin-5-ylidene]acetate*	$C_{31}H_{23}N_5O_5S$	578.14927	578.15000	7.30×10^{-4}	1.26265
**8c**	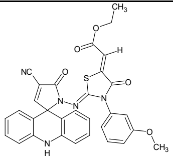	*ethyl 2-[(2Z,5Z)-2-({4'-cyano-5'-oxo-1',5'-dihydro-10H-spiro[acridine-9,2'-pyrrol]-1'-yl}imino)-3-(3-methoxyphenyl)-4-oxo-1,3-thiazolidin-5-ylidene]acetate*	$C_{31}H_{23}N_5O_5S$	578.14927	578.15000	7.30×10^{-4}	1.26265
**8d**	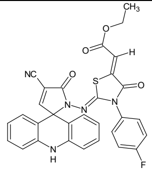	*ethyl 2-[(2Z,5Z)-2-({4'-cyano-5'-oxo-1',5'-dihydro-10H-spiro[acridine-9,2'-pyrrol]-1'-yl}imino)-3-(4-fluorophenyl)-4-oxo-1,3-thiazolidin-5-ylidene]acetate*	$C_{30}H_{20}FN_5O_4S$	566.12928	566.13000	7.20×10^{-4}	1.27179
**8e**	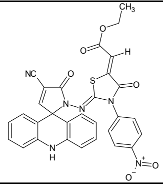	*ethyl 2-[(2Z,5Z)-2-({4'-cyano-5'-oxo-1',5'-dihydro-10H-spiro[acridine-9,2'-pyrrol]-1'-yl}imino)-3-(4-nitrophenyl)-4-oxo-1,3-thiazolidin-5-ylidene]acetate*	$C_{30}H_{20}N_6O_6S$	593.12378	593.12410	3.20×10^{-4}	0.53952
**9a**	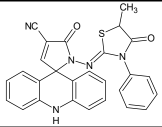	*1'-{[(2Z)-5-methyl-4-oxo-3-phenyl-1,3-thiazolidin-2-ylidene]amino}-5'-oxo-1',5'-dihydro-10H-spiro[acridine-9,2'-pyrrole]-4'-carbonitrile*	$C_{27}H_{19}N_5O_2S$	478.13322	478.13410	8.80×10^{-4}	1.84049
**9b**	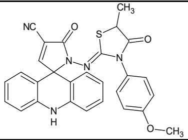	*1'-{[(2Z)-3-(4-methoxyphenyl)-5-methyl-4-oxo-1,3-thiazolidin-2-ylidene]amino}-5'-oxo-1',5'-dihydro-10H-spiro[acridine-9,2'-pyrrole]-4'-carbonitrile*	$C_{28}H_{21}N_5O_3S$	508.14379	508.14420	4.10×10^{-4}	0.80686
**9c**	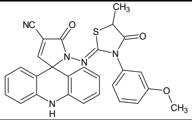	*1'-{[(2Z)-3-(3-methoxyphenyl)-5-methyl-4-oxo-1,3-thiazolidin-2-ylidene]amino}-5'-oxo-1',5'-dihydro-10H-spiro[acridine-9,2'-pyrrole]-4'-carbonitrile*	$C_{28}H_{21}N_5O_3S$	508.14379	508.14450	7.10×10^{-4}	1.39724

**9d**	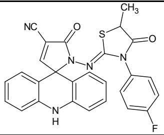	*1'-{[(2Z)-3-(4-fluorophenyl)-5-methyl-4-oxo-1,3-thiazolidin-2-ylidene]amino}-5'-oxo-1',5'-dihydro-10H-spiro[acridine-9,2'-pyrrole]-4'-carbonitrile*	$C_{27}H_{18}FN_5O_2S$	496.12380	496.12470	9.00×10^{-4}	1.81406
**9e**	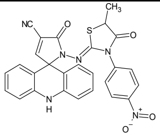	*1'-{[(2Z)-5-methyl-3-(4-nitrophenyl)-4-oxo-1,3-thiazolidin-2-ylidene]amino}-5'-oxo-1',5'-dihydro-10H-spiro[acridine-9,2'-pyrrole]-4'-carbonitrile*	$C_{27}H_{18}N_6O_4S$	523.11830	523.11880	5.00×10^{-4}	0.95581
**10a**	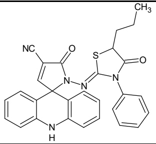	*5'-oxo-1'-{[(2Z)-4-oxo-3-phenyl-5-propyl-1,3-thiazolidin-2-ylidene]amino}-1',5'-dihydro-10H-spiro[acridine-9,2'-pyrrole]-4'-carbonitrile*	$C_{29}H_{23}N_5O_2S$	506.16452	506.16510	5.80×10^{-4}	1.14587
**10b**	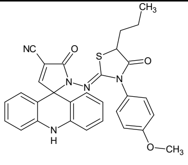	*1'-{[(2Z)-3-(4-methoxyphenyl)-4-oxo-5-propyl-1,3-thiazolidin-2-ylidene]amino}-5'-oxo-1',5'-dihydro-10H-spiro[acridine-9,2'-pyrrole]-4'-carbonitrile*	$C_{30}H_{25}N_5O_3S$	536.17509	536.17560	5.10×10^{-4}	0.95118
**10c**	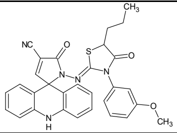	*1'-{[(2Z)-3-(3-methoxyphenyl)-4-oxo-5-propyl-1,3-thiazolidin-2-ylidene]amino}-5'-oxo-1',5'-dihydro-10H-spiro[acridine-9,2'-pyrrole]-4'-carbonitrile*	$C_{30}H_{25}N_5O_3S$	536.17509	536.17570	6.10×10^{-4}	1.13769
**10d**	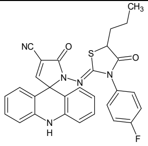	*1'-{[(2Z)-3-(4-fluorophenyl)-4-oxo-5-propyl-1,3-thiazolidin-2-ylidene]amino}-5'-oxo-1',5'-dihydro-10H-spiro[acridine-9,2'-pyrrole]-4'-carbonitrile*	$C_{29}H_{22}FN_5O_2S$	524.15510	524.15610	1.00×10^{-4}	1.90783
**10 e**	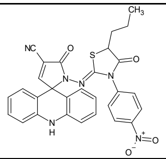	*1'-{[(2Z)-3-(4-nitrophenyl)-4-oxo-5-propyl-1,3-thiazolidin-2-ylidene]amino}-5'-oxo-1',5'-dihydro-10H-spiro[acridine-9,2'-pyrrole]-4'-carbonitrile*	$C_{29}H_{22}N_6O_4S$	551.14960	551.15050	9.00×10^{-4}	1.63295

2 NMR, IR and MS spectra of synthesized derivatives

2.1 3-{4'-Cyano-5'-oxo-1',5'-dihydro-10*H*-spiro[acridine-9,2'-pyrrol]-1'-yl}-1-phenylthiourea (6a)

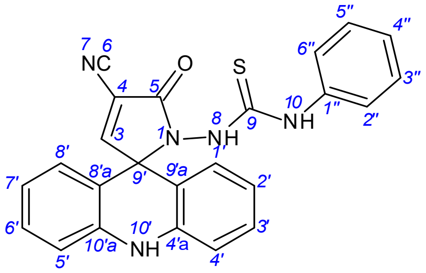

6a

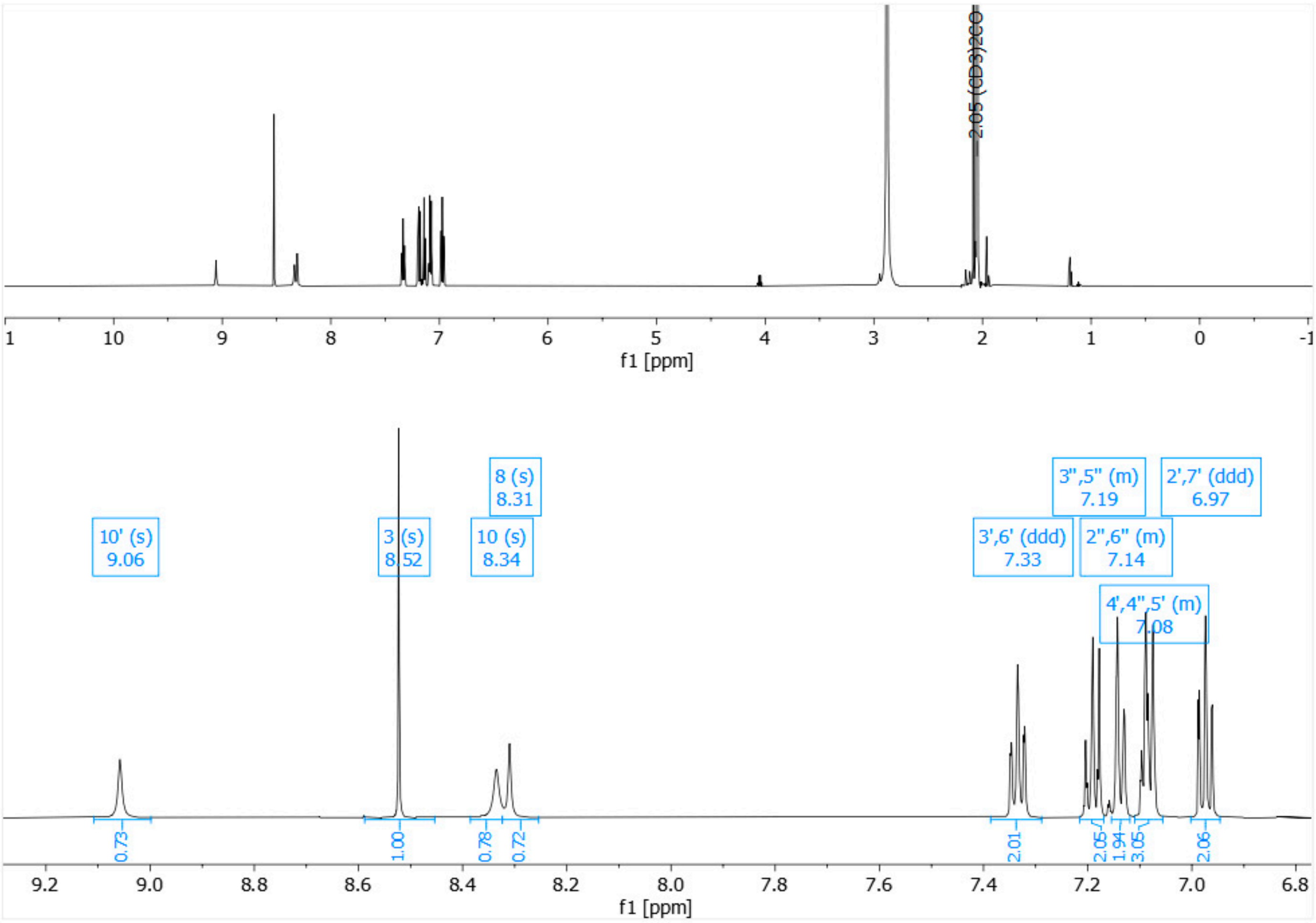

^{1}H NMR (600 MHz, acetone-d$_6$) spectrum of derivative **6a**.

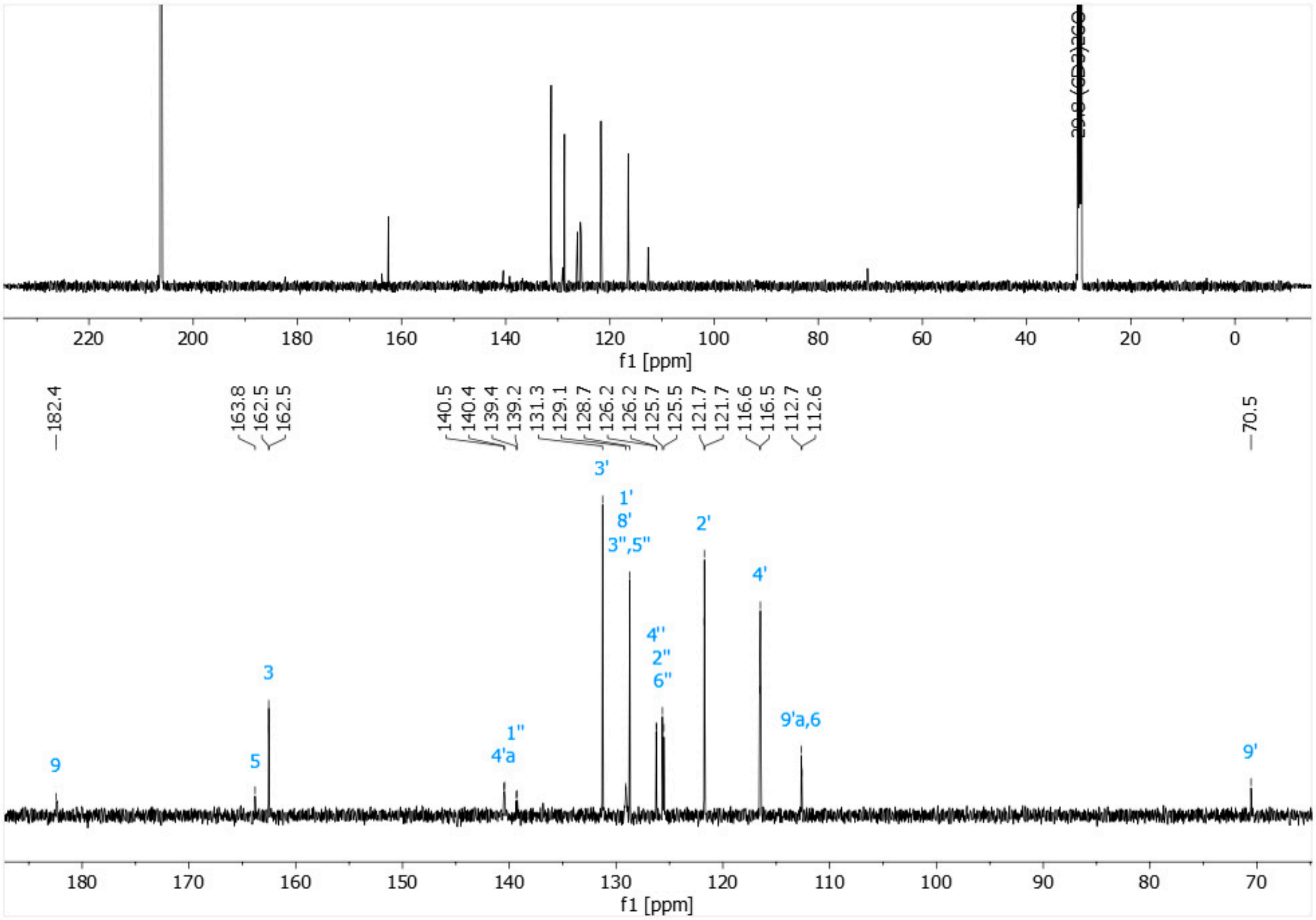

^{13}C NMR (150 MHz, acetone-d_6) spectrum of derivative **6a**.

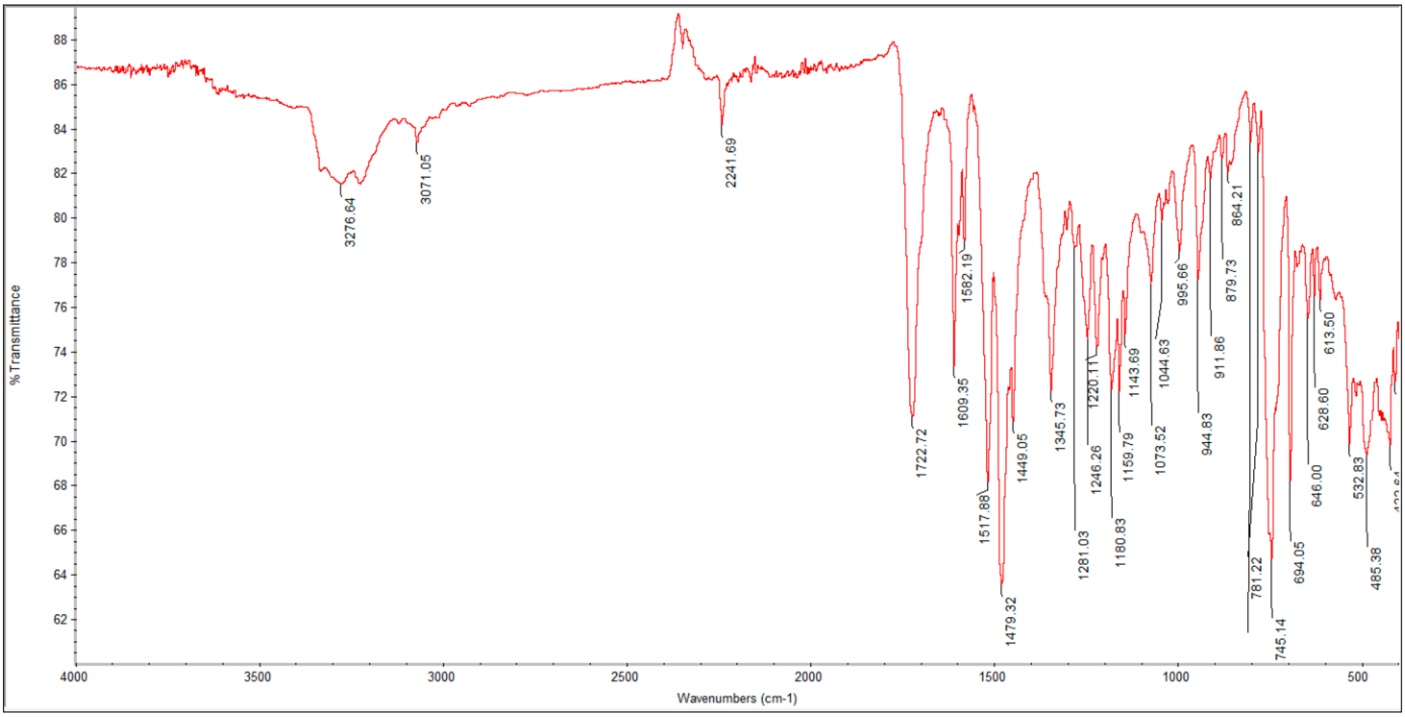

FTIR spectrum of derivative **6a**.

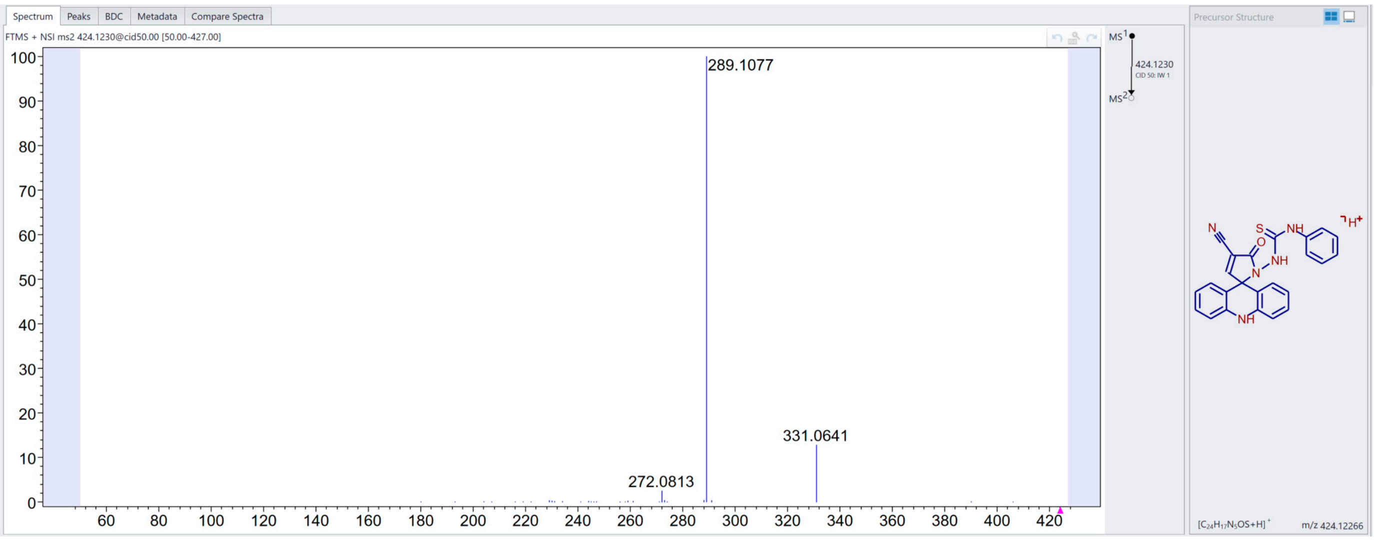

MS2 spectrum of derivative **6a**.

2.2 3-{4'-Cyano-5'-oxo-1',5'-dihydro-10H-spiro[acridine-9,2'-pyrrol]-1'-yl}-1-(4-methoxyphenyl)thiourea (6b)

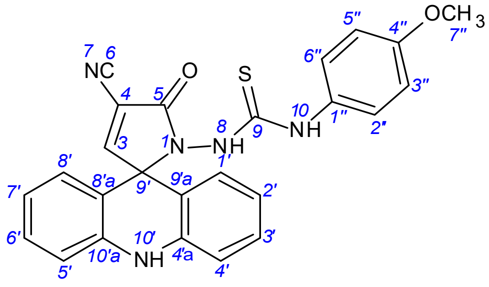

6b

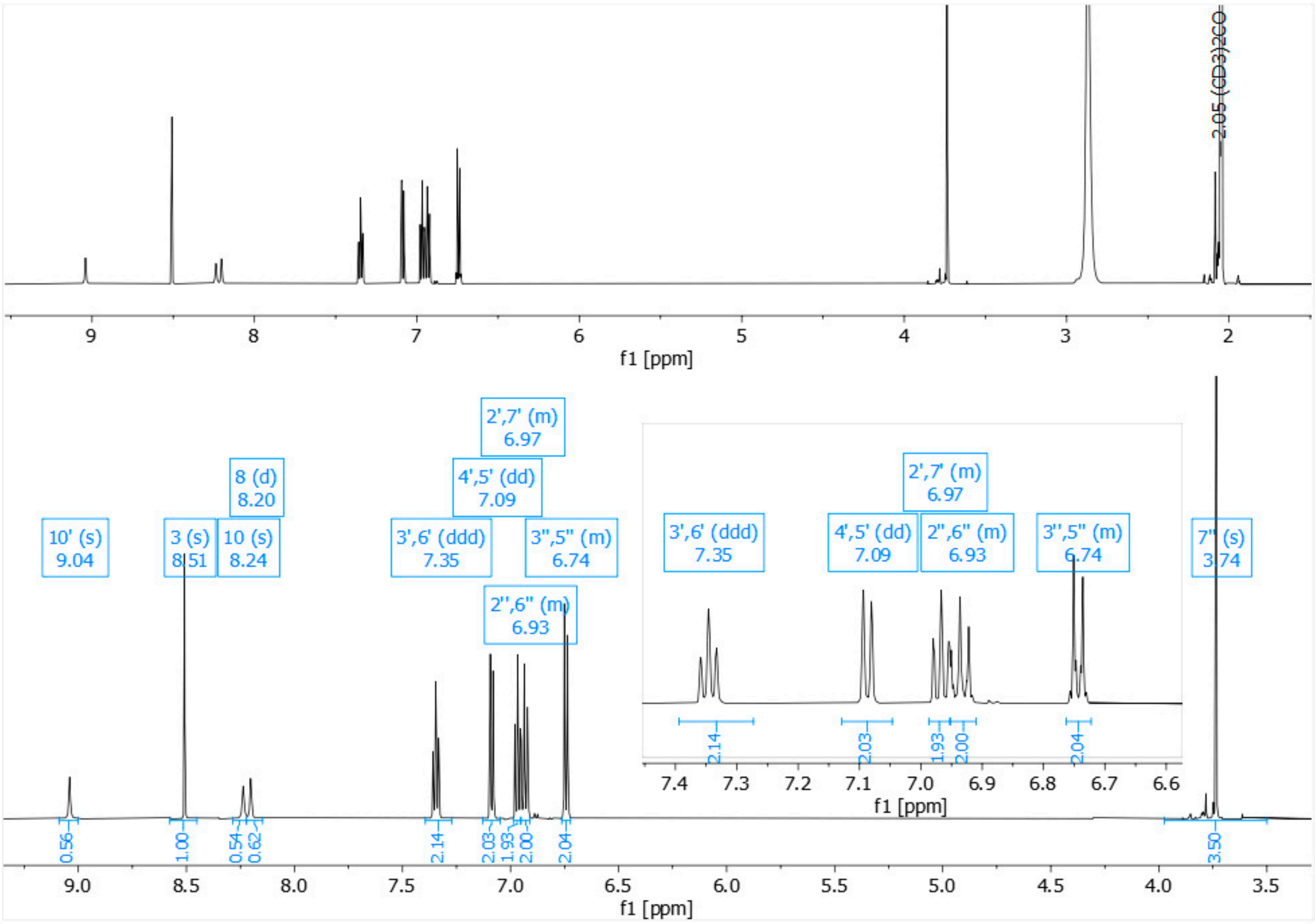

^{1}H NMR (600 MHz, acetone-d$_6$) spectrum of derivative **6b**.

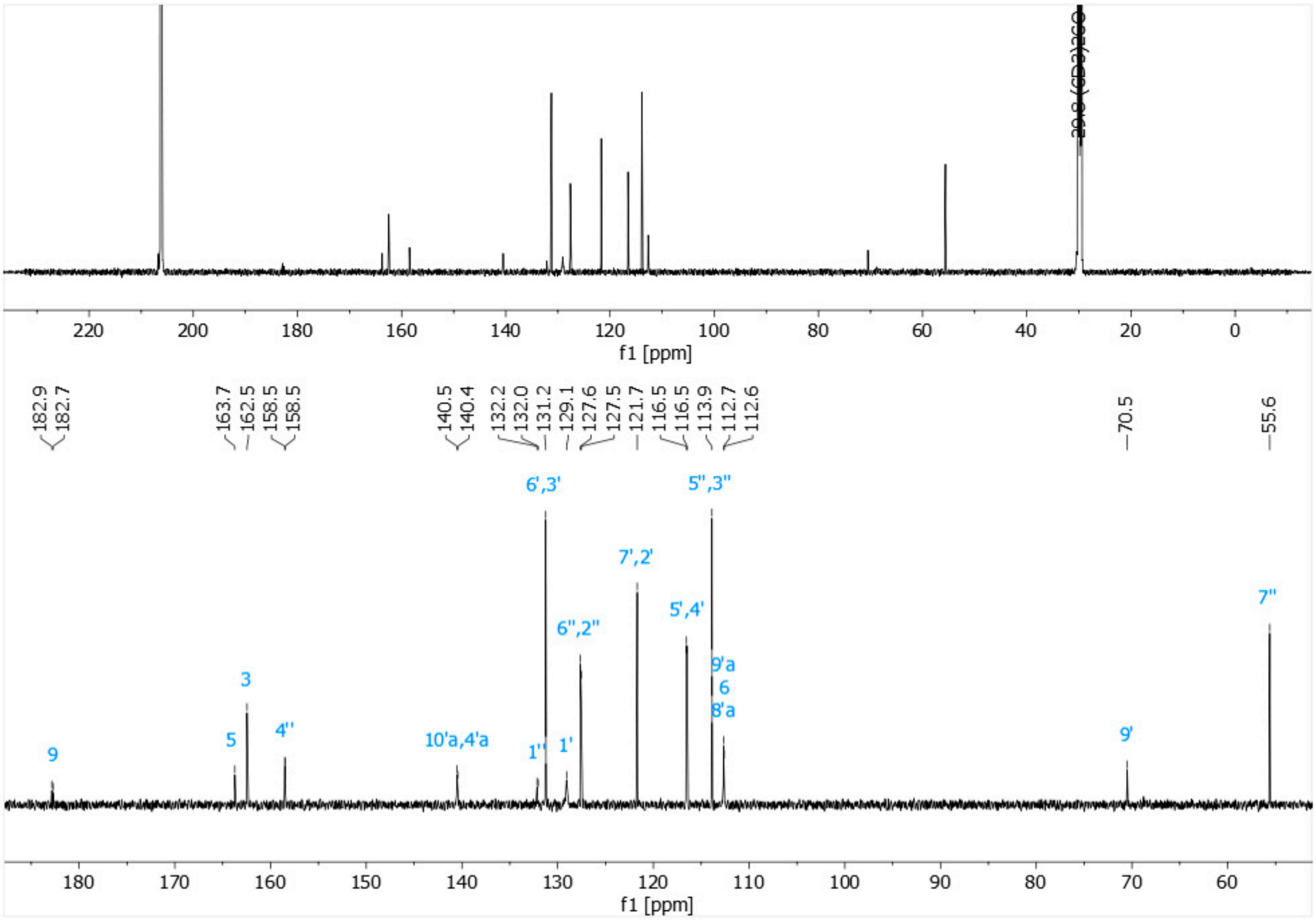

^{13}C NMR (150 MHz, acetone-d$_6$) spectrum of derivative **6b**.

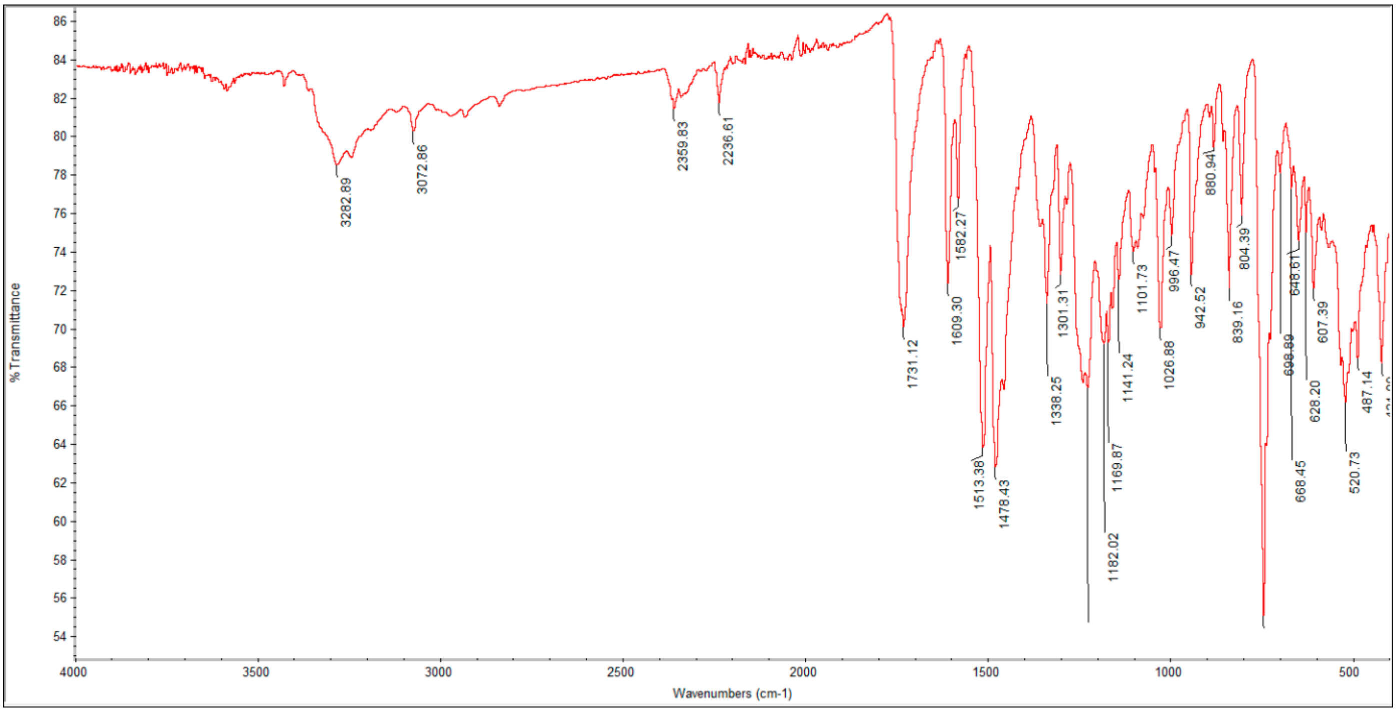

FTIR spectrum of derivative **6b**.

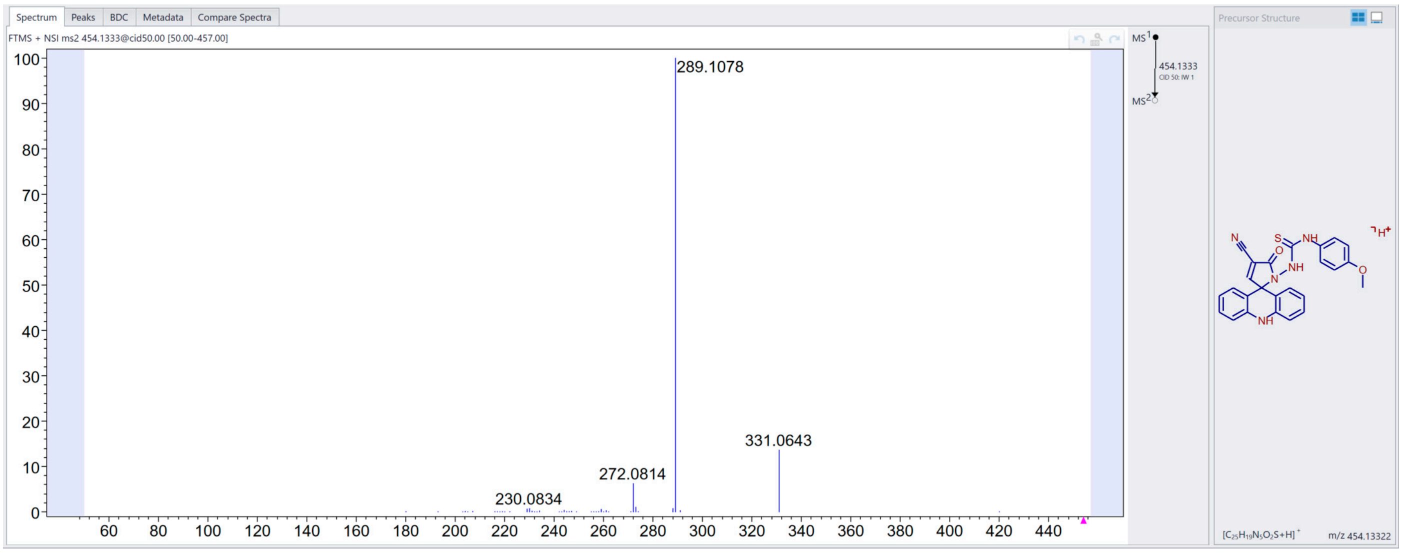

MS2 spectrum of derivative **6b**.

2.3 3-{4'-Cyano-5'-oxo-1',5'-dihydro-10H-spiro[acridine-9,2'-pyrrol]-1'-yl}-1-(3-methoxyphenyl)thiourea (6c)

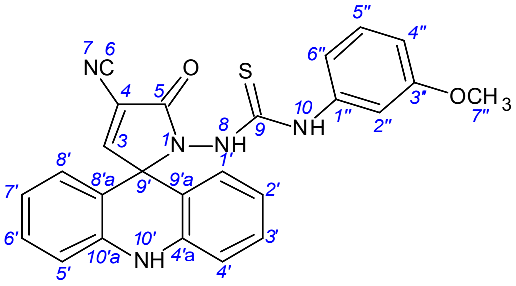

6c

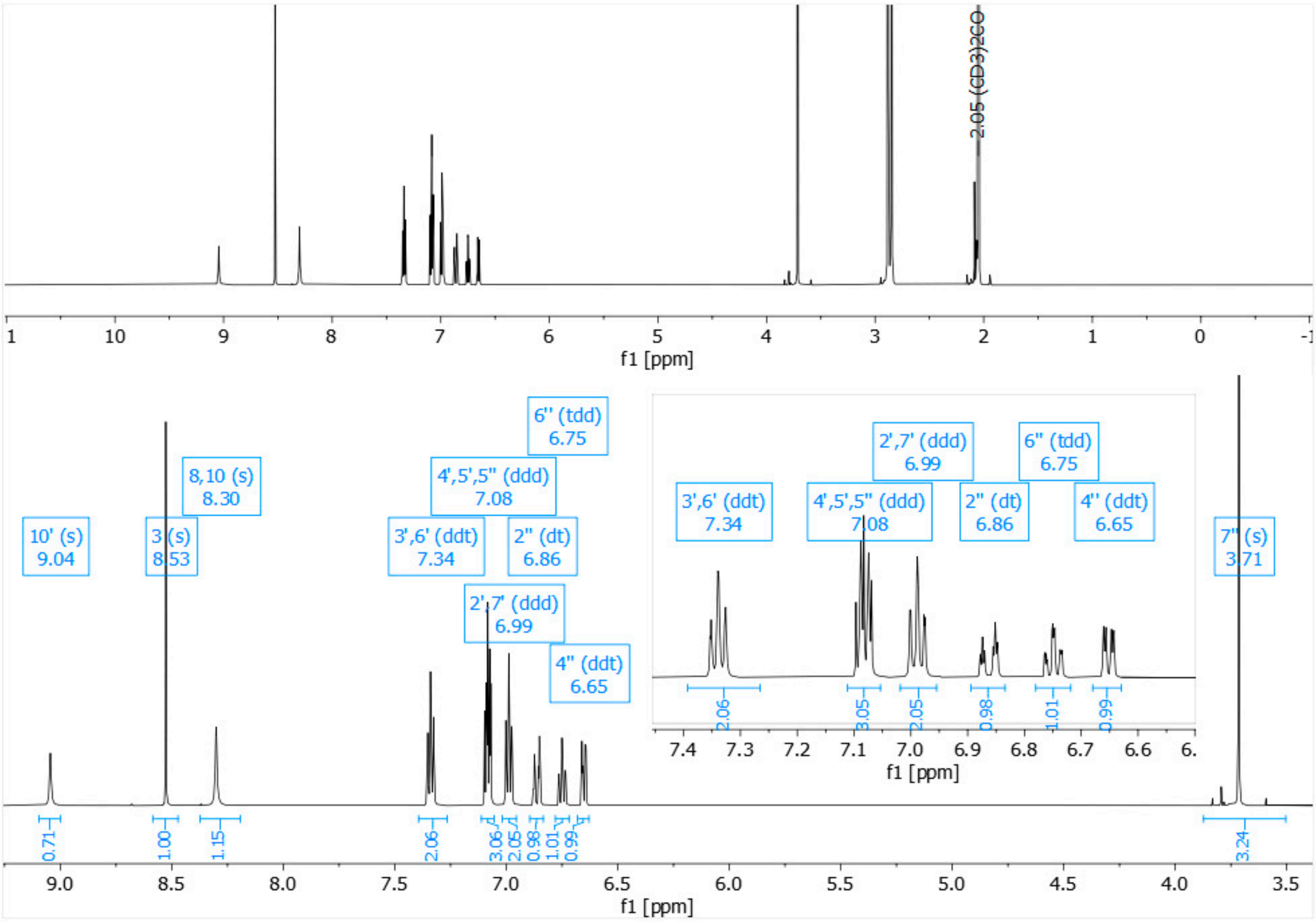

^{1}H NMR (600 MHz, acetone-d_6) spectrum of derivative **6c**.

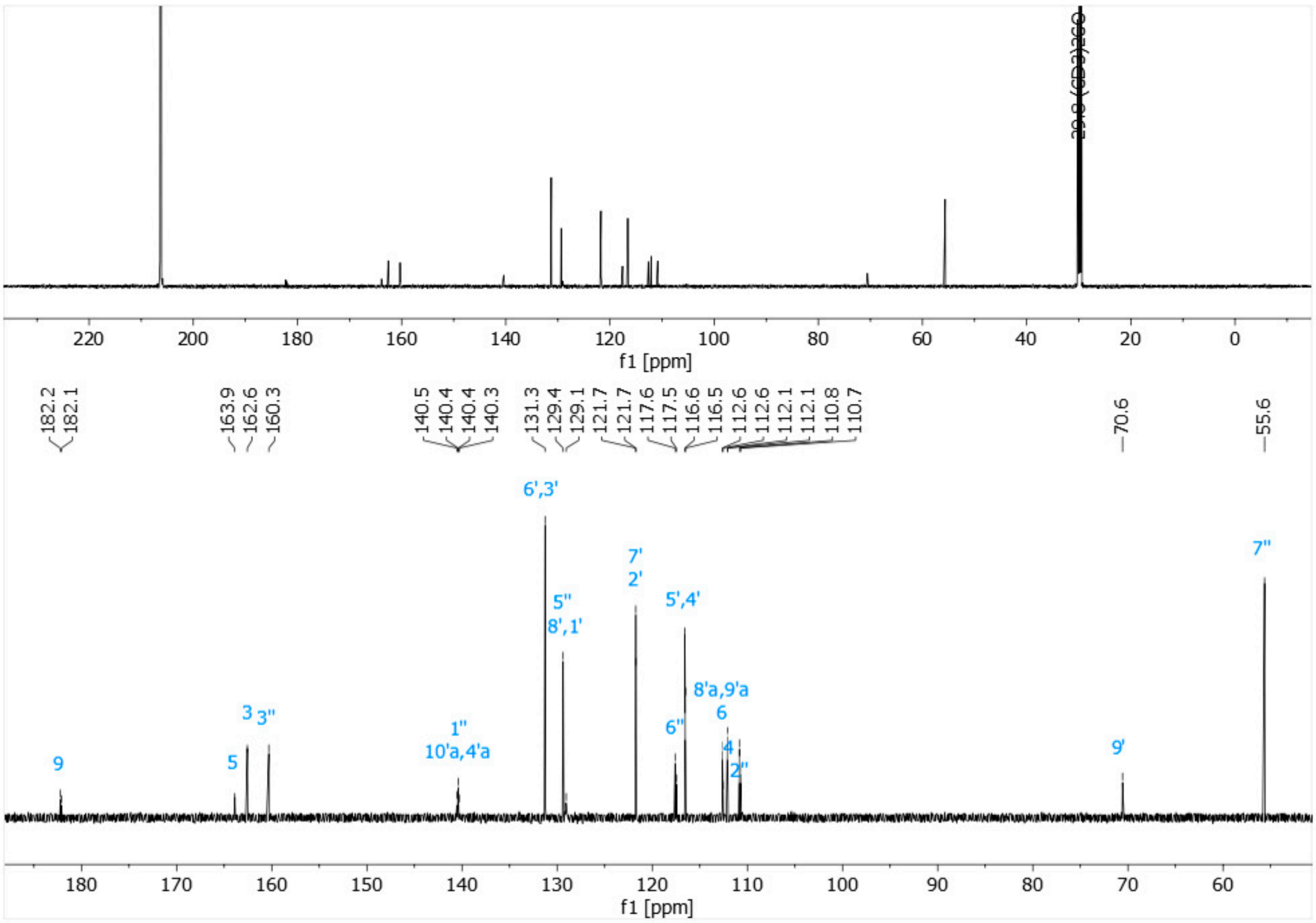

^{13}C NMR (150 MHz, acetone-d$_6$) spectrum of derivative **6c**.

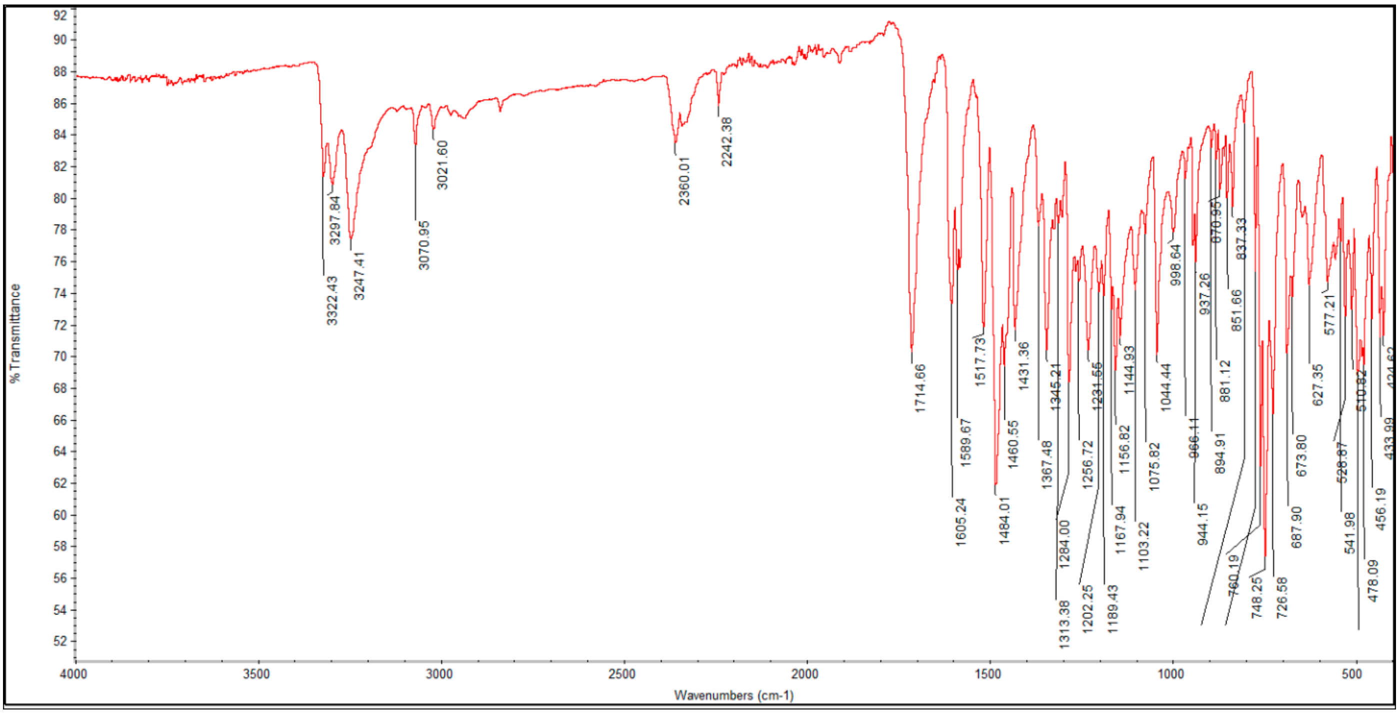

FTIR spectrum of derivative **6c**.

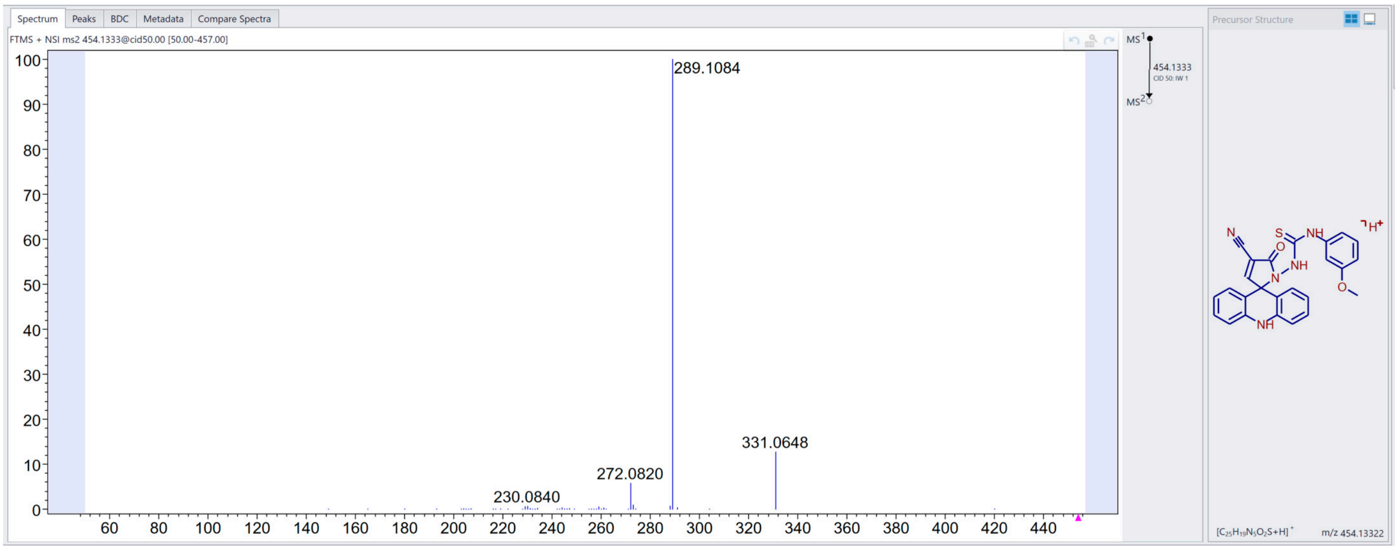

MS2 spectrum of derivative **6c**.

2.4 3-{4'-Cyano-5'-oxo-1',5'-dihydro-10*H*-spiro[acridine-9,2'-pyrrol]-1'-yl}-1-(4-fluorophenyl)thiourea (**6d**)

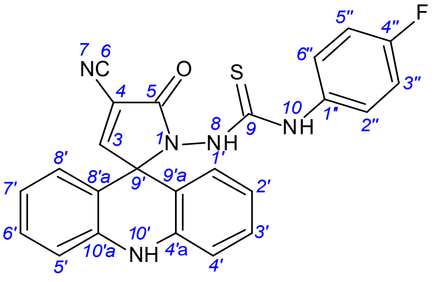

6d

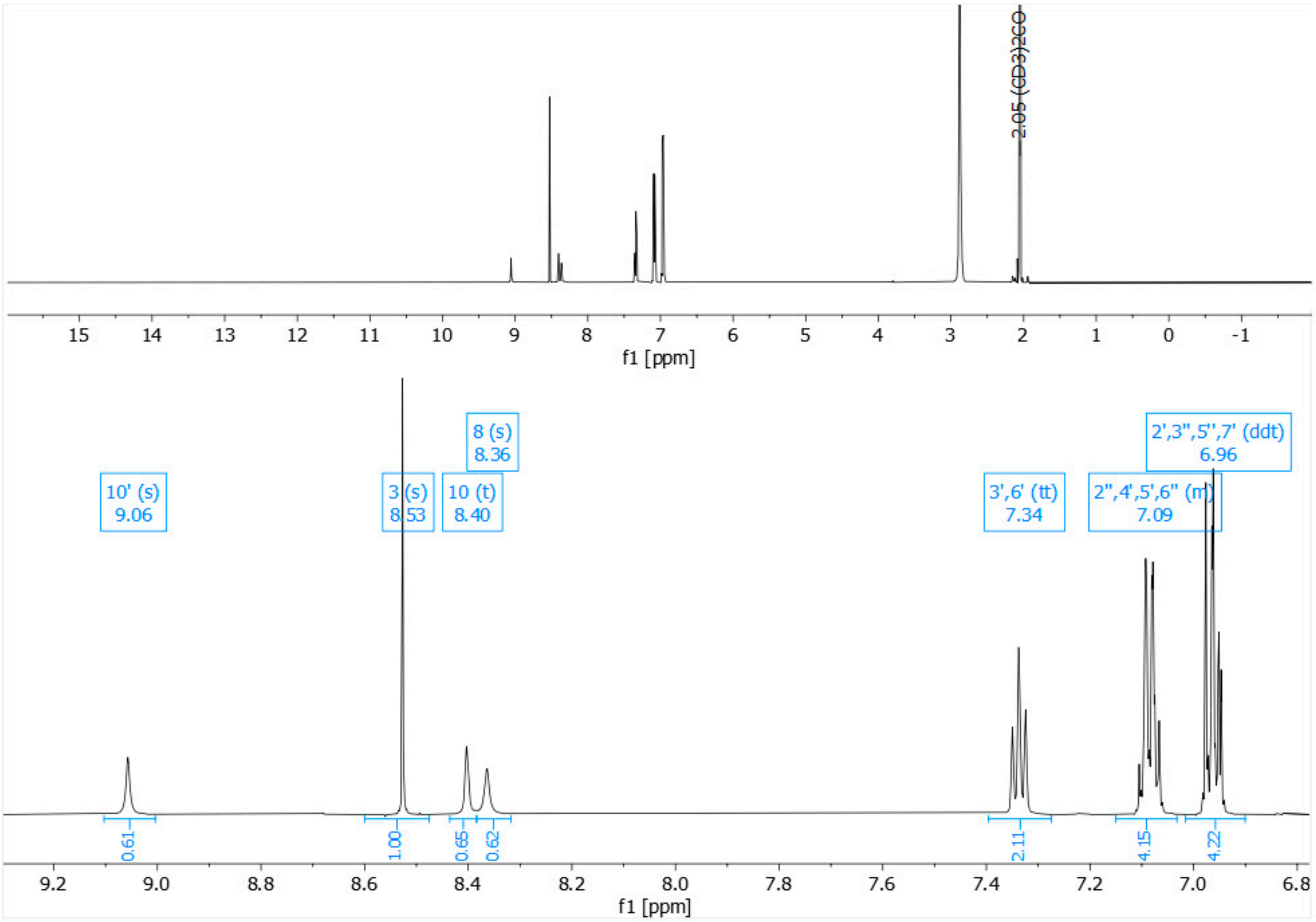

^{1}H NMR (600 MHz, acetone-d$_6$) spectrum of derivative **6d**.

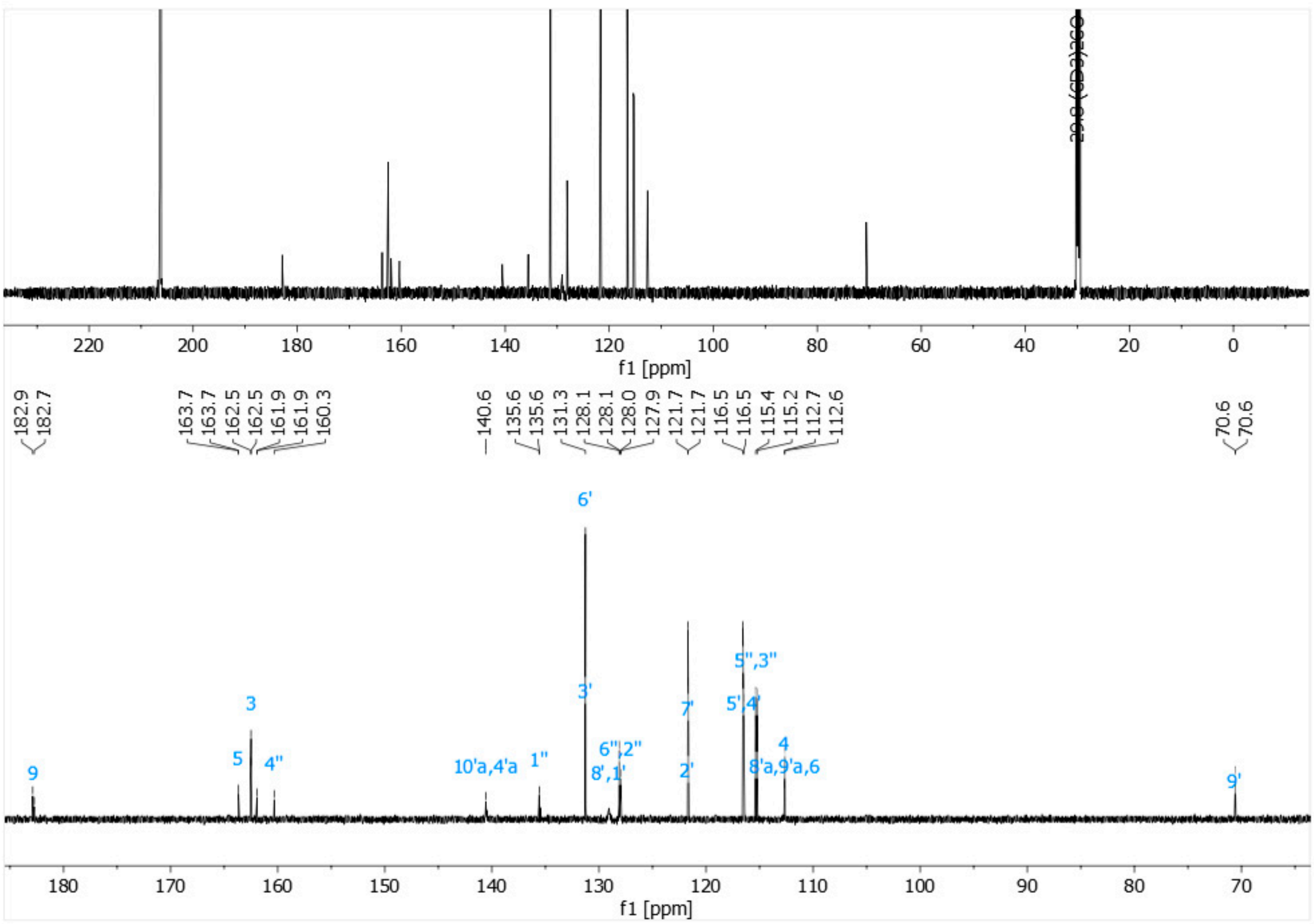

^{13}C NMR (150 MHz, acetone-d$_6$) spectrum of derivative **6d**.

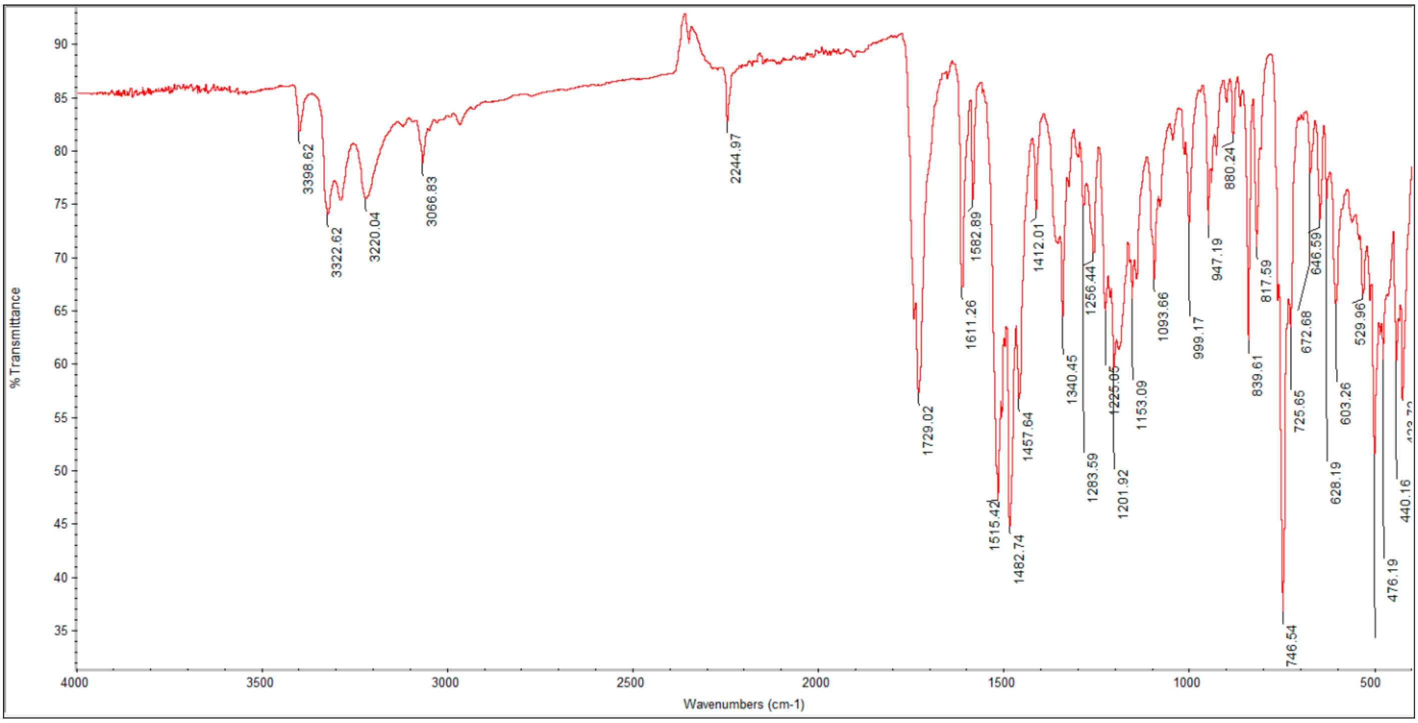

FTIR spectrum of derivative **6d**.

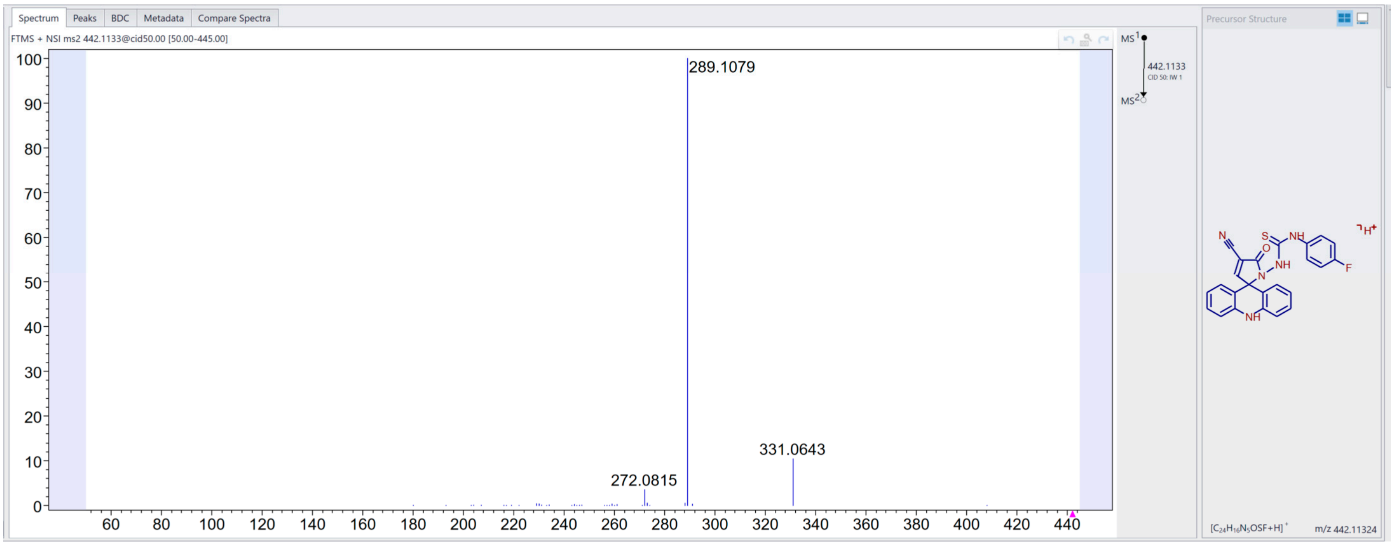

MS2 spectrum of derivative **6d**.

2.5 3-{4'-Cyano-5'-oxo-1',5'-dihydro-10*H*-spiro[acridine-9,2'-pyrrol]-1'-yl}-1-(4-nitrophenyl)thiourea (**6e**)

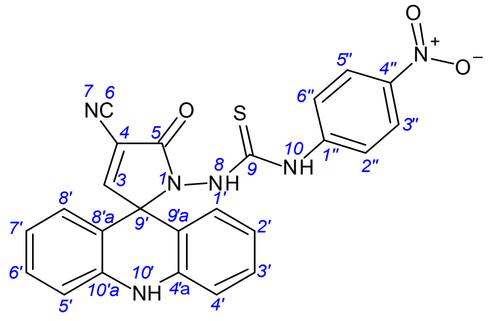

6e

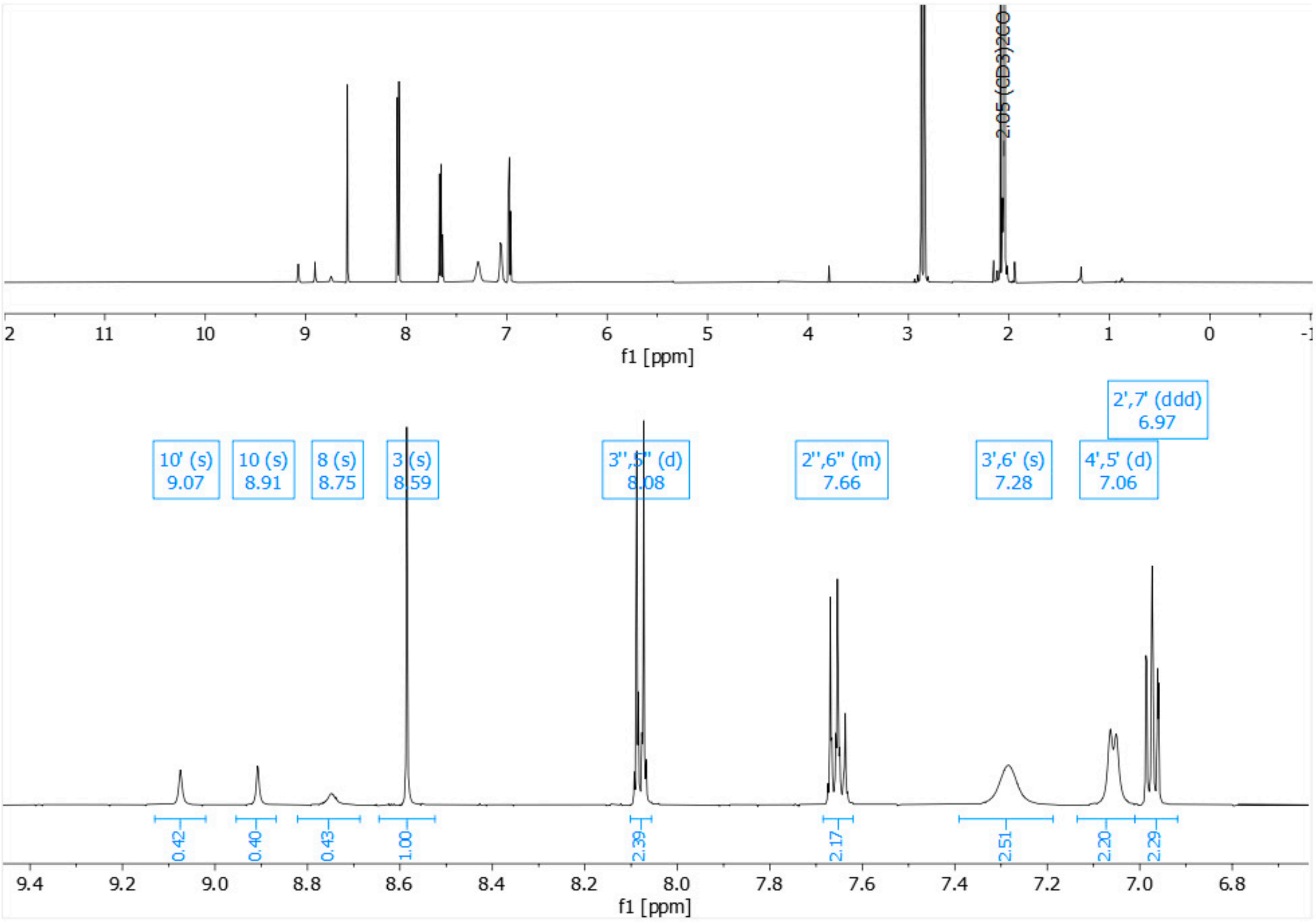

^{1}H NMR (600 MHz, acetone-d$_6$) spectrum of derivative **6e**.

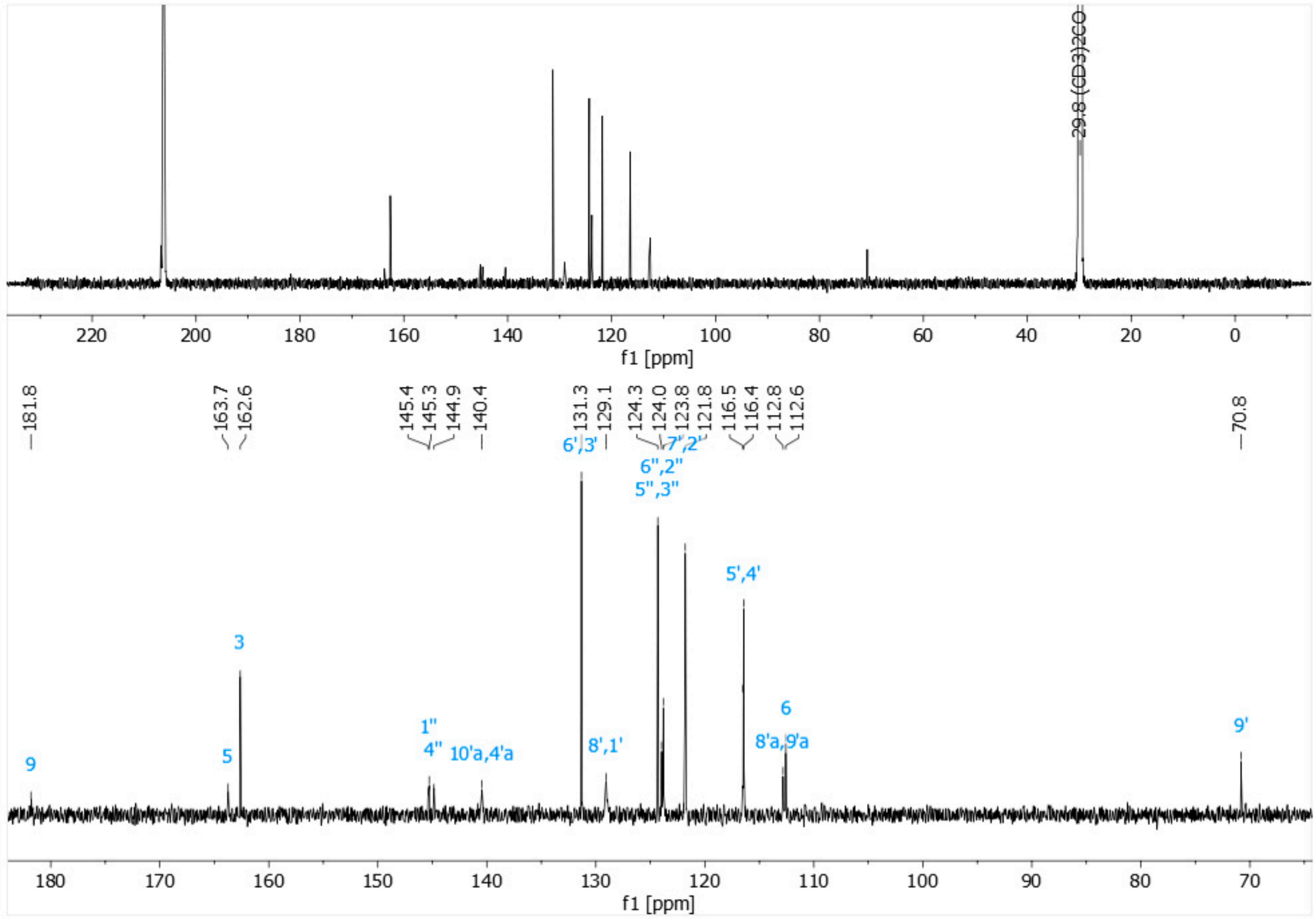

^{13}C NMR (150 MHz, acetone-d_6) spectrum of derivative **6e**.

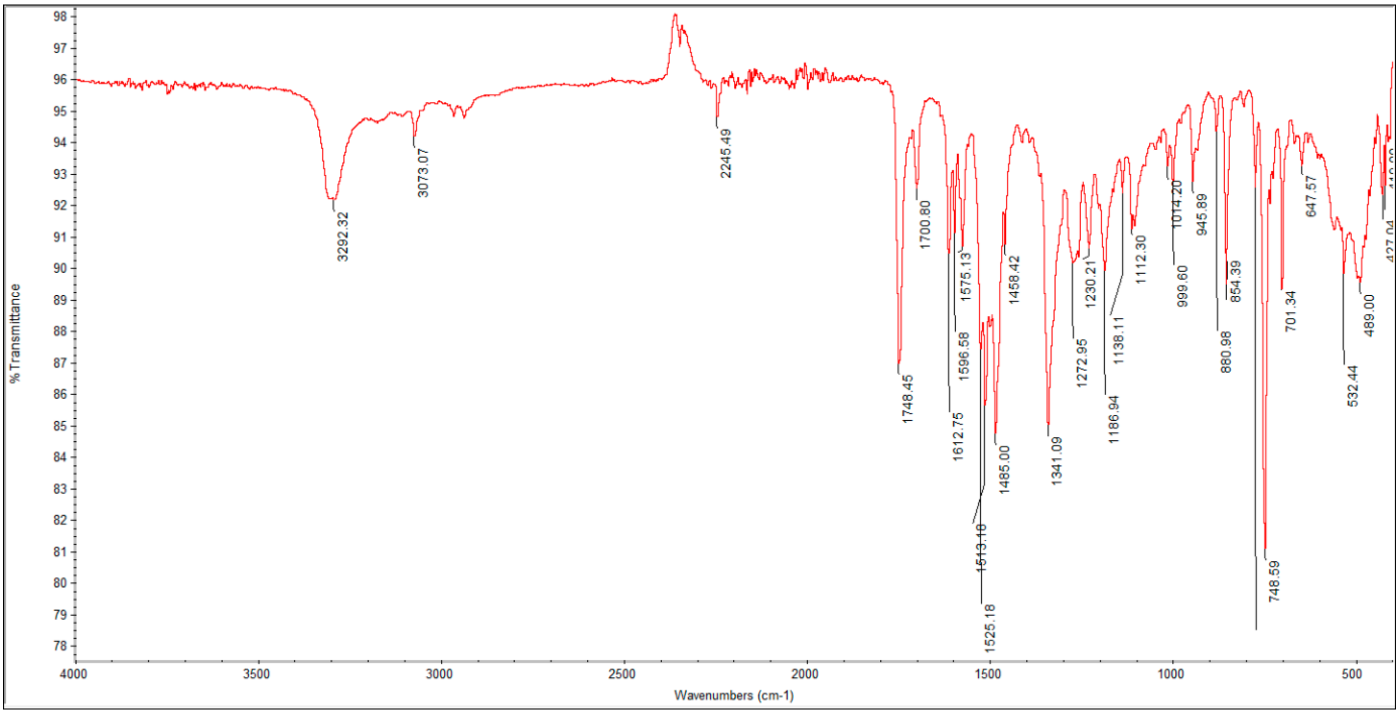

FTIR spectrum of derivative **6e**.

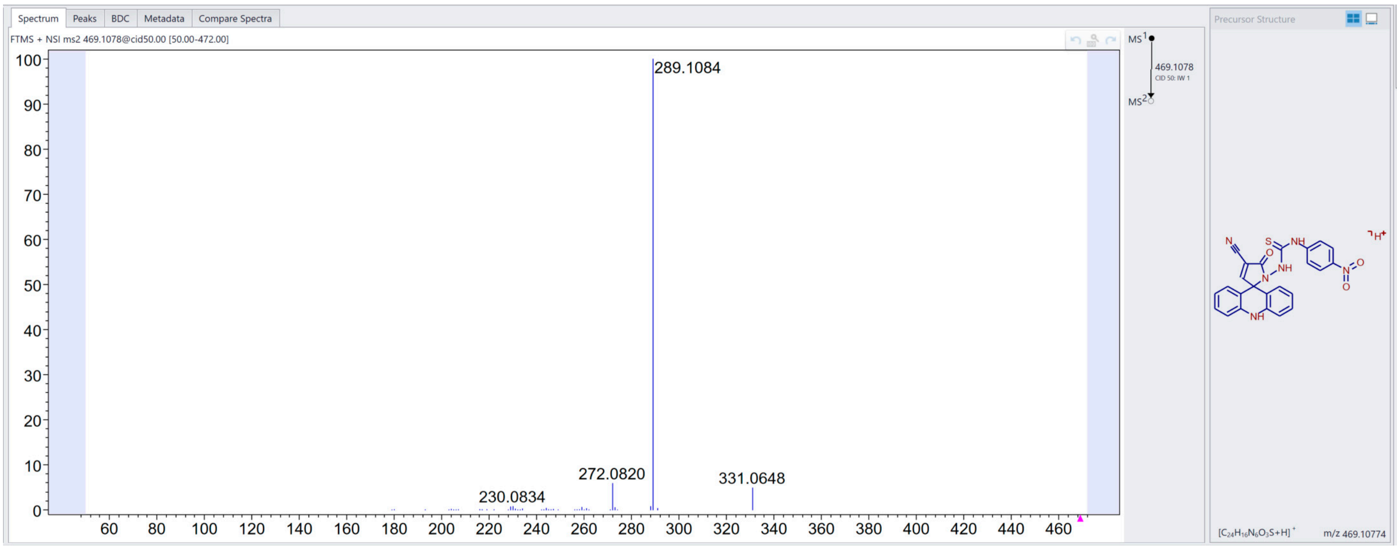

MS2 spectrum of derivative **6e**.

2.6 5'-Oxo-1'-{[(2*Z*)-4-oxo-3-phenyl-1,3-thiazolidin-2-ylidene]amino}-1',5'-dihydro-10*H*-spiro[acridine-9,2'-pyrrole]-4'-carbonitrile (**7a**)

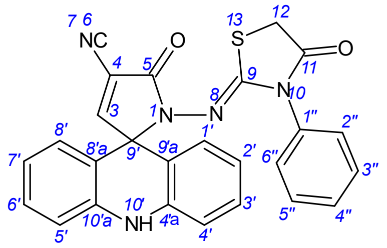

7a

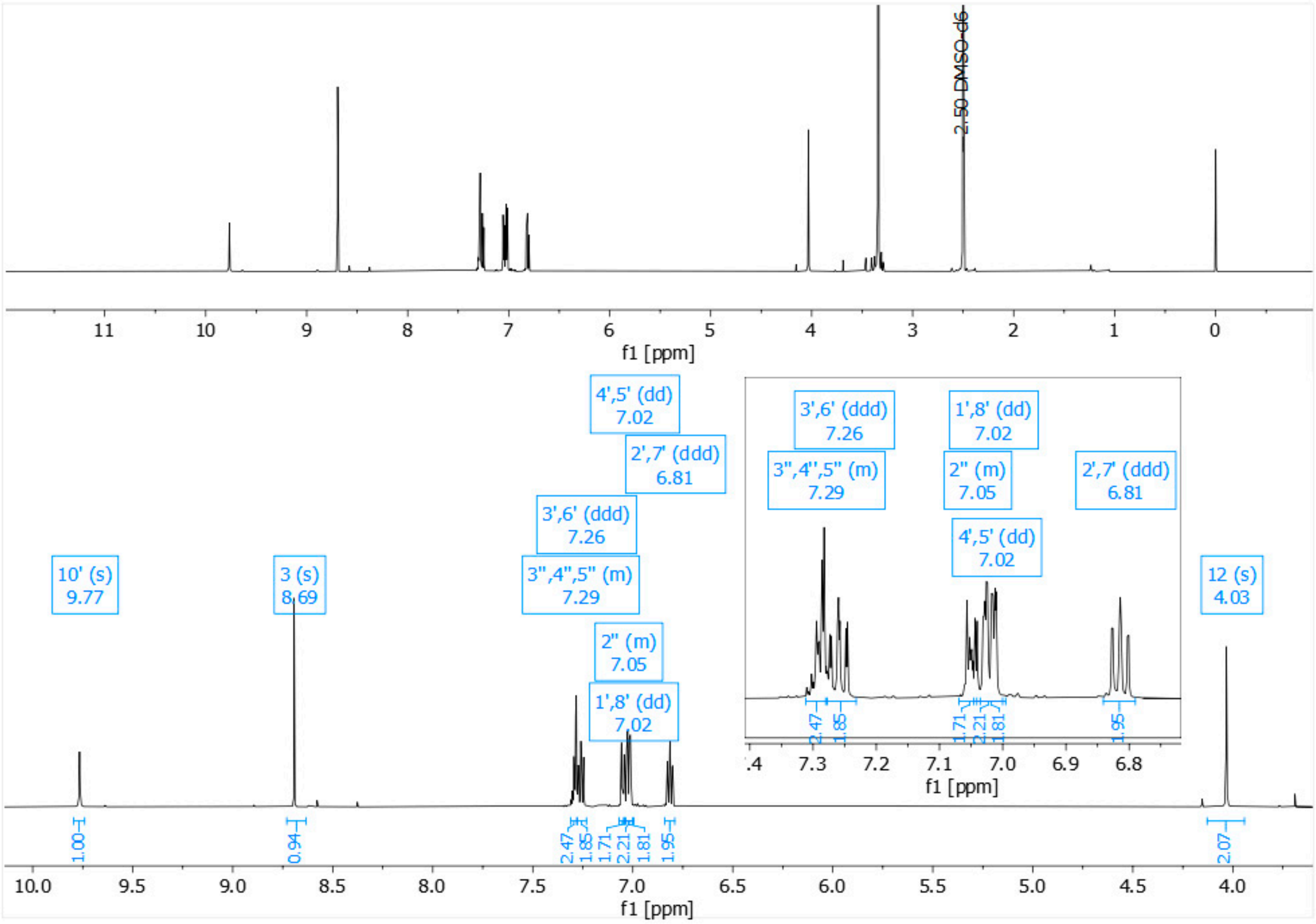

^{1}H NMR (600 MHz, DMSO-d_6) spectrum of derivative **7a**.

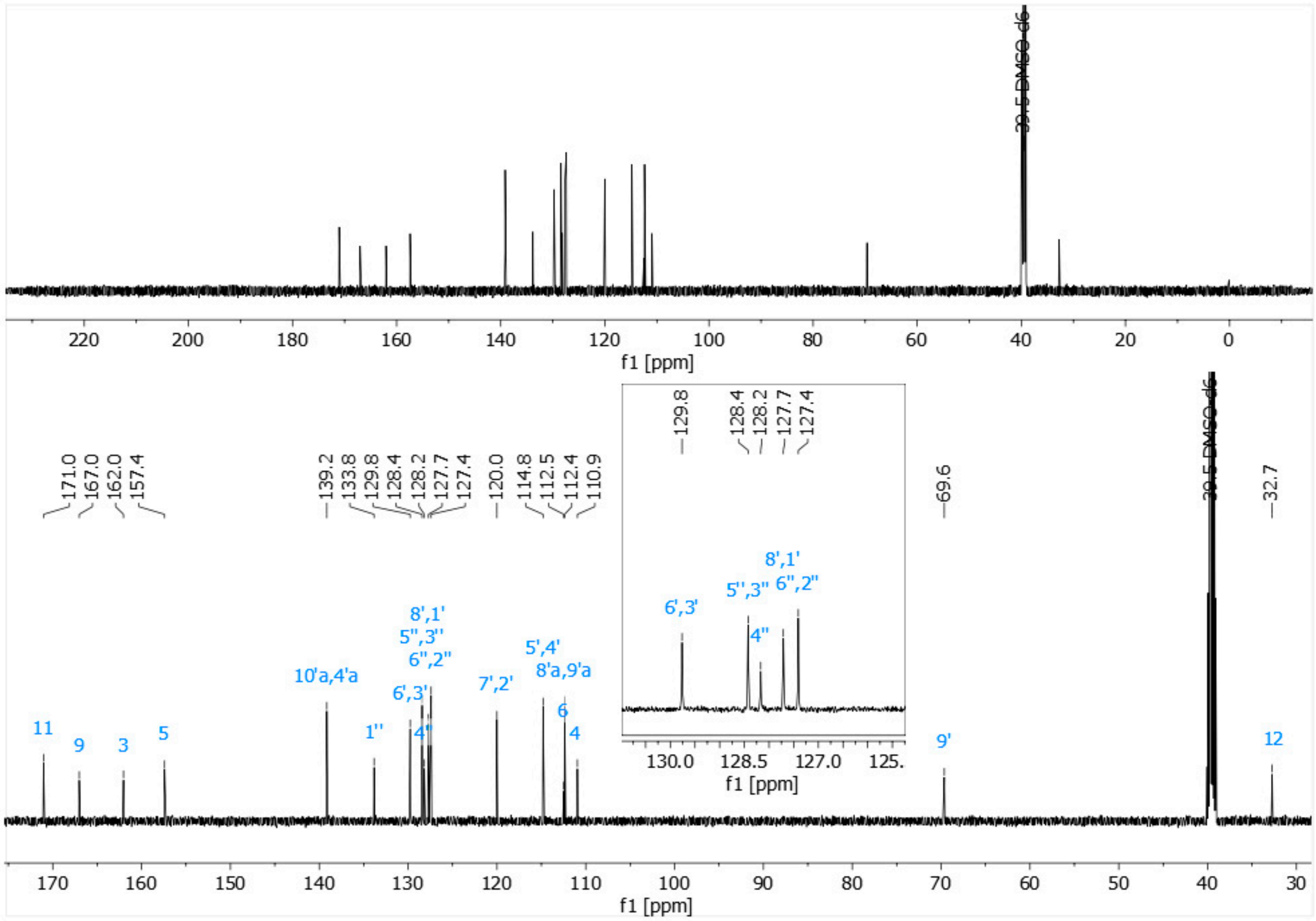

^{13}C NMR (150 MHz, DMSO-d$_6$) spectrum of derivative **7a**.

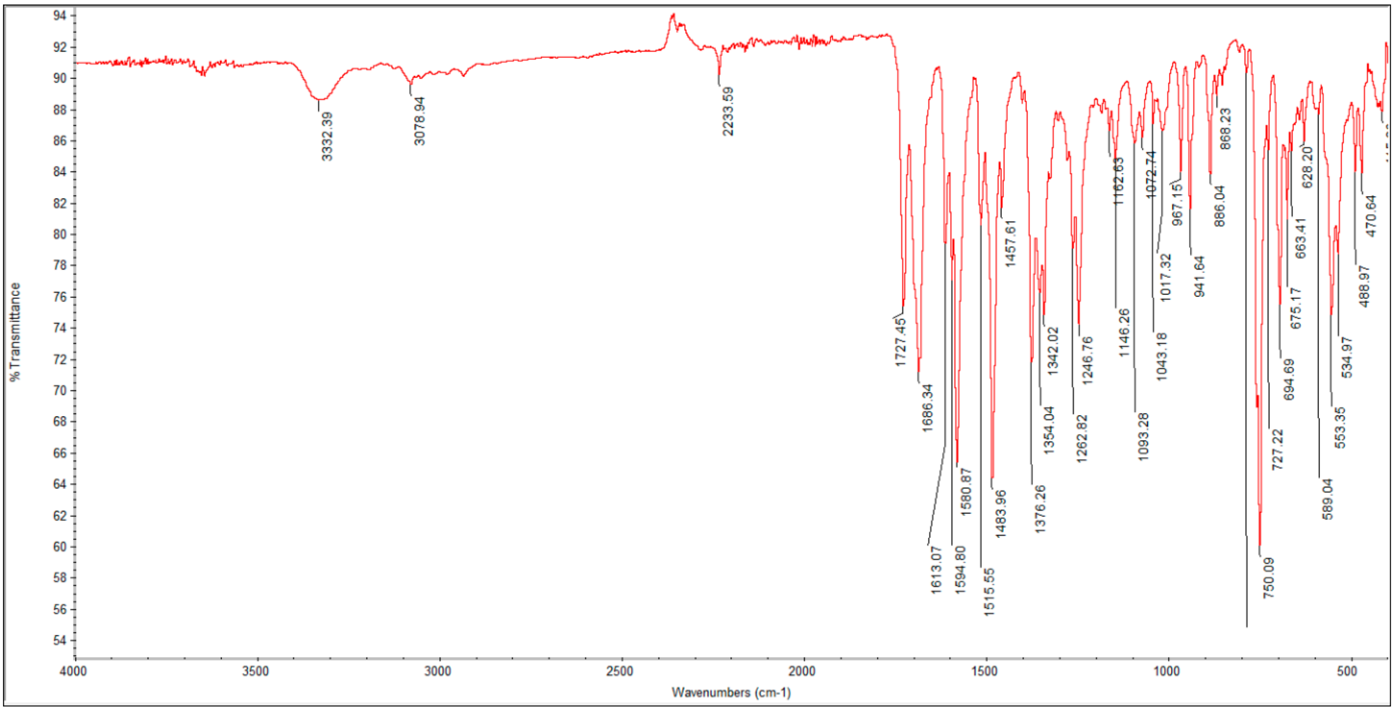

FTIR spectrum of derivative **7a**.

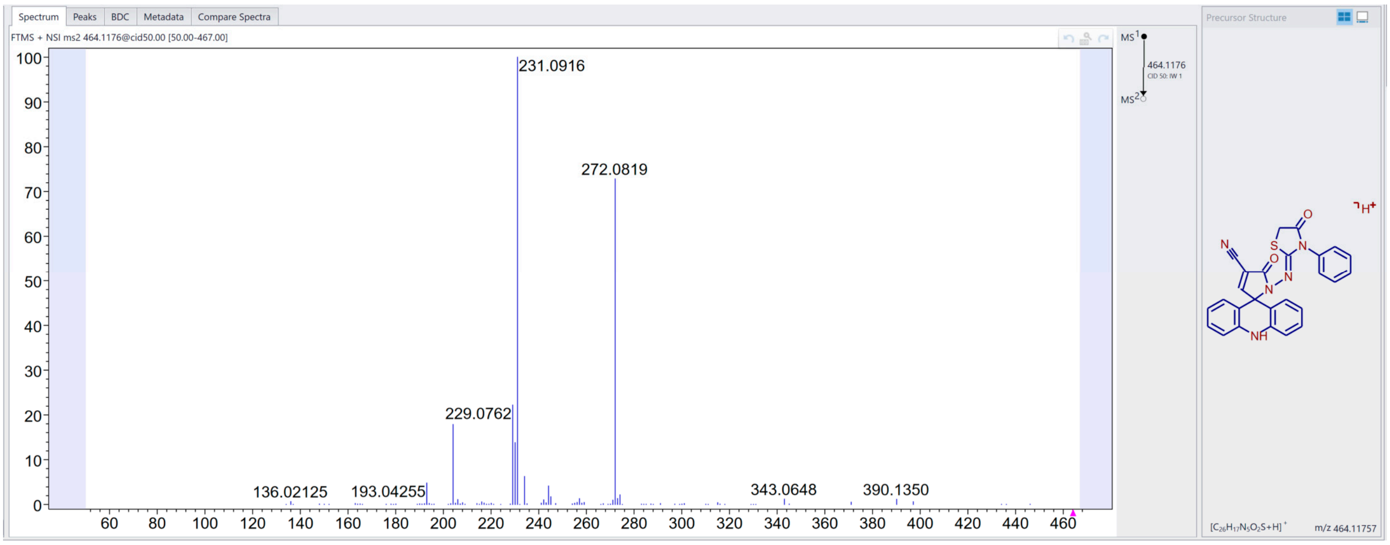

MS2 spectrum of derivative **7a**.

2.7 1'-{[(2*Z*)-3-(4-Methoxyphenyl)-4-oxo-1,3-thiazolidin-2-ylidene]amino}-5'-oxo-1',5'-dihydro-10*H*-spiro[acridine-9,2'-pyrrole]-4'-carbonitrile (**7b**)

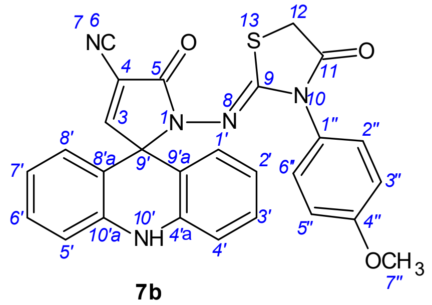

7b

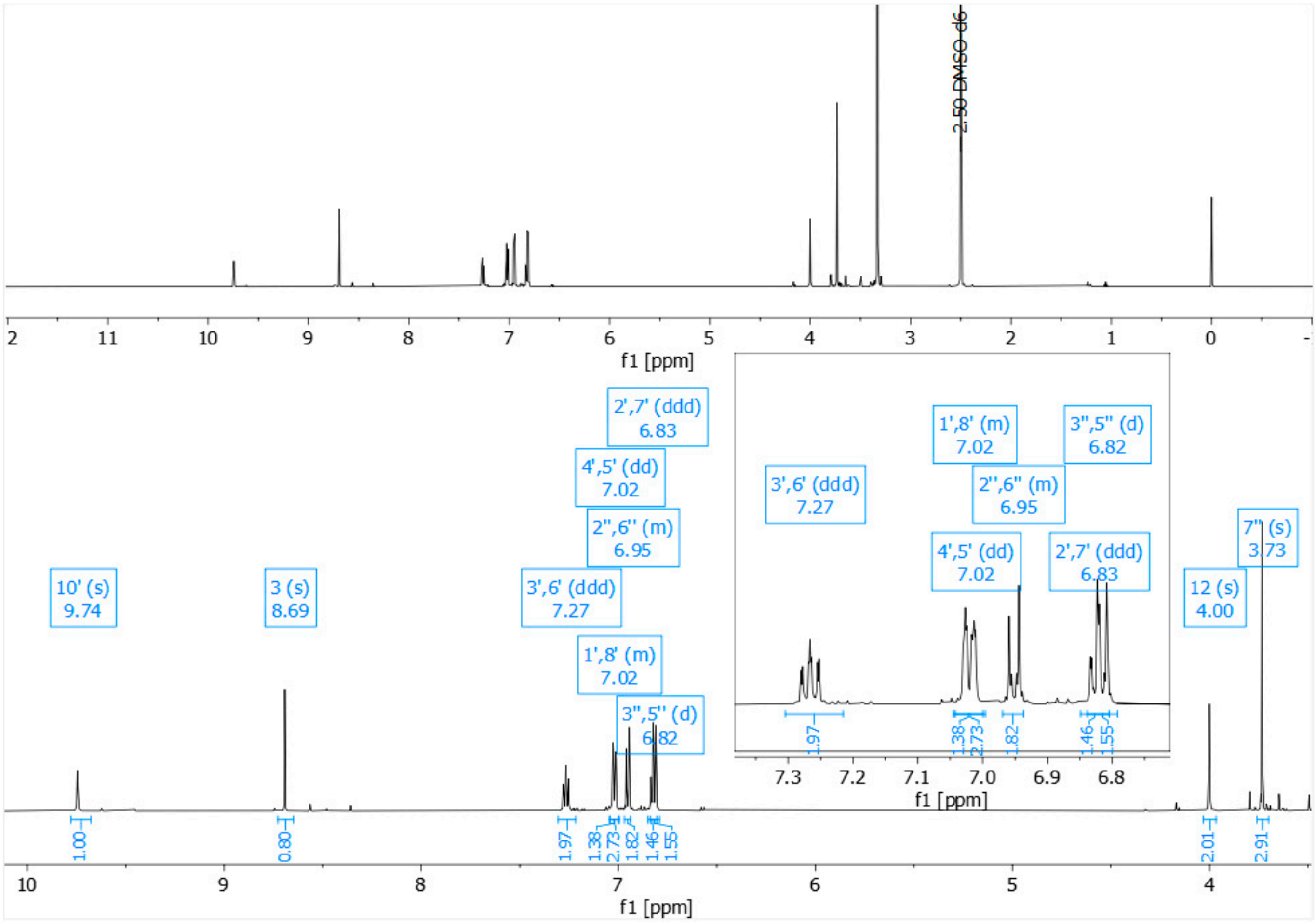

^{1}H NMR (600 MHz, DMSO-d$_6$) spectrum of derivative **7b**.

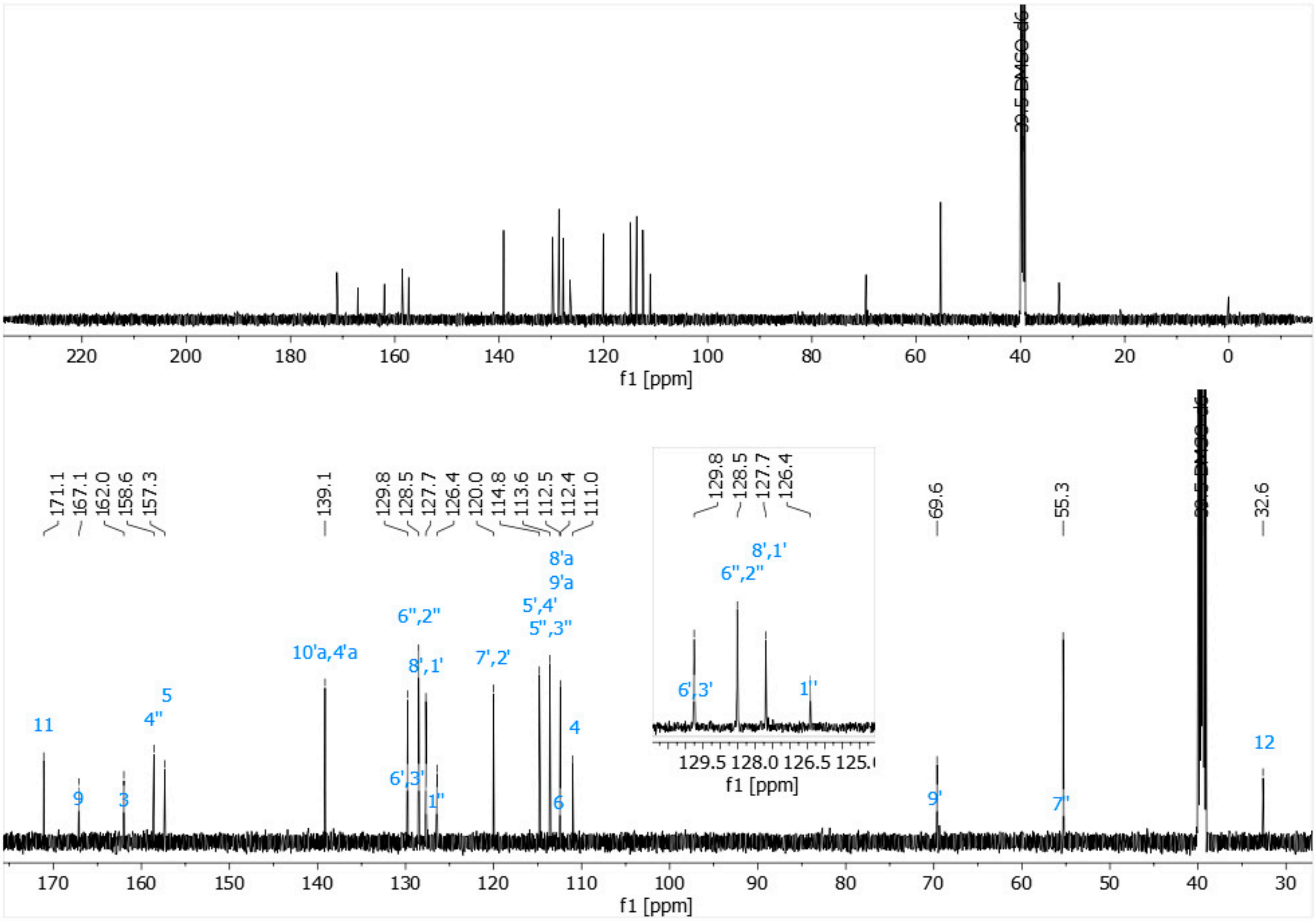

^{13}C NMR (150 MHz, DMSO-d_6) spectrum of derivative **7b**.

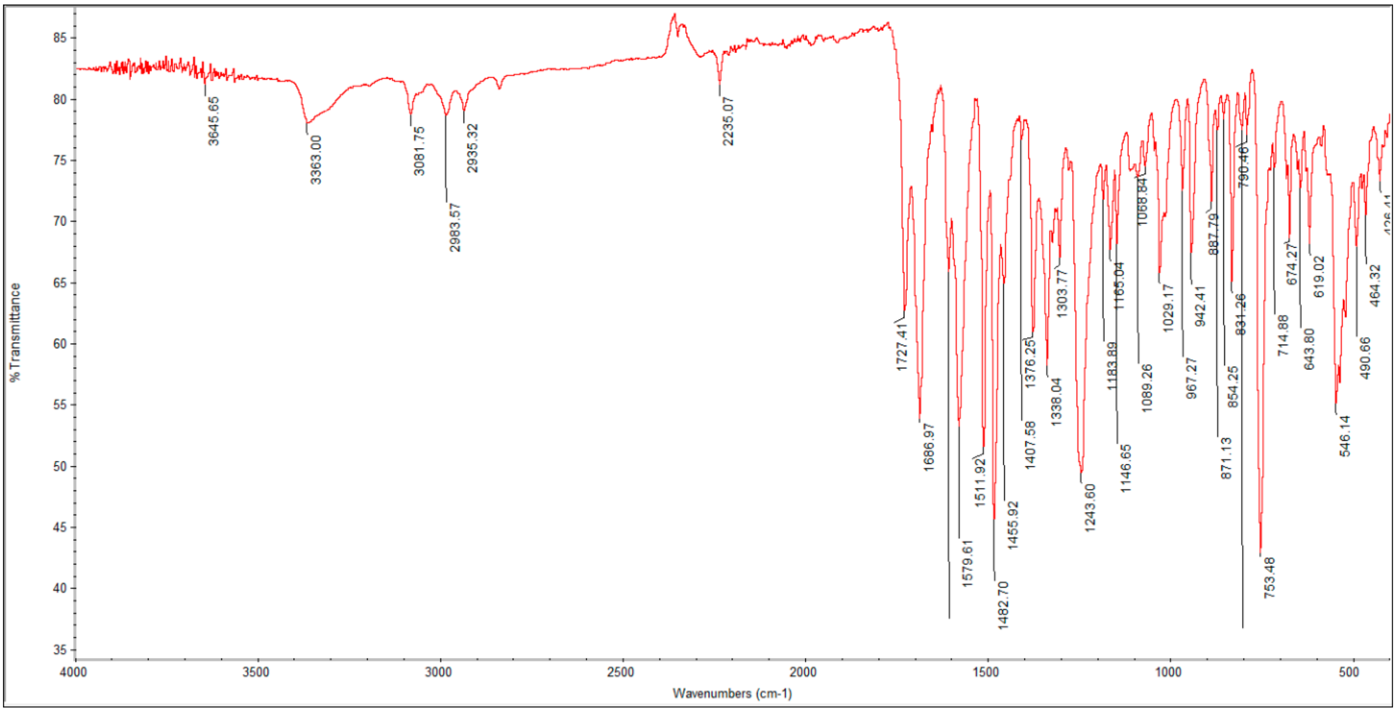

FTIR spectrum of derivative **7b**.

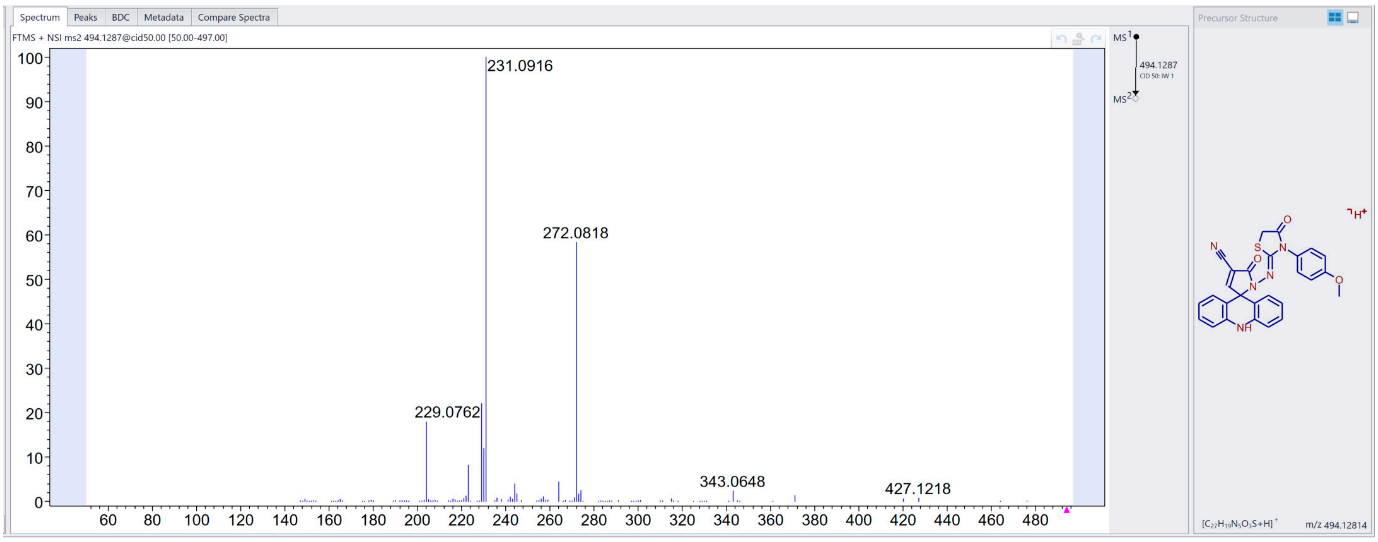

MS2 spectrum of derivative **7b**.

2.8 1'-{[(2*Z*)-3-(3-Methoxyphenyl)-4-oxo-1,3-thiazolidin-2-ylidene]amino}-5'-oxo-1',5'-dihydro-10*H*-spiro[acridine-9,2'-pyrrole]-4'-carbonitrile (**7c**)

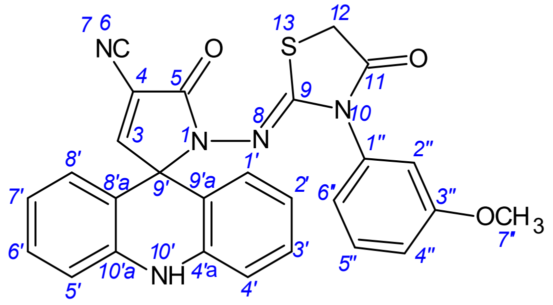

7c

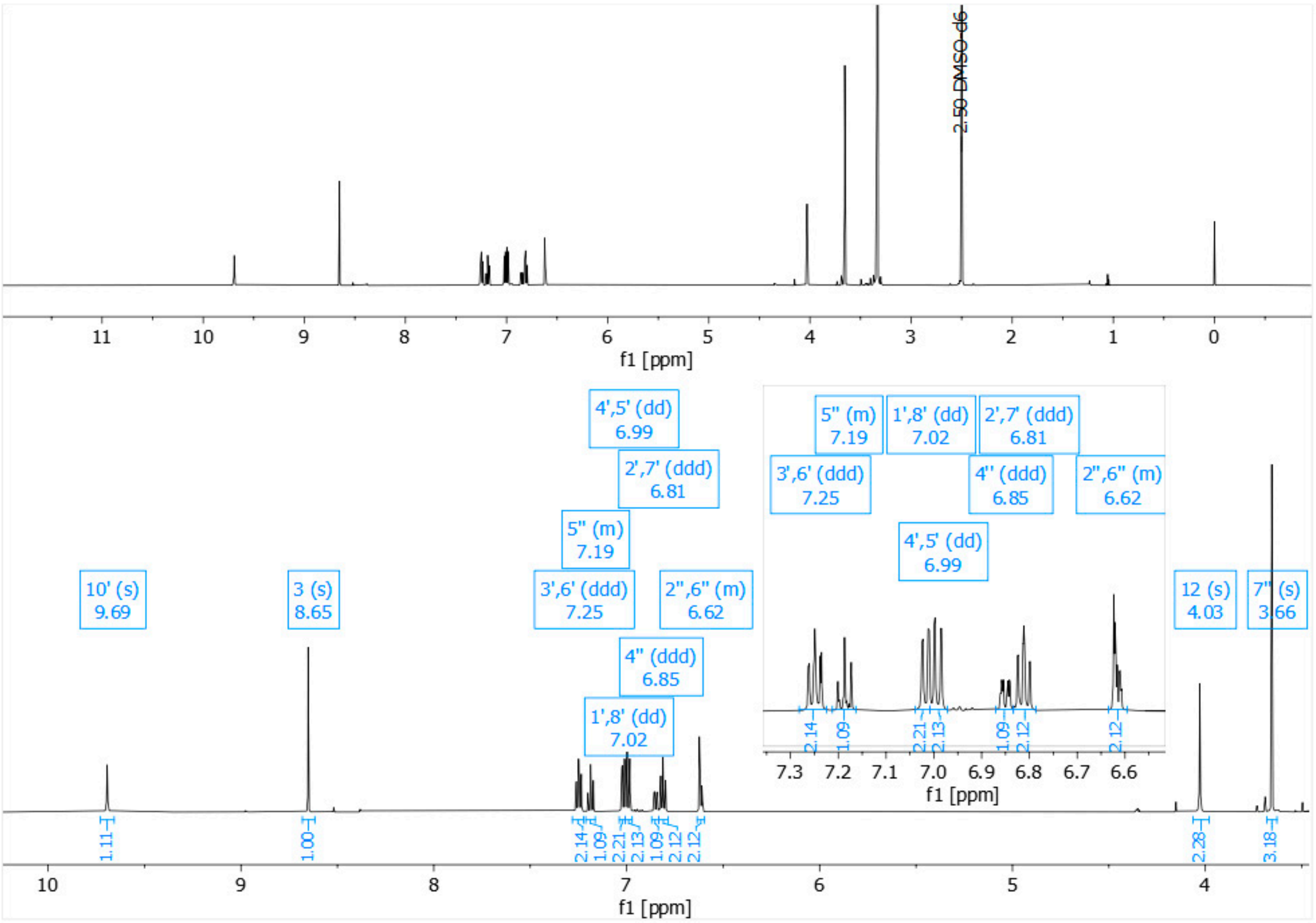

^{1}H NMR (600 MHz, DMSO-d$_6$) spectrum of derivative **7c**.

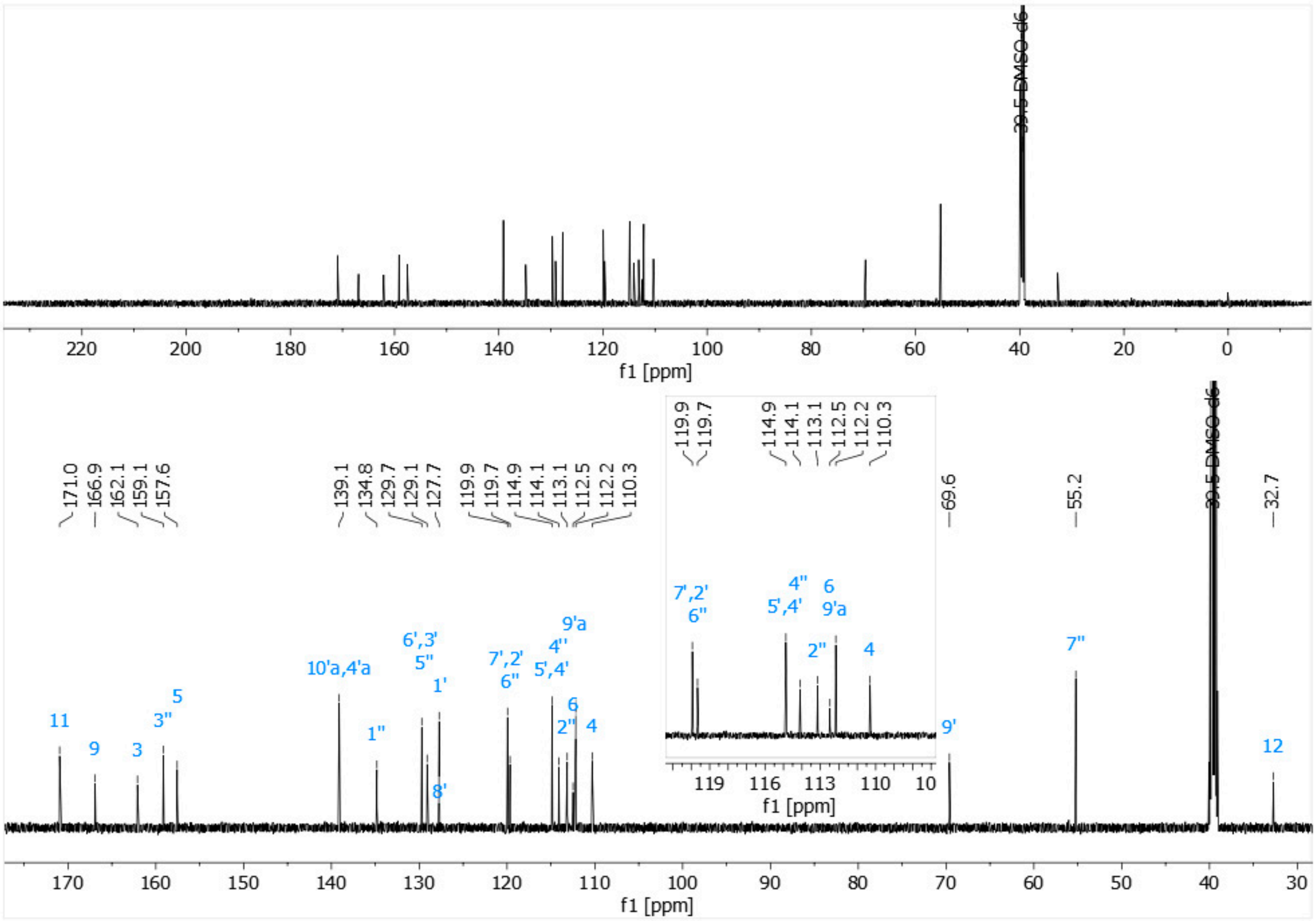

^{13}C NMR (150 MHz, DMSO-d_6) spectrum of derivative **7c**.

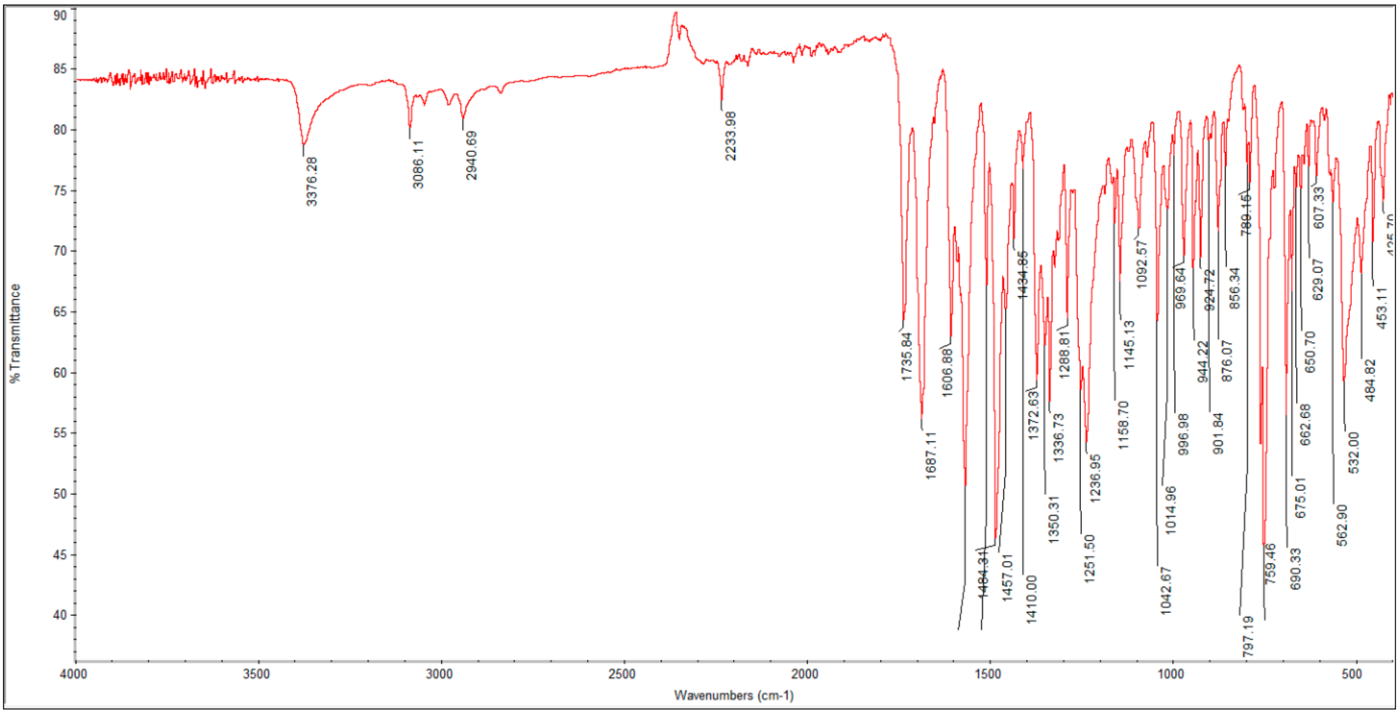

FTIR spectrum of derivative **7c**.

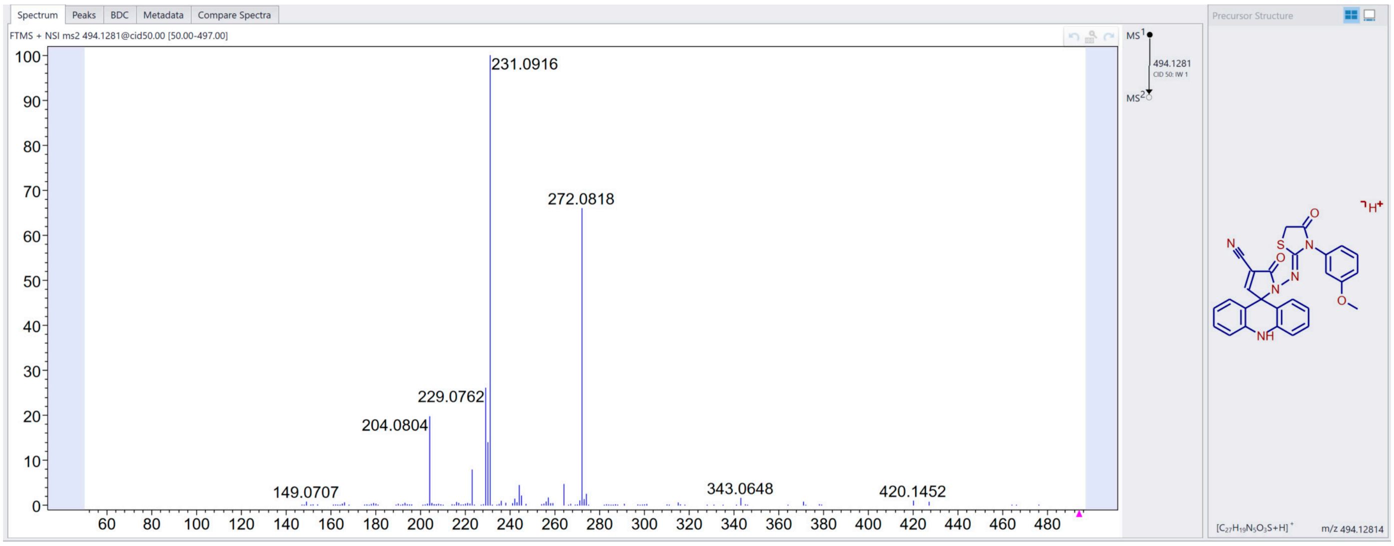

MS2 spectrum of derivative **7c**.

2.9 1'-{[(2*Z*)-3-(4-Fluorophenyl)-4-oxo-1,3-thiazolidin-2-ylidene]amino}-5'-oxo-1',5'-dihydro-10*H*-spiro[acridine-9,2'-pyrrole]-4'-carbonitrile (**7d**)

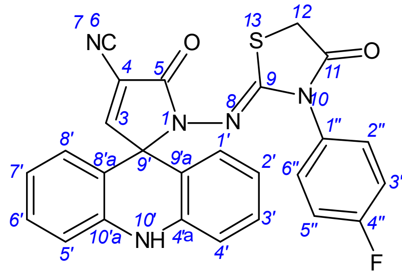

7d

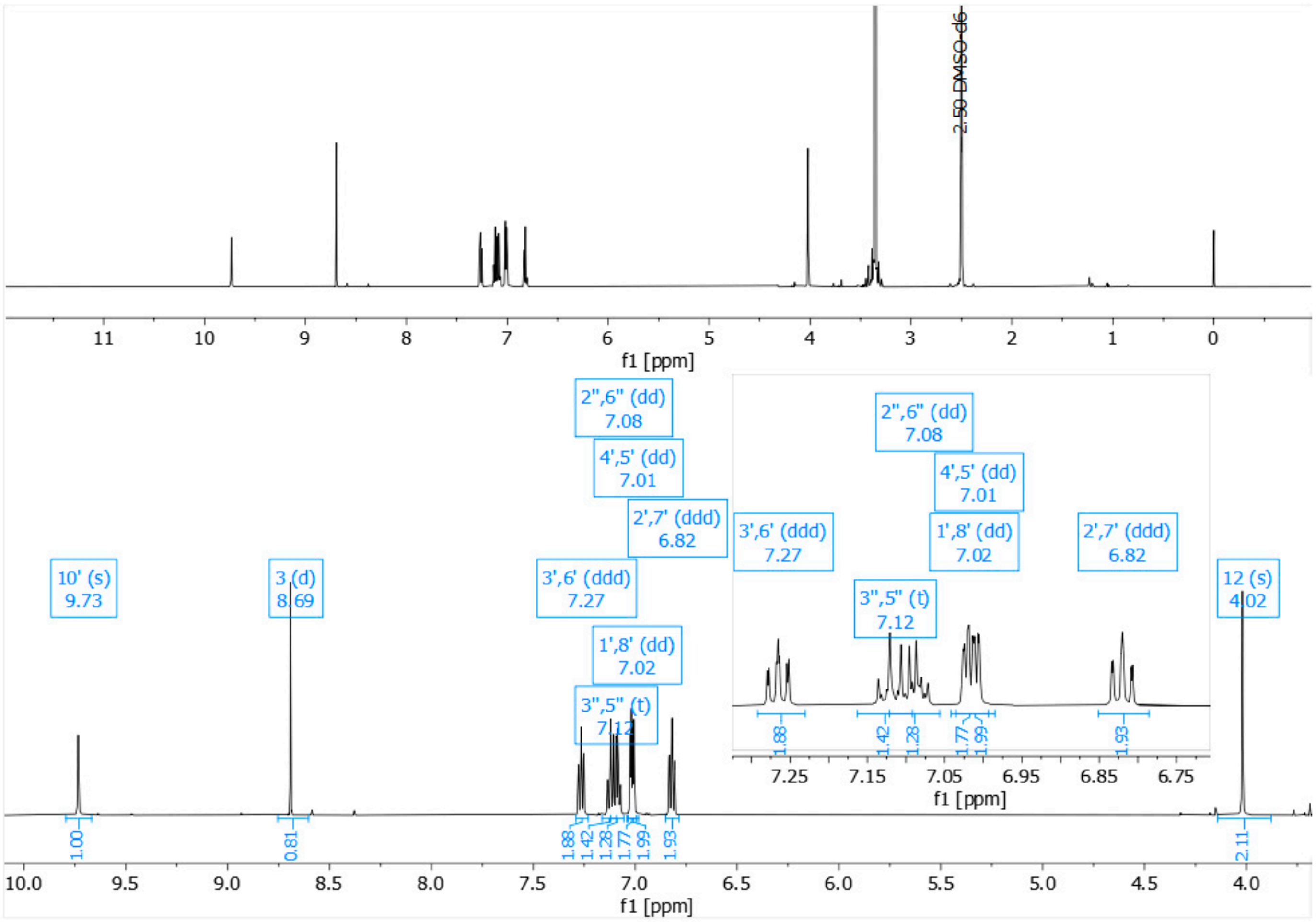

^{1}H NMR (600 MHz, DMSO-d$_6$) spectrum of derivative **7d**.

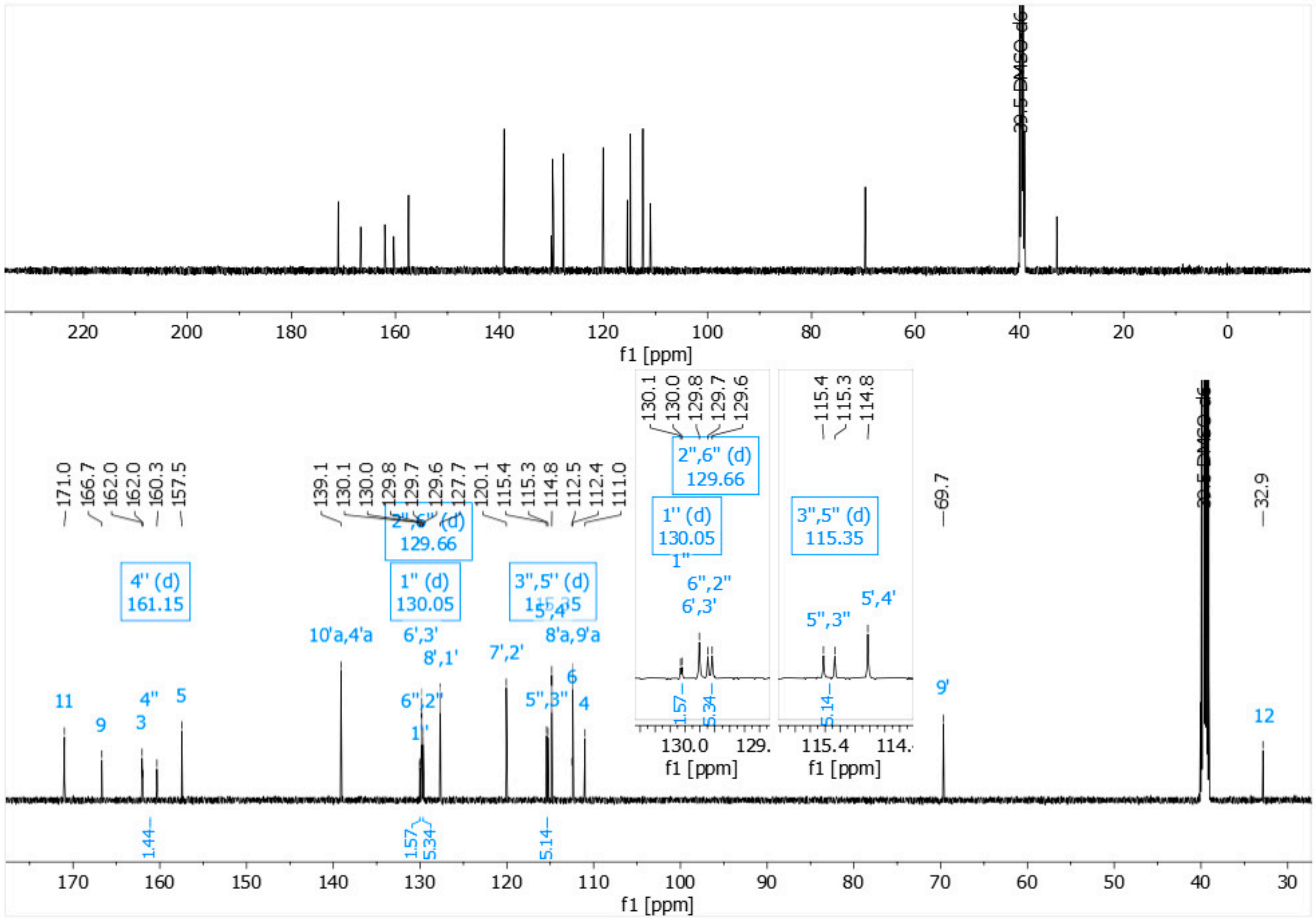

^{13}C NMR (150 MHz, DMSO-d$_6$) spectrum of derivative **7d**.

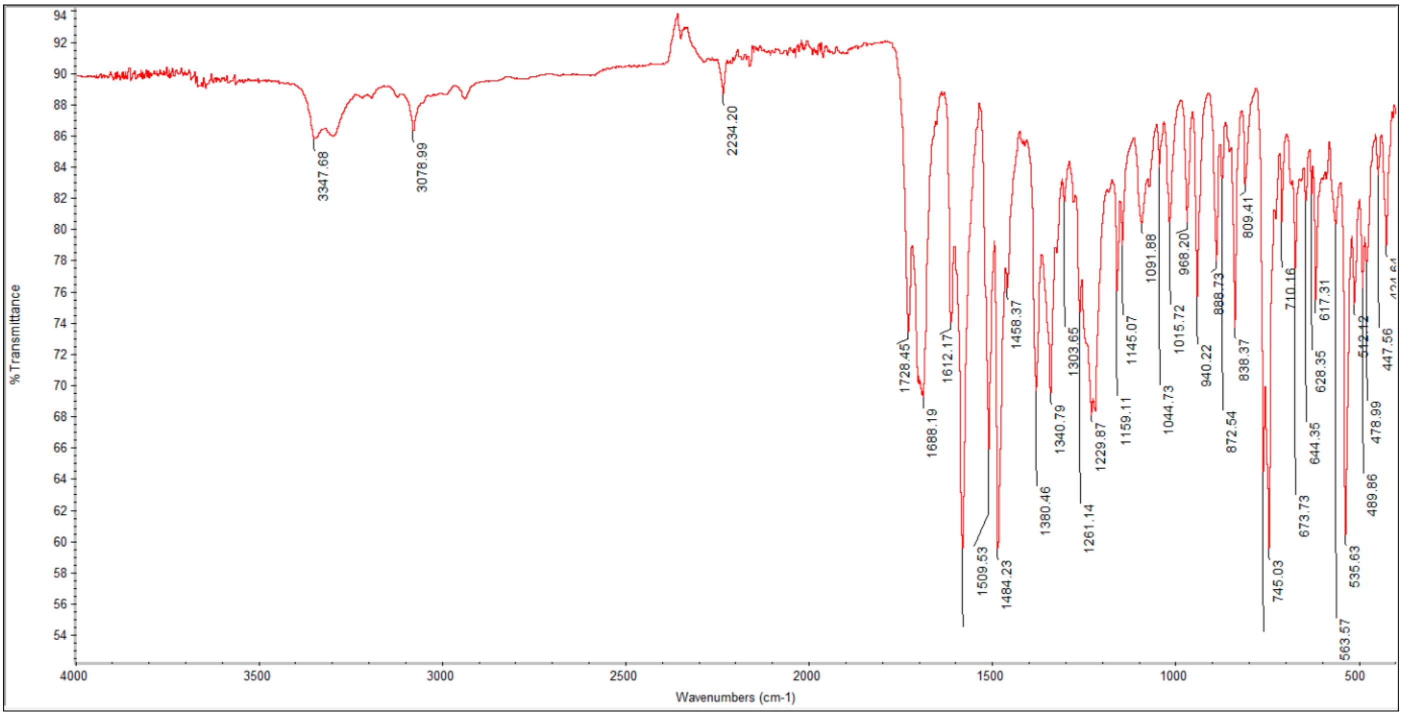

FTIR spectrum of derivative **7d**.

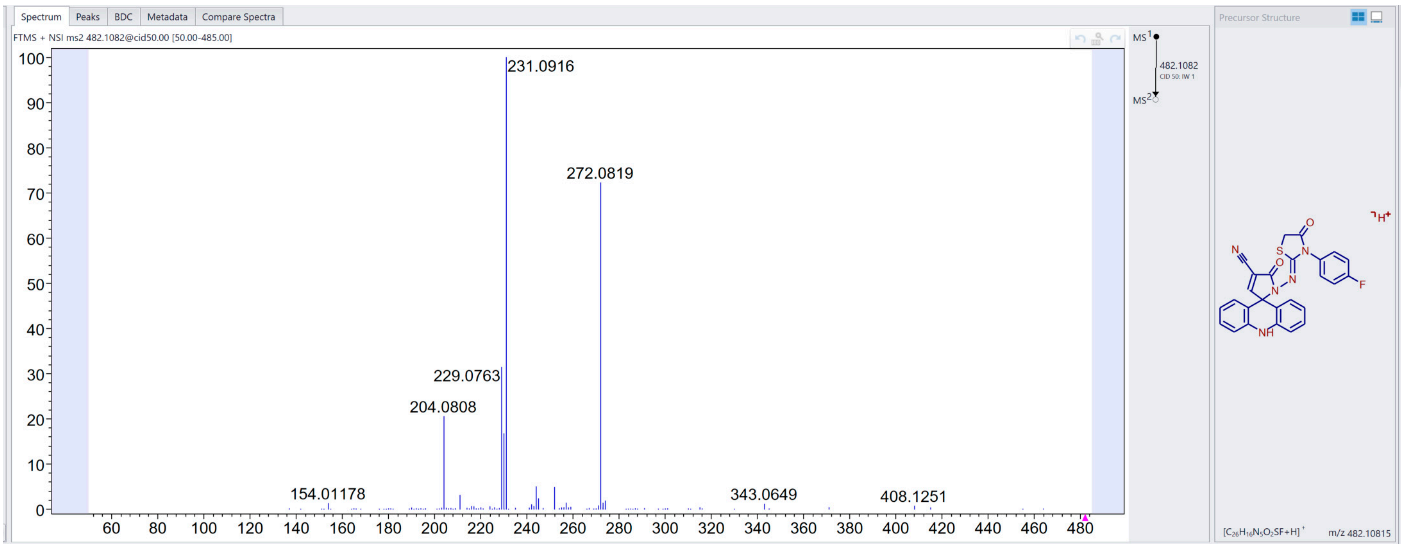

MS2 spectrum of derivative **7d**.

2.10 1'-{[(2*Z*)-3-(4-Nitrophenyl)-4-oxo-1,3-thiazolidin-2-ylidene]amino}-5'-oxo-1',5'-dihydro-10*H*-spiro[acridine-9,2'-pyrrole]-4'-carbonitrile (**7e**)

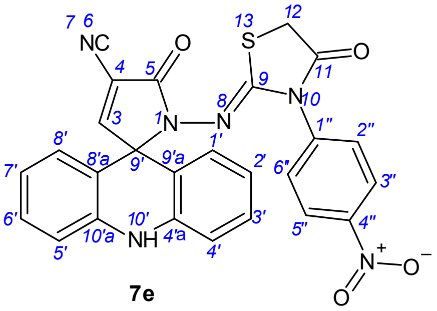

7e

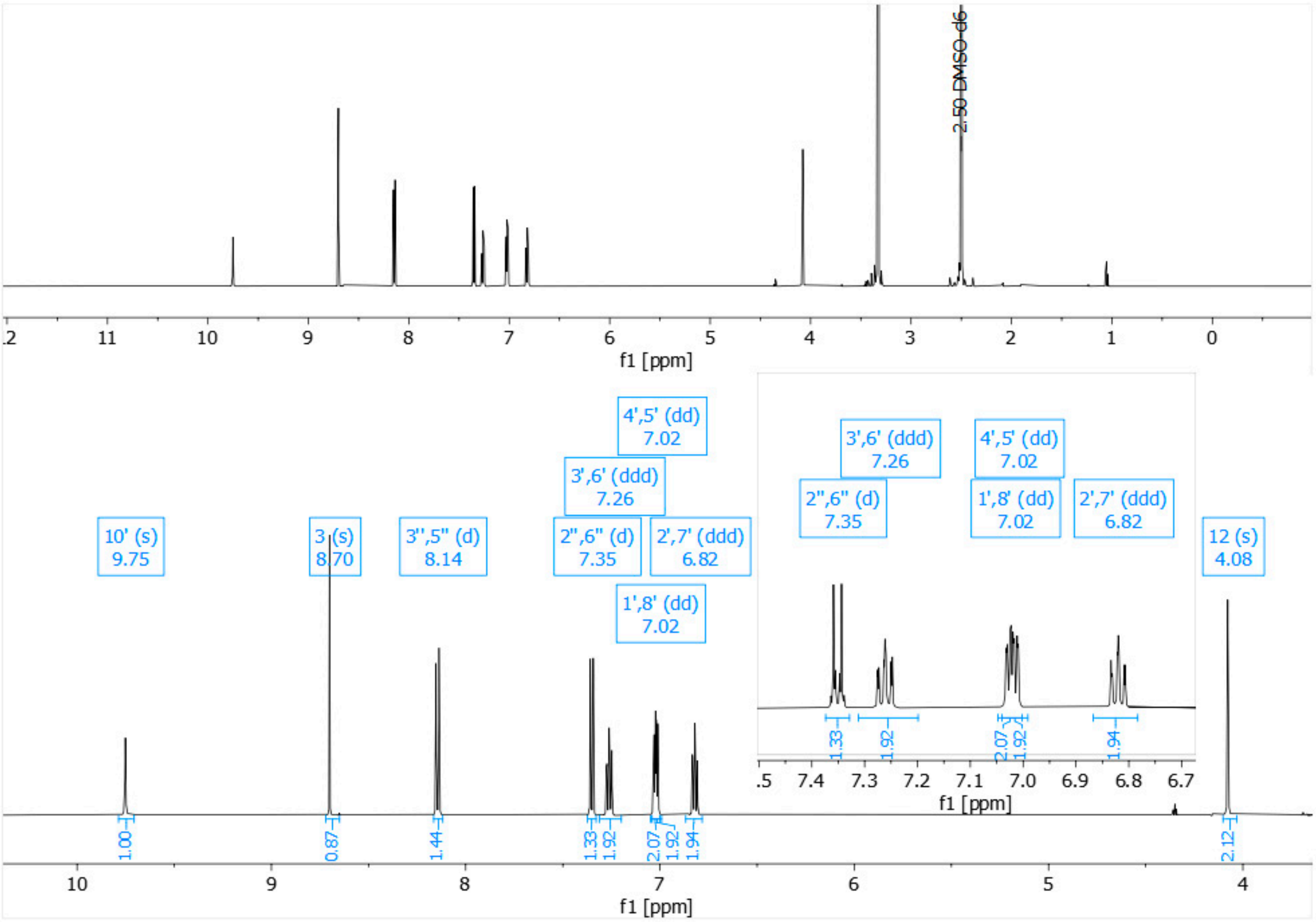

^{1}H NMR (600 MHz, DMSO-d$_6$) spectrum of derivative **7e**.

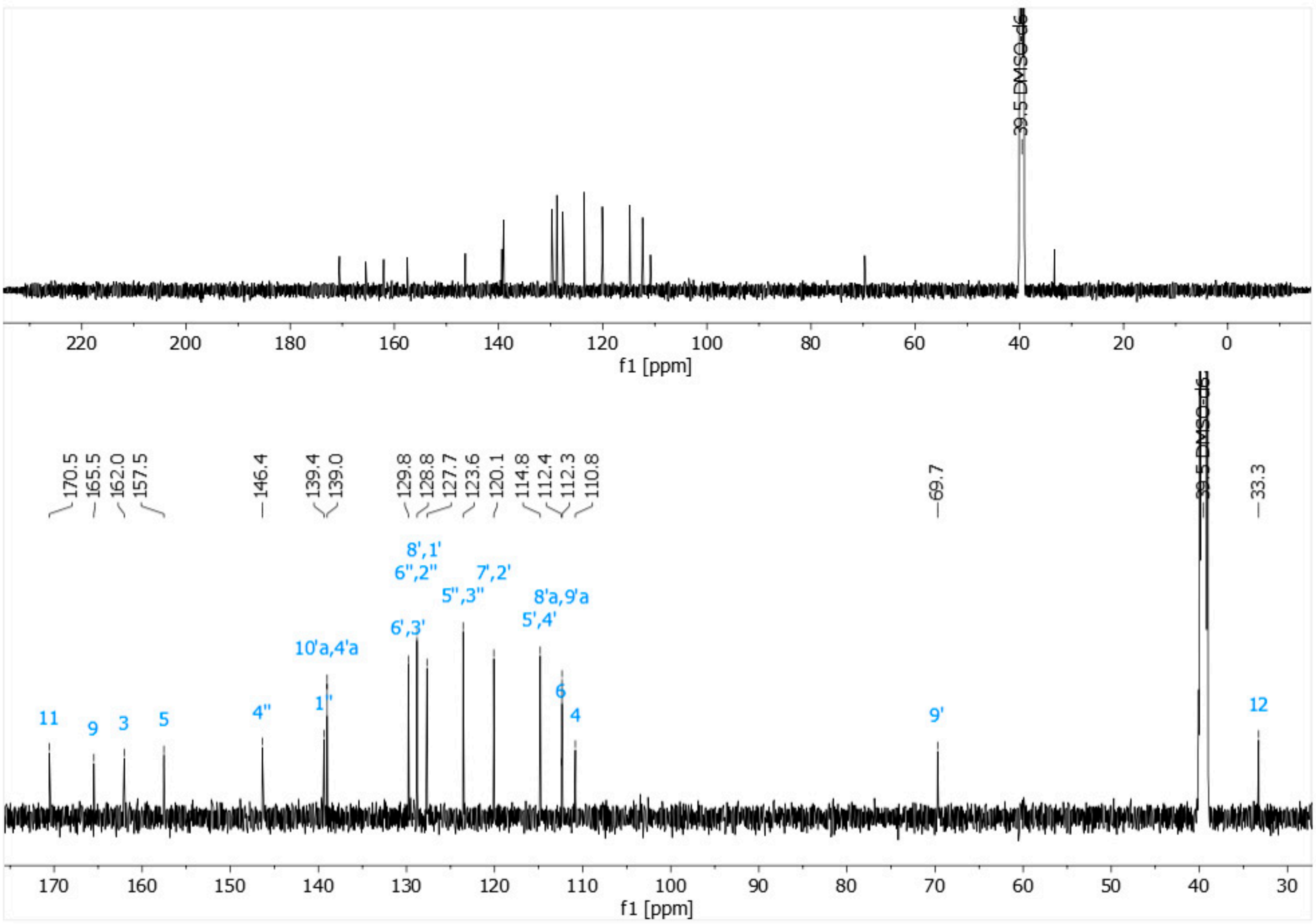

^{13}C NMR (150 MHz, DMSO-d$_6$) spectrum of derivative **7e**.

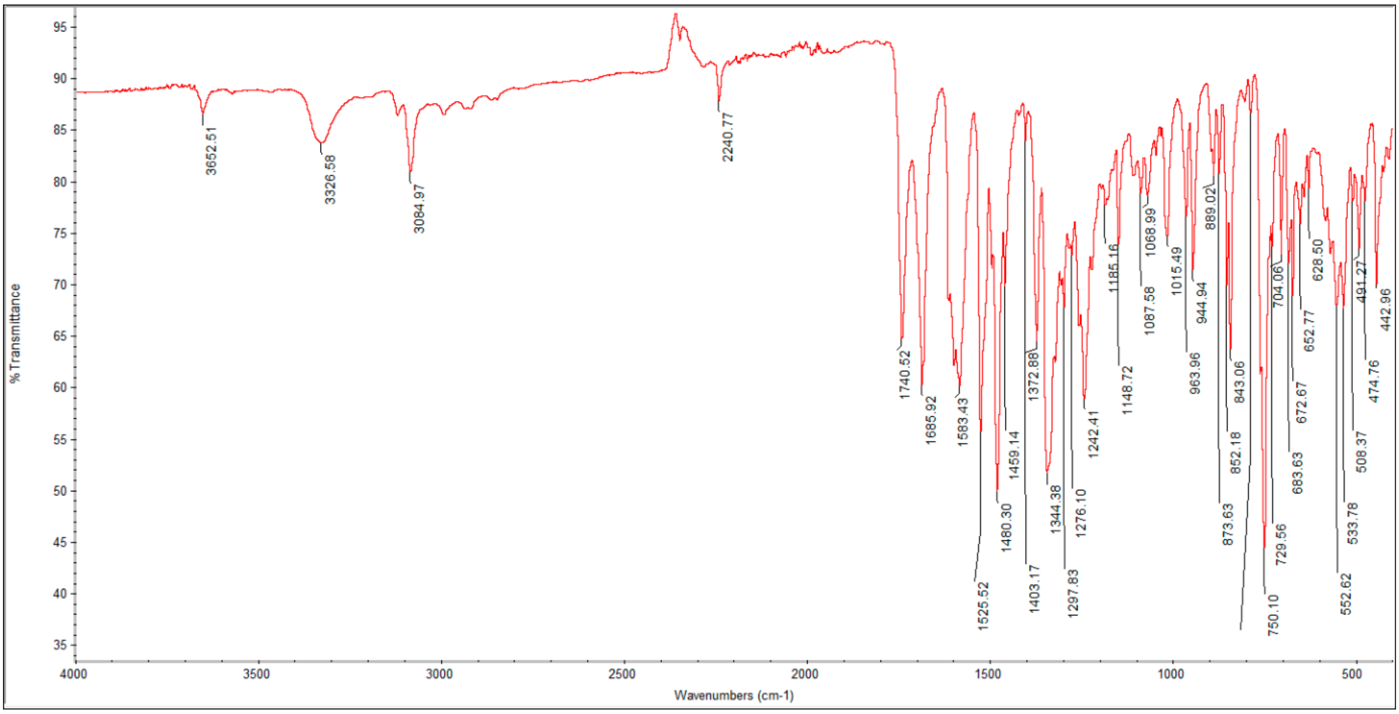

FTIR spectrum of derivative **7e**.

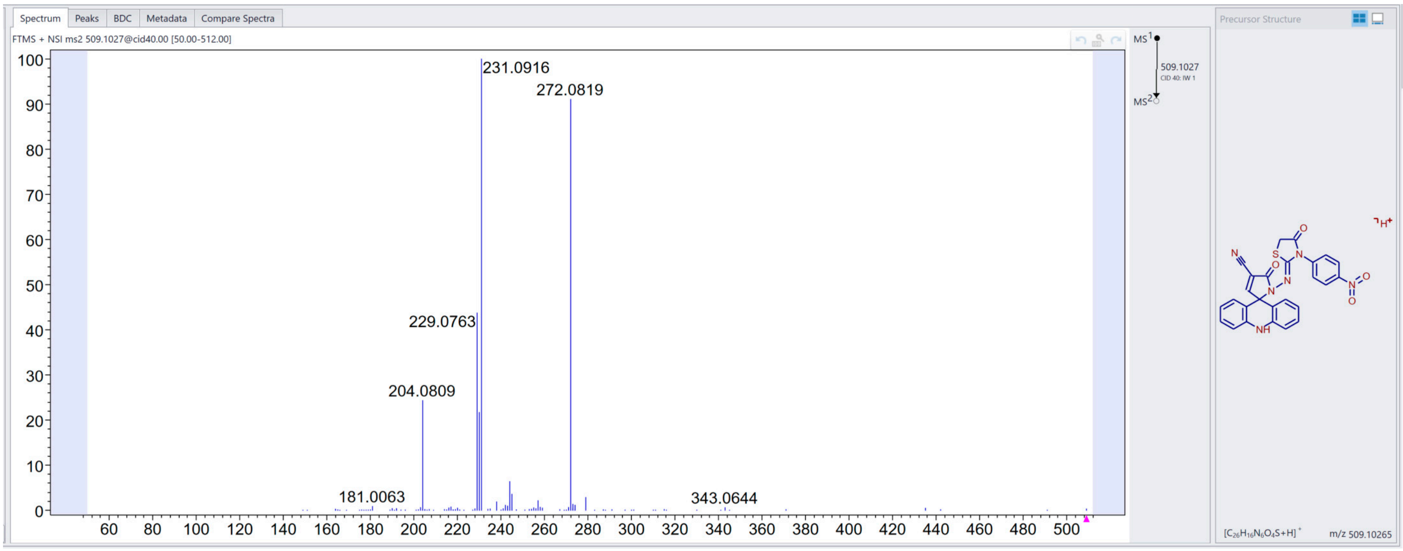

MS2 spectrum of derivative **7e**.

2.11 Ethyl 2-[(2*Z*,5*Z*)-2-({4'-cyano-5'-oxo-1',5'-dihydro-10*H*-spiro[acridine-9,2'-pyrrol]-1'-yl}imino)-4-oxo-3-phenyl-1,3-thiazolidin-5-ylidene]acetate (8a)

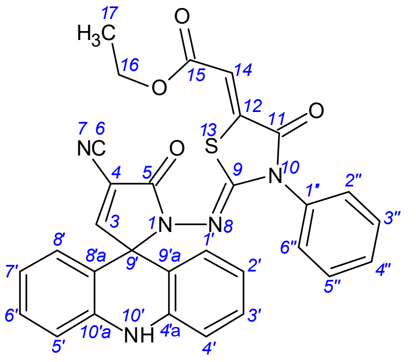

8a

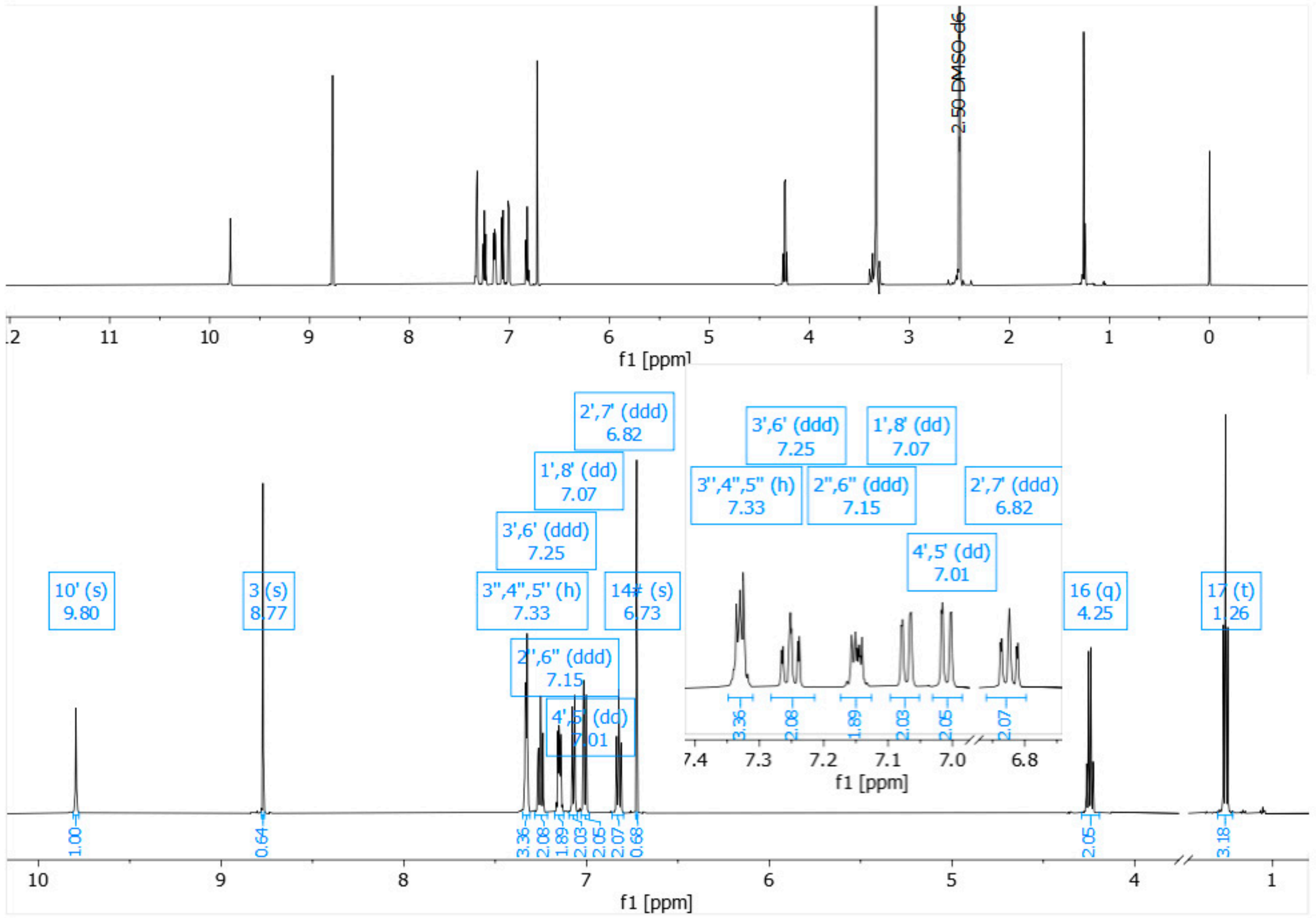

^{1}H NMR (600 MHz, DMSO-d_6) spectrum of derivative **8a**.

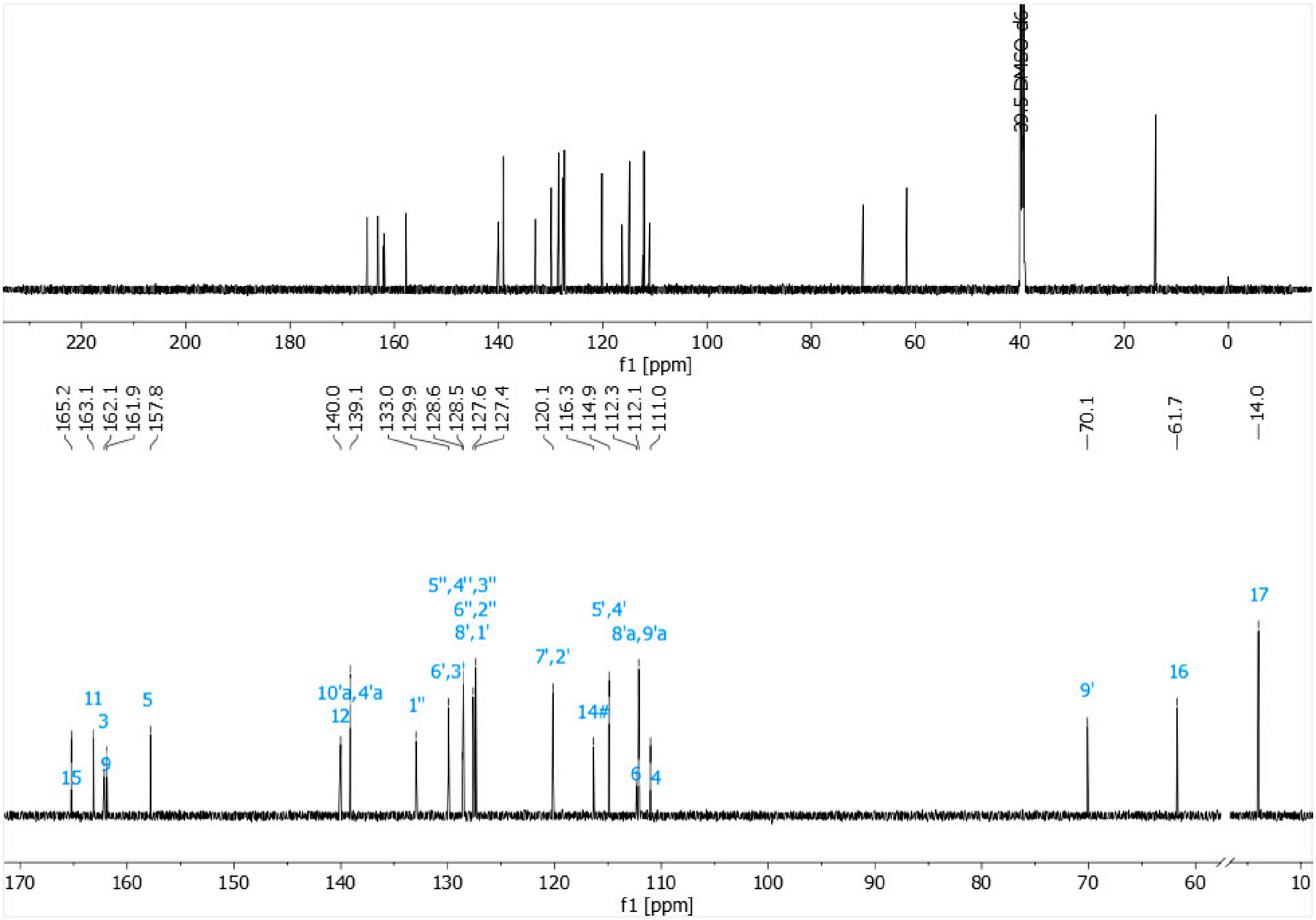

^{13}C NMR (150 MHz, DMSO-d$_6$) spectrum of derivative **8a**.

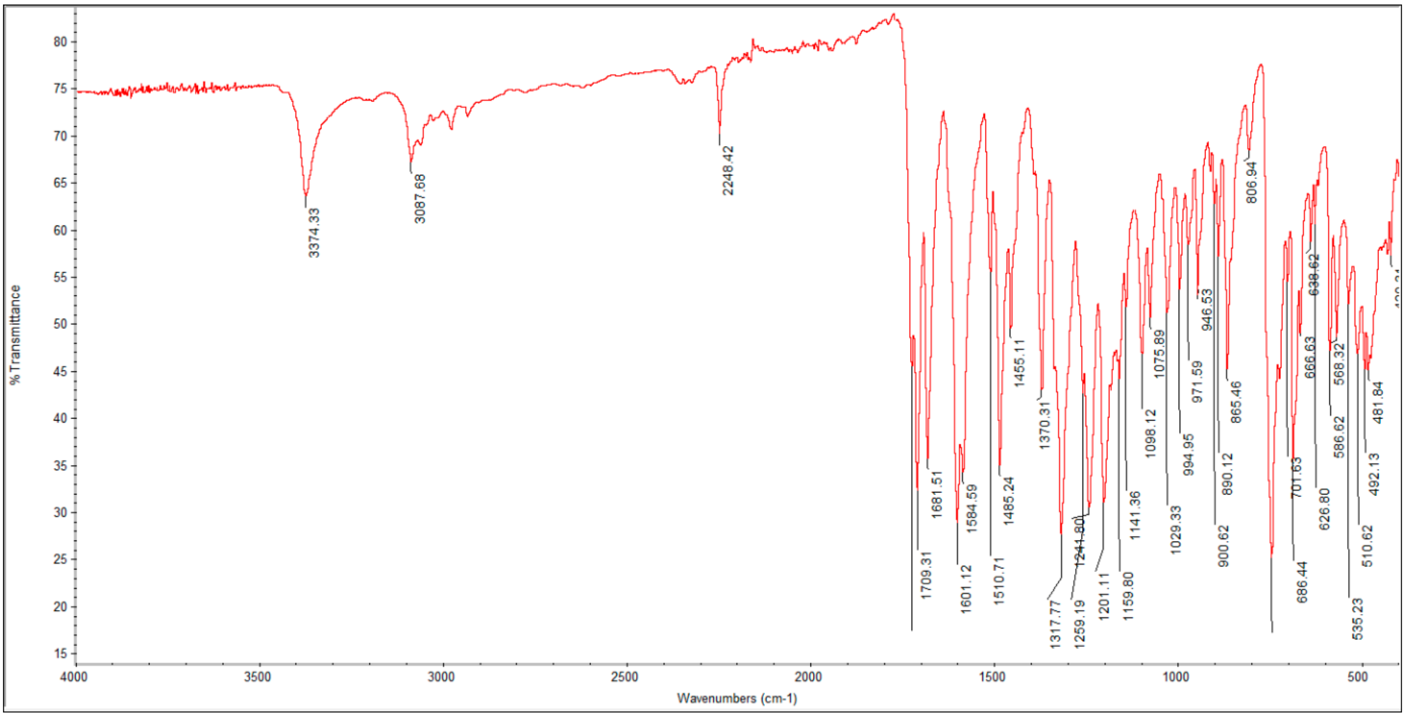

FTIR spectrum of derivative **8a**.

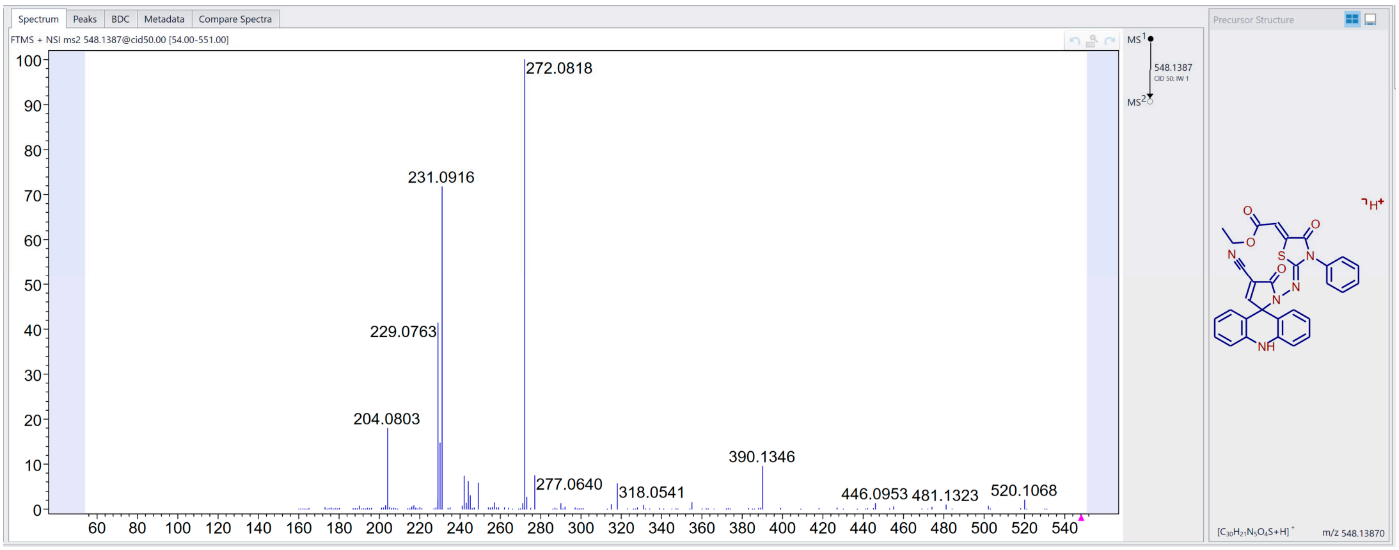

MS2 spectrum of derivative **8a**.

2.12 Ethyl 2-[(2*Z*,5*Z*)-2-({4'-cyano-5'-oxo-1',5'-dihydro-10*H*-spiro[acridine-9,2'-pyrrol]-1'-yl}imino)-3-(4-methoxyphenyl)-4-oxo-1,3-thiazolidin-5-ylidene]acetate (**8b**)

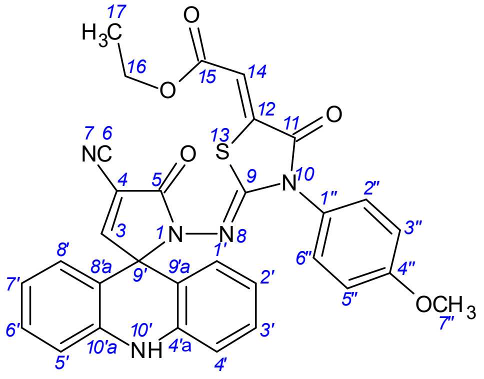

8b

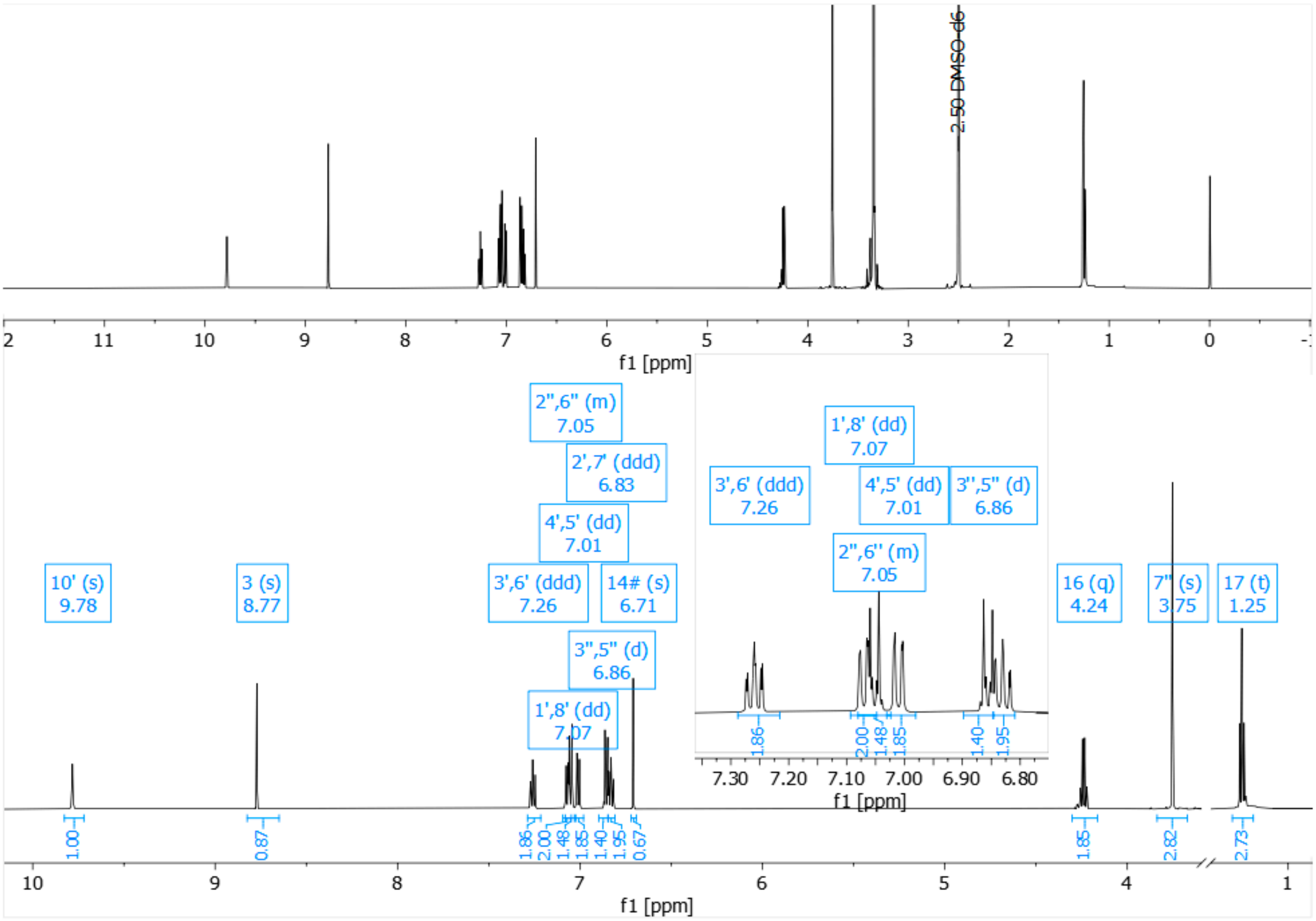

^{1}H NMR (600 MHz, DMSO-d$_6$) spectrum of derivative **8b**.

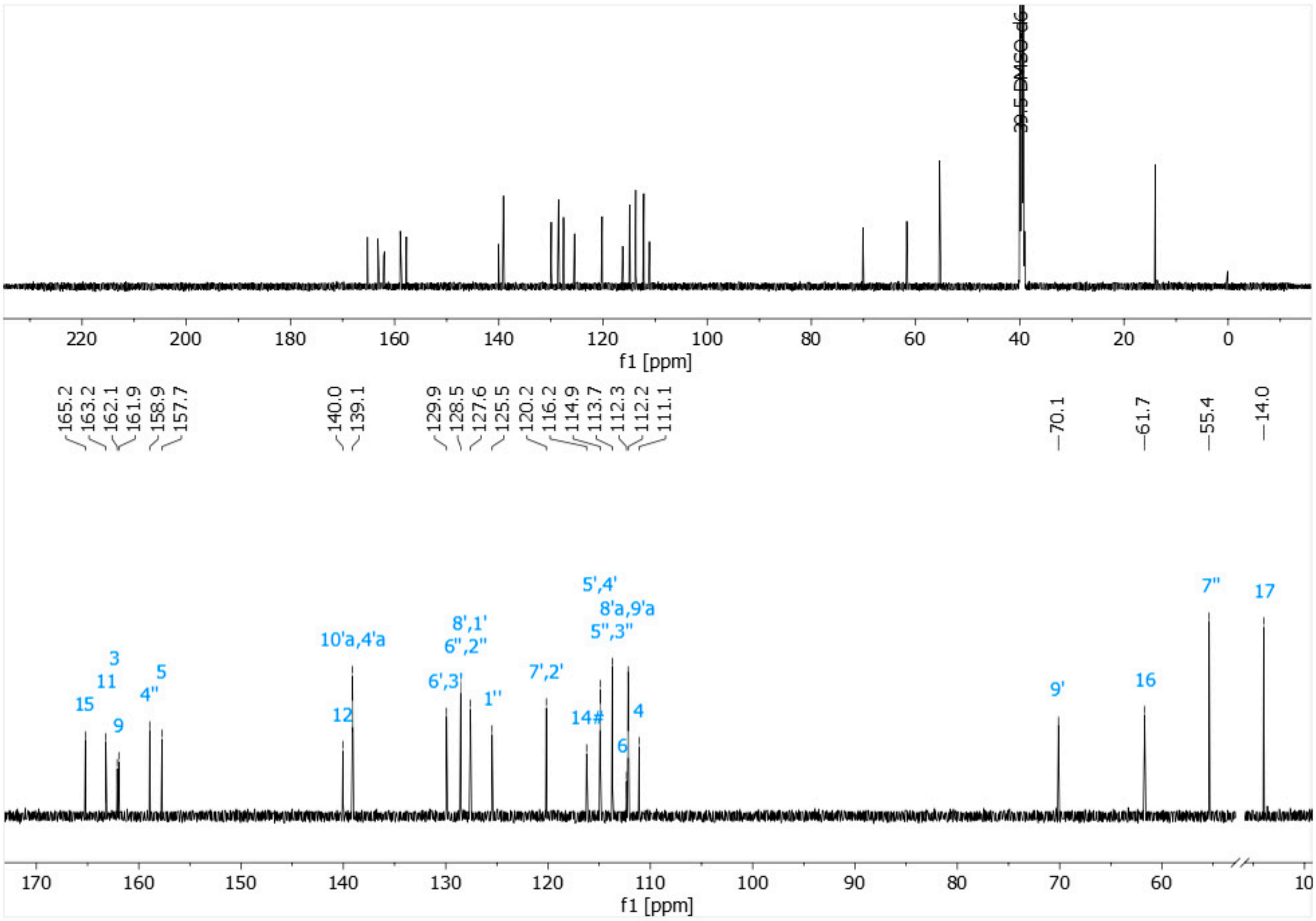

^{13}C NMR (150 MHz, DMSO-d$_6$) spectrum of derivative **8b**.

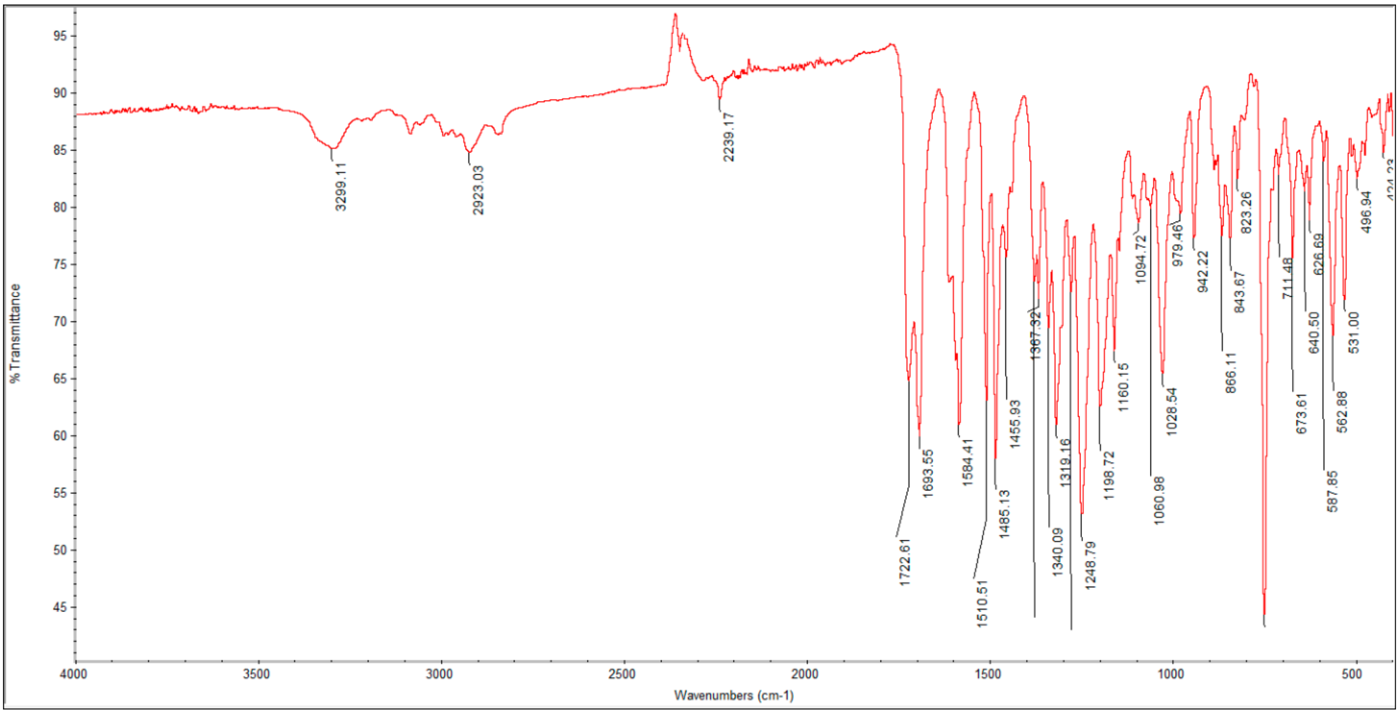

FTIR spectrum of derivative **8b**.

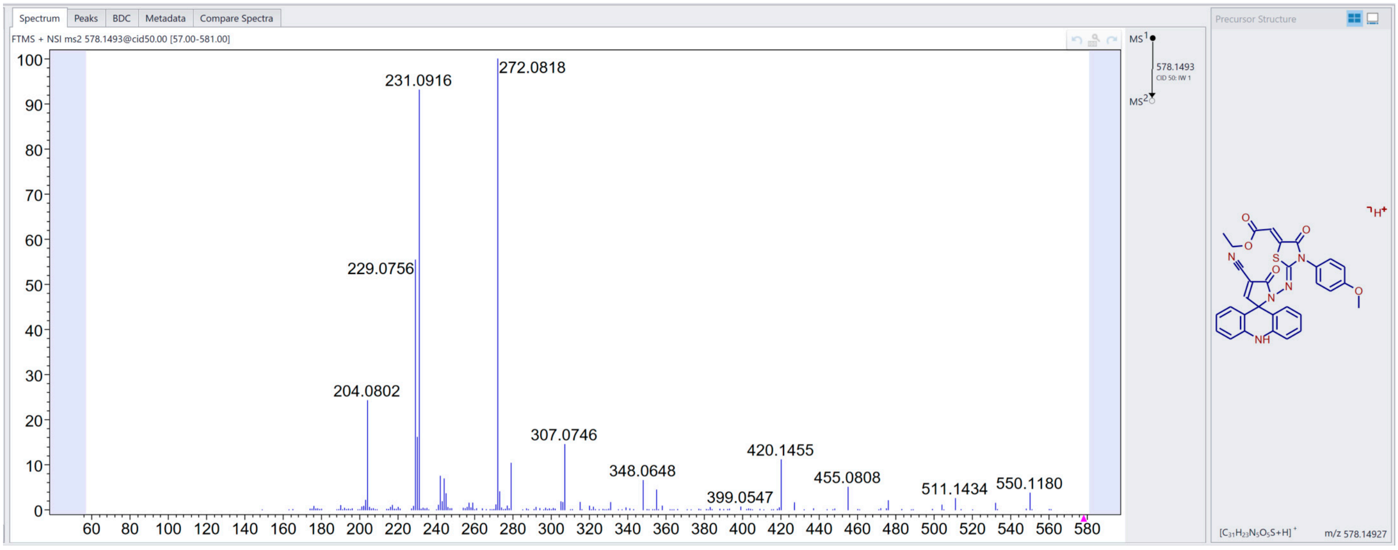

MS2 spectrum of derivative **8b**.

2.13 Ethyl 2-[(2*Z*,5*Z*)-2-({4'-cyano-5'-oxo-1',5'-dihydro-10*H*-spiro[acridine-9,2'-pyrrol]-1'-yl}imino)-3-(3-methoxyphenyl)-4-oxo-1,3-thiazolidin-5-ylidene]acetate (**8c**)

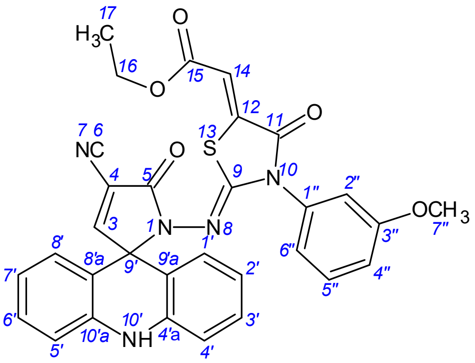

8c

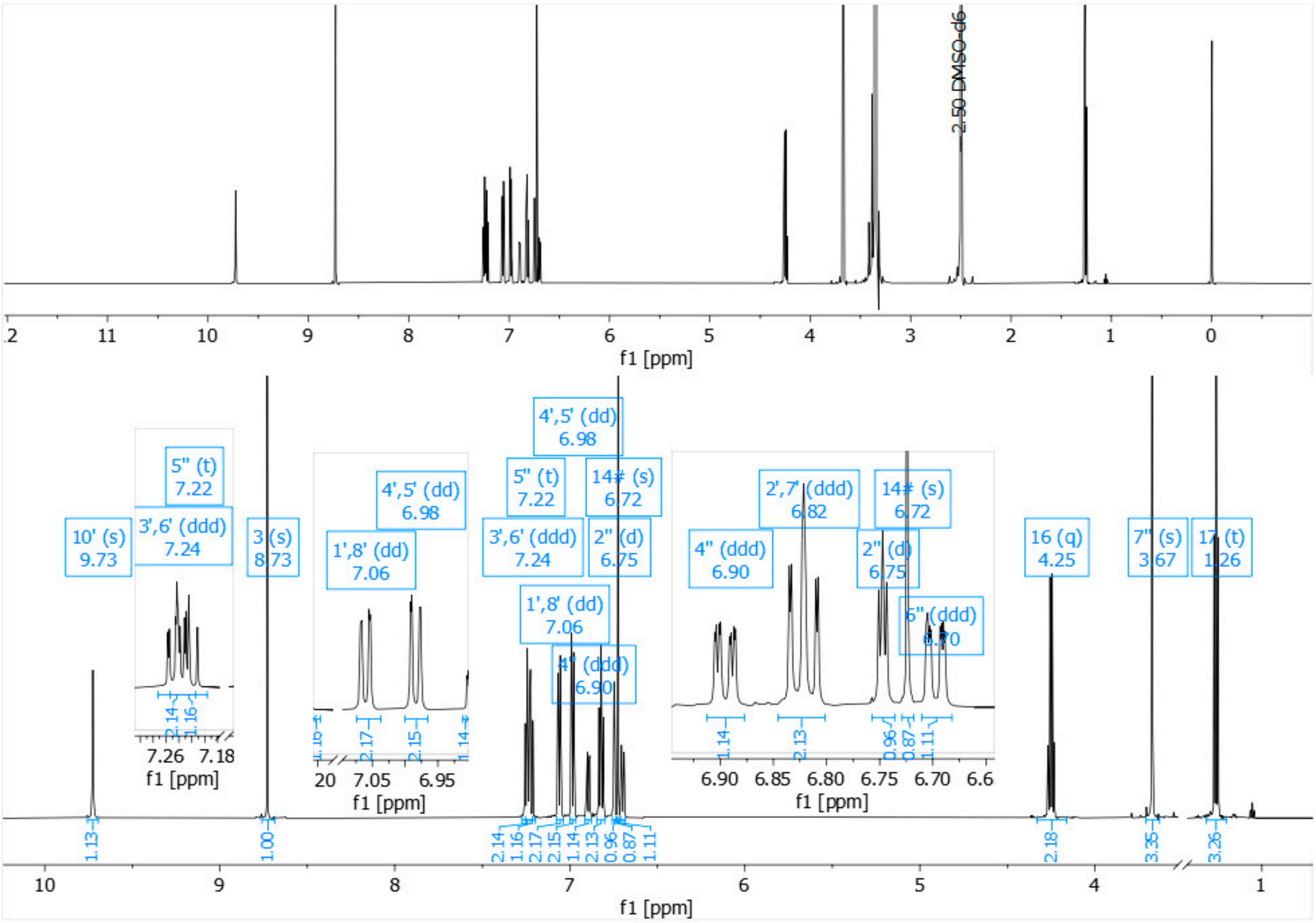

^{1}H NMR (600 MHz, DMSO-d$_6$) spectrum of derivative **8c**.

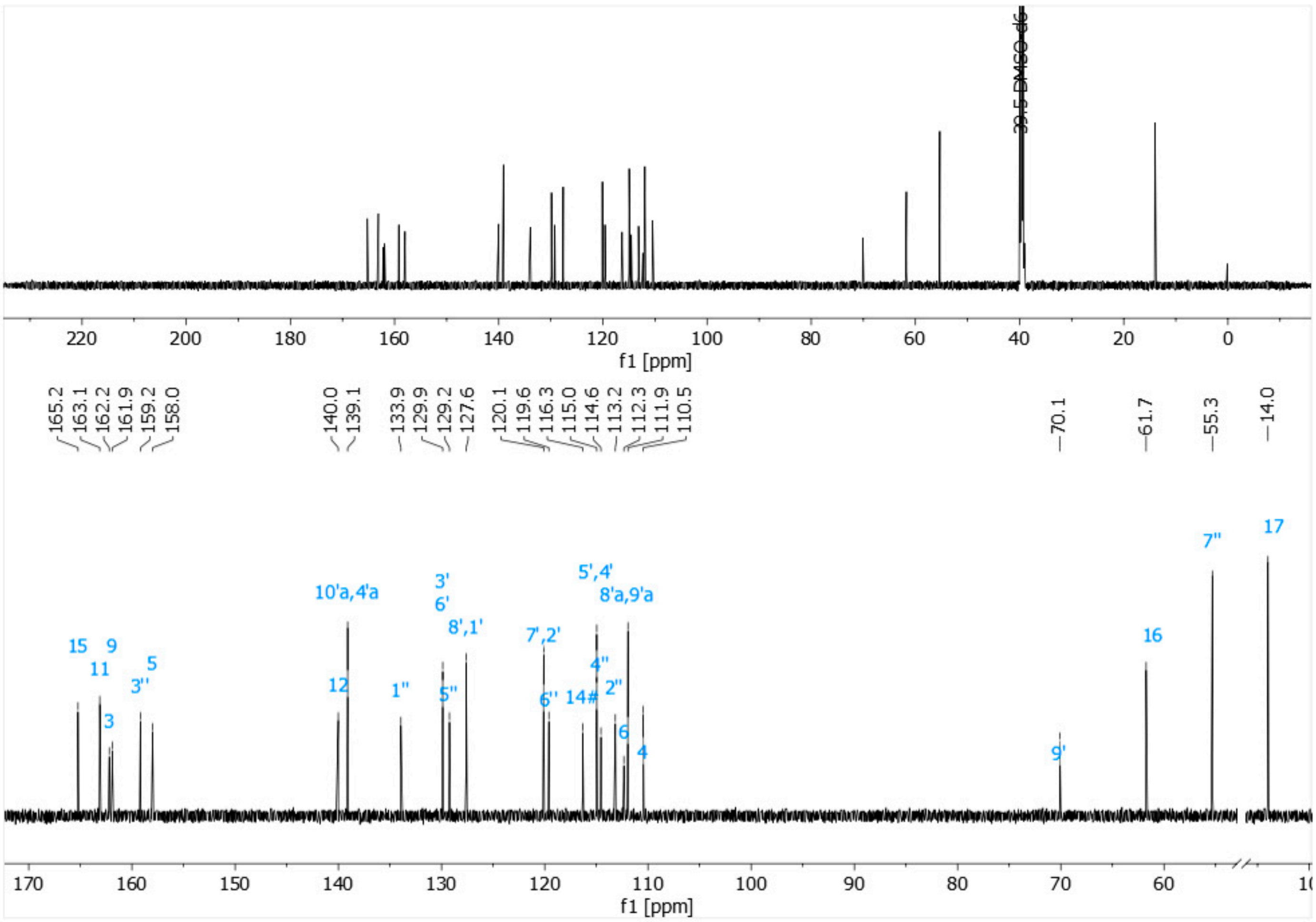

^{13}C NMR (150 MHz, DMSO-d$_6$) spectrum of derivative **8c**.

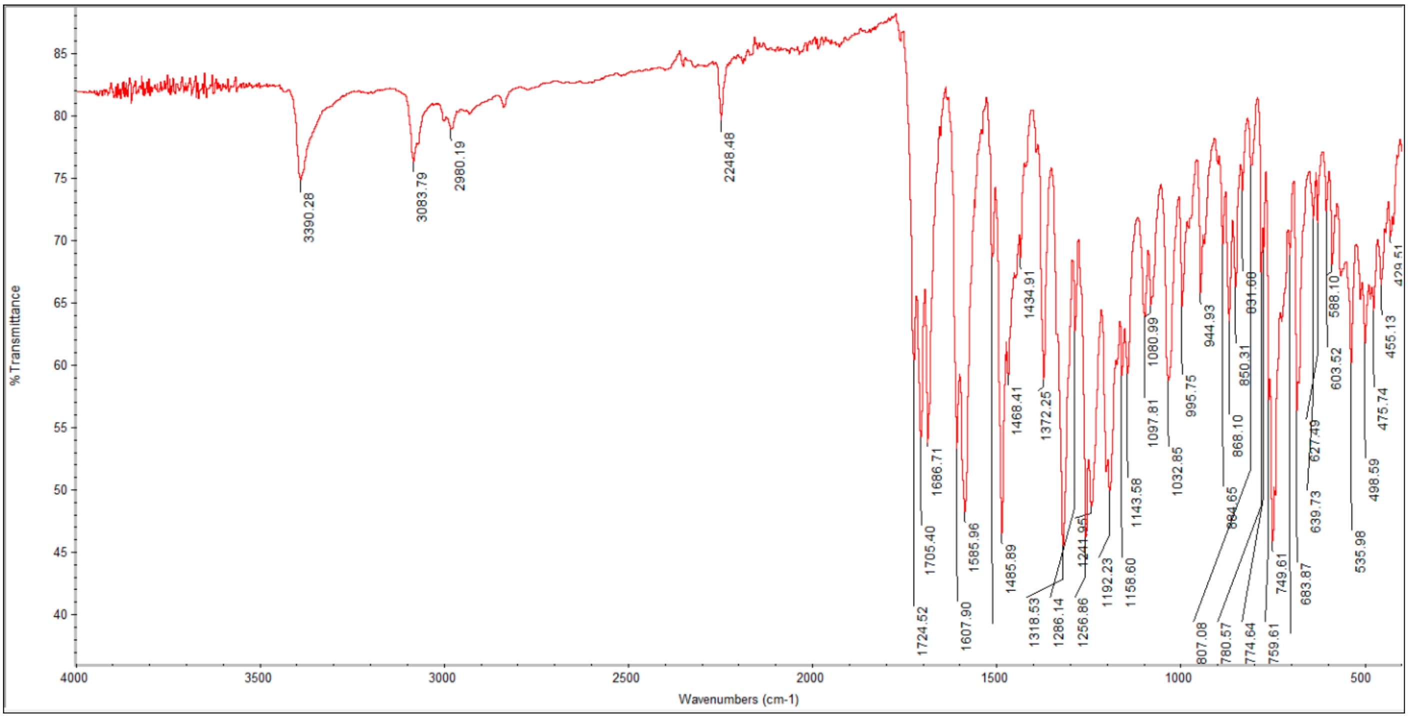

FTIR spectrum of derivative **8c**.

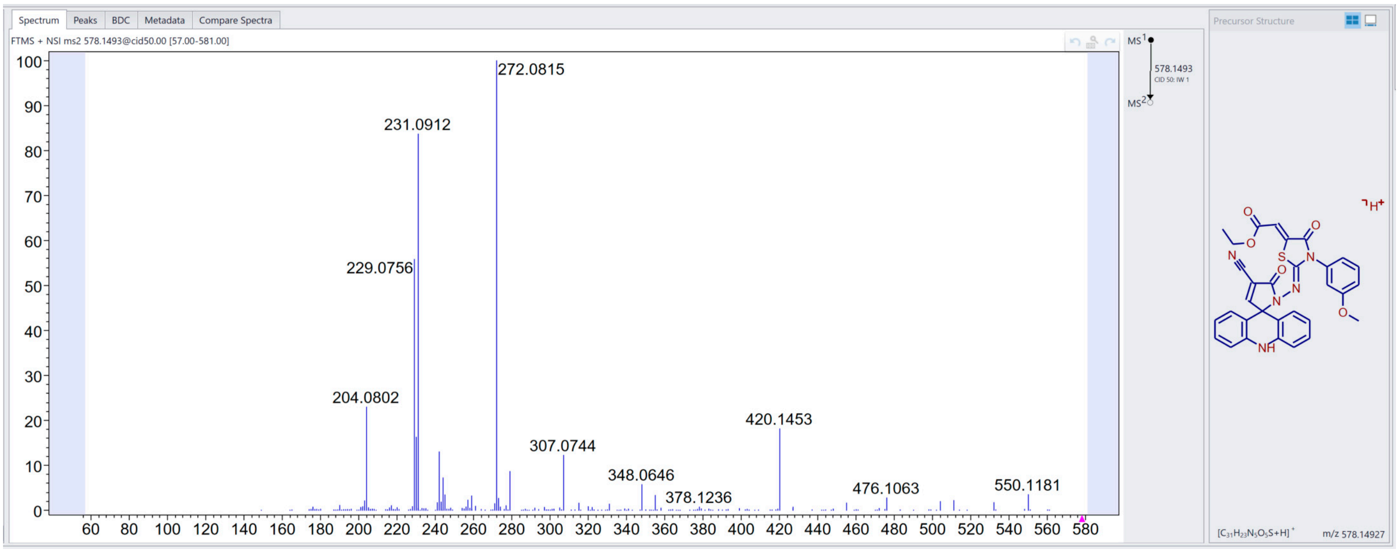

MS2 spectrum of derivative **8c**.

2.14 Ethyl 2-[(2*Z*,5*Z*)-2-({4'-cyano-5'-oxo-1',5'-dihydro-10*H*-spiro[acridine-9,2'-pyrrol]-1'-yl}imino)-3-(4-fluorophenyl)-4-oxo-1,3-thiazolidin-5-ylidene]acetate (**8d**)

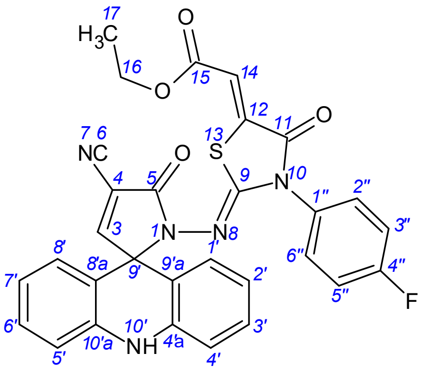

8d

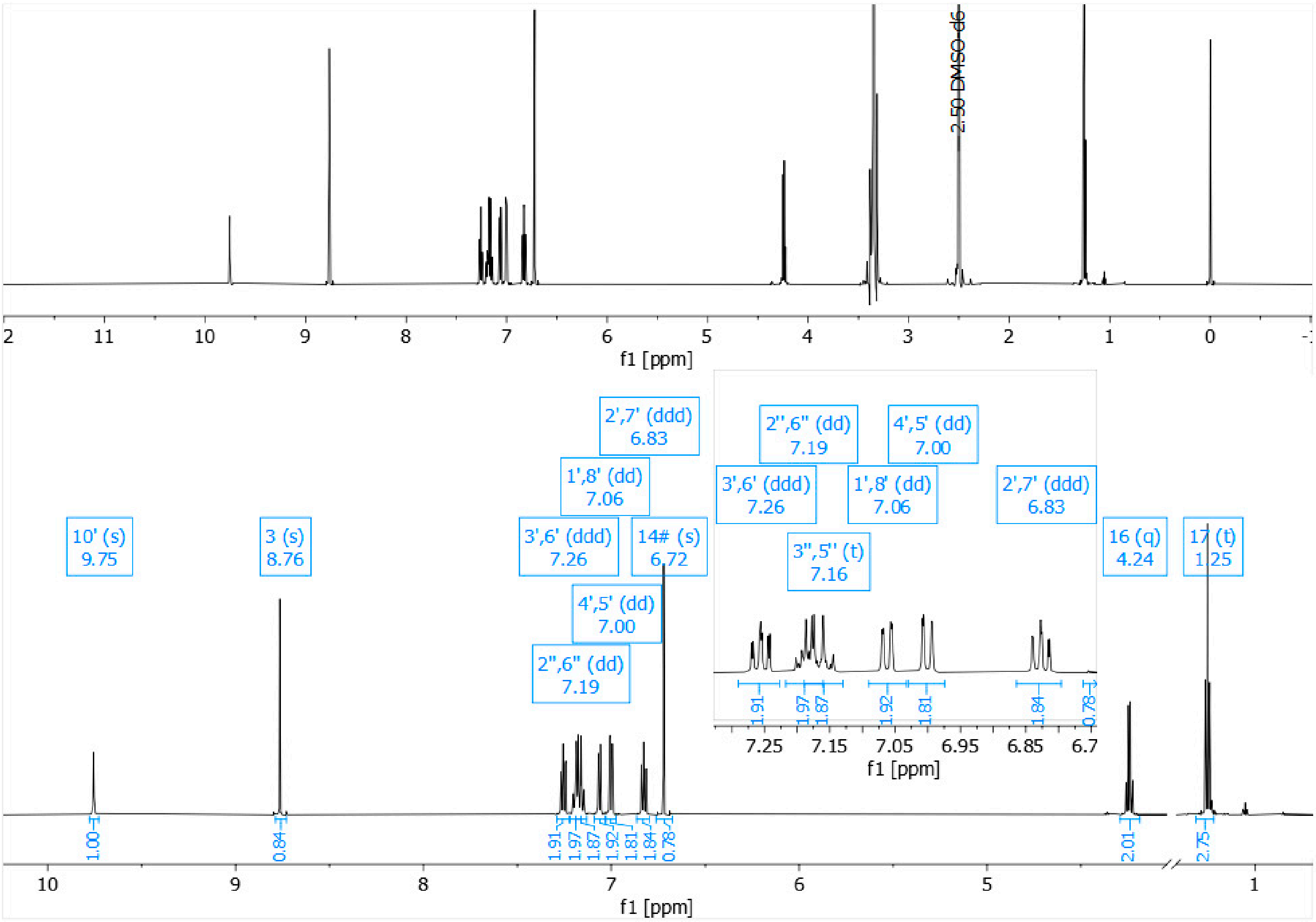

^{1}H NMR (600 MHz, DMSO-d$_6$) spectrum of derivative **8d**.

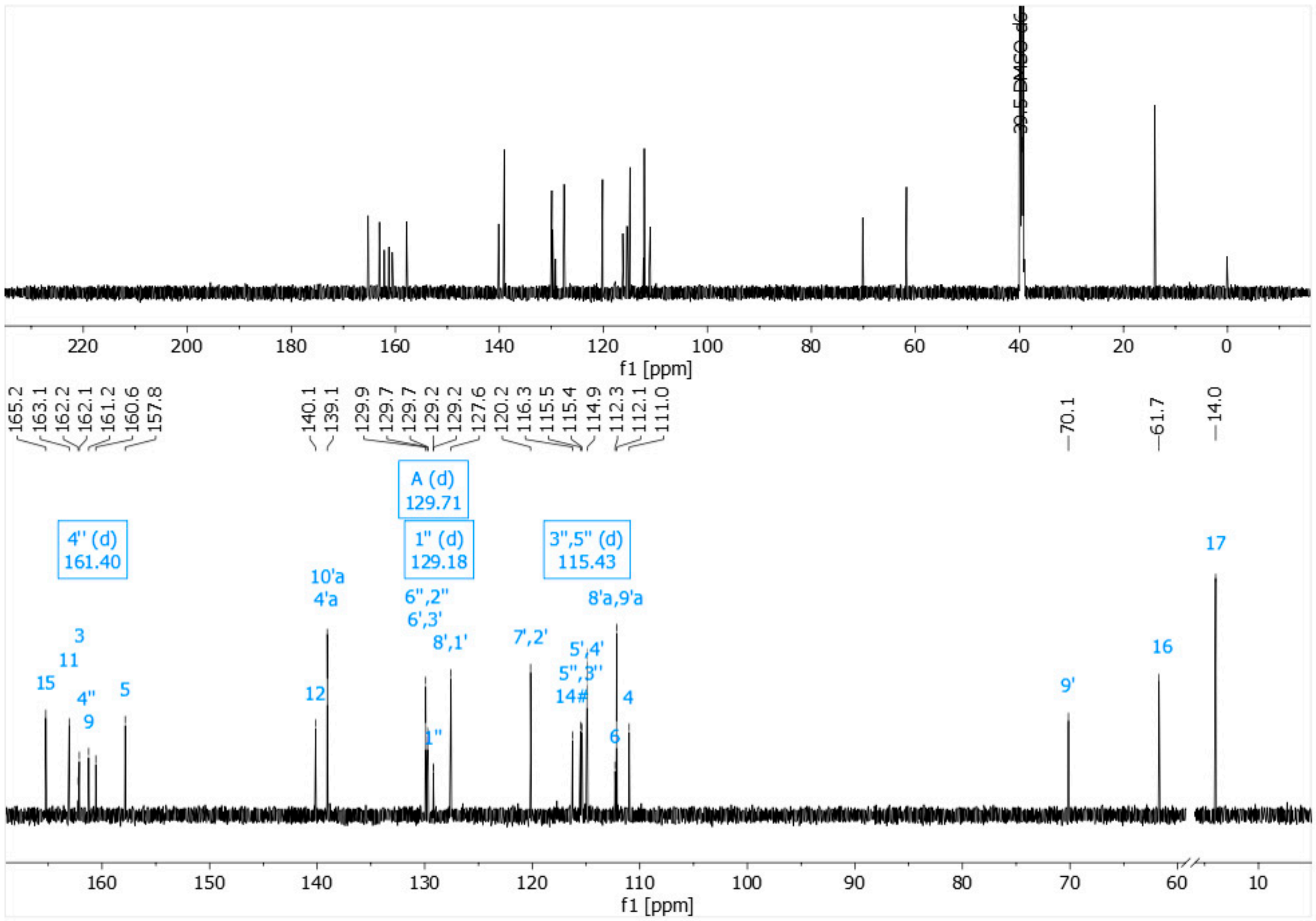

^{13}C NMR (150 MHz, DMSO-d$_6$) spectrum of derivative **8d**.

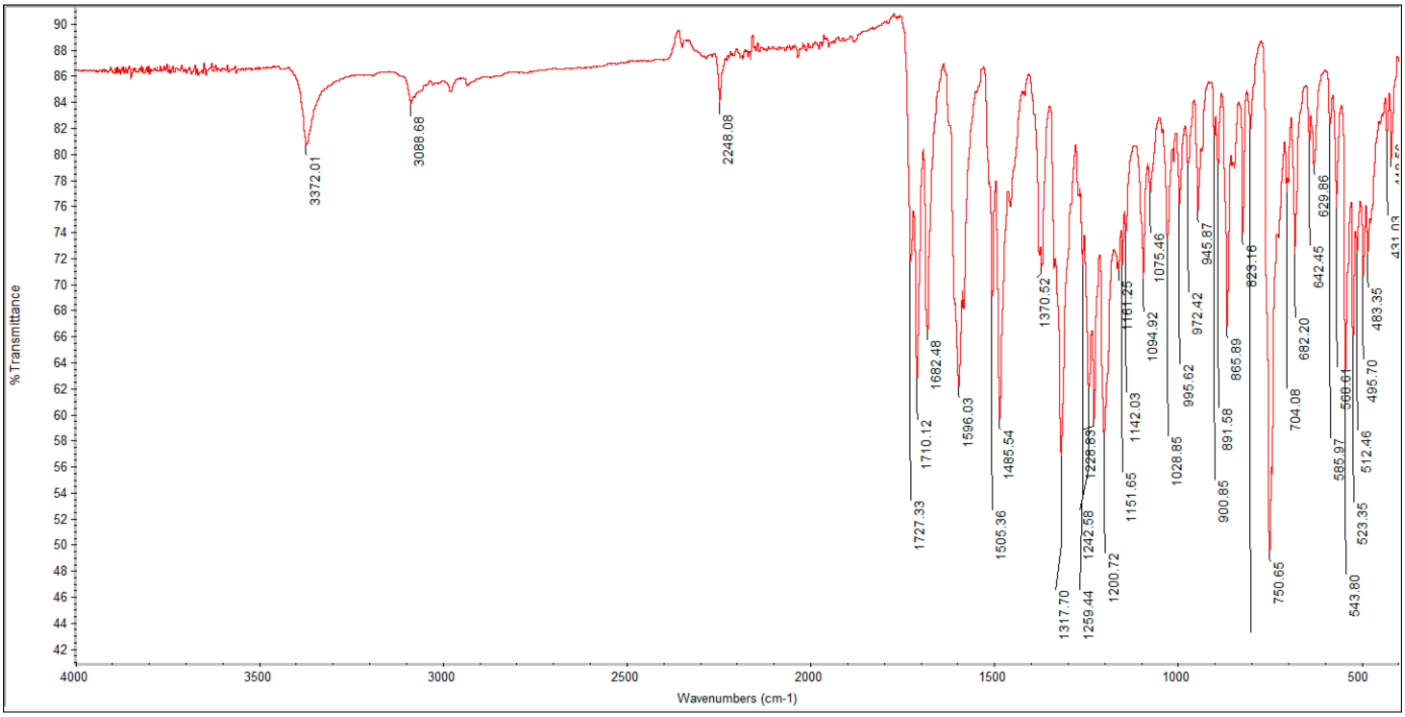

FTIR spectrum of derivative **8d**.

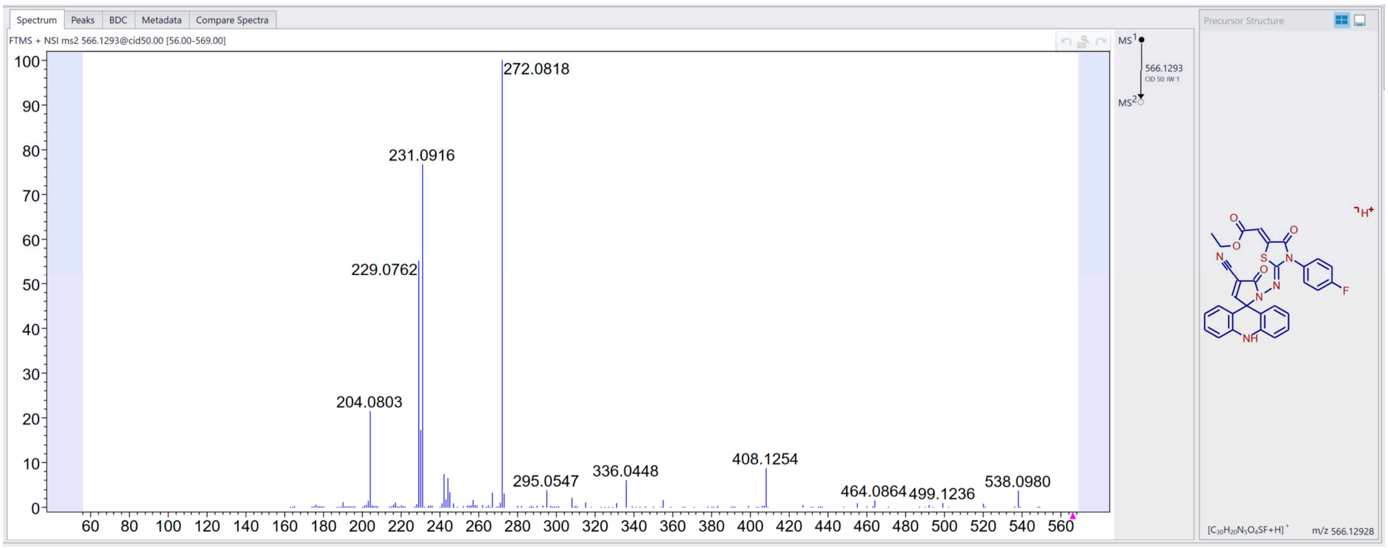

MS2 spectrum of derivative **8d**.

2.15 Ethyl 2-[(2*Z*,5*Z*)-2-({4'-cyano-5'-oxo-1',5'-dihydro-10*H*-spiro[acridine-9,2'-pyrrol]-1'-yl}imino)-3-(4-nitrophenyl)-4-oxo-1,3-thiazolidin-5-ylidene]acetate (8e)

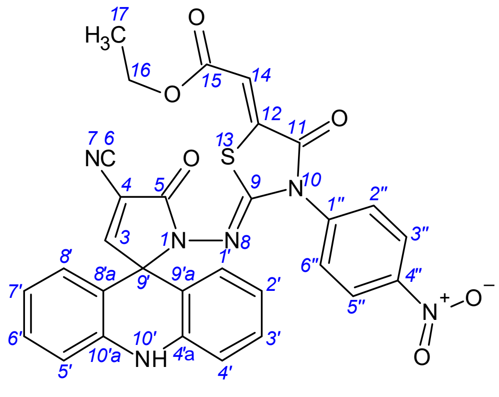

8e

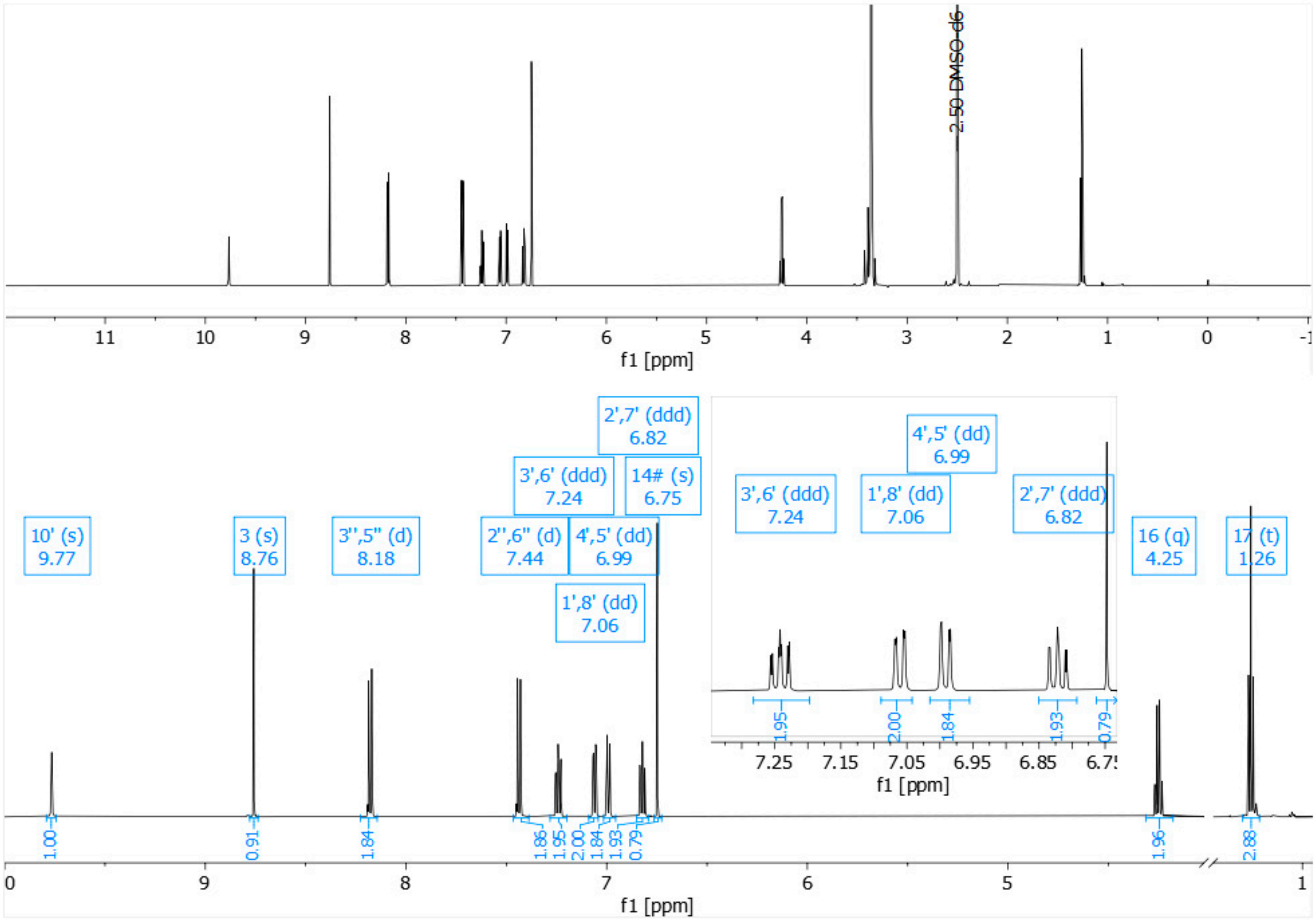

^{1}H NMR (600 MHz, DMSO-d$_6$) spectrum of derivative **8e**.

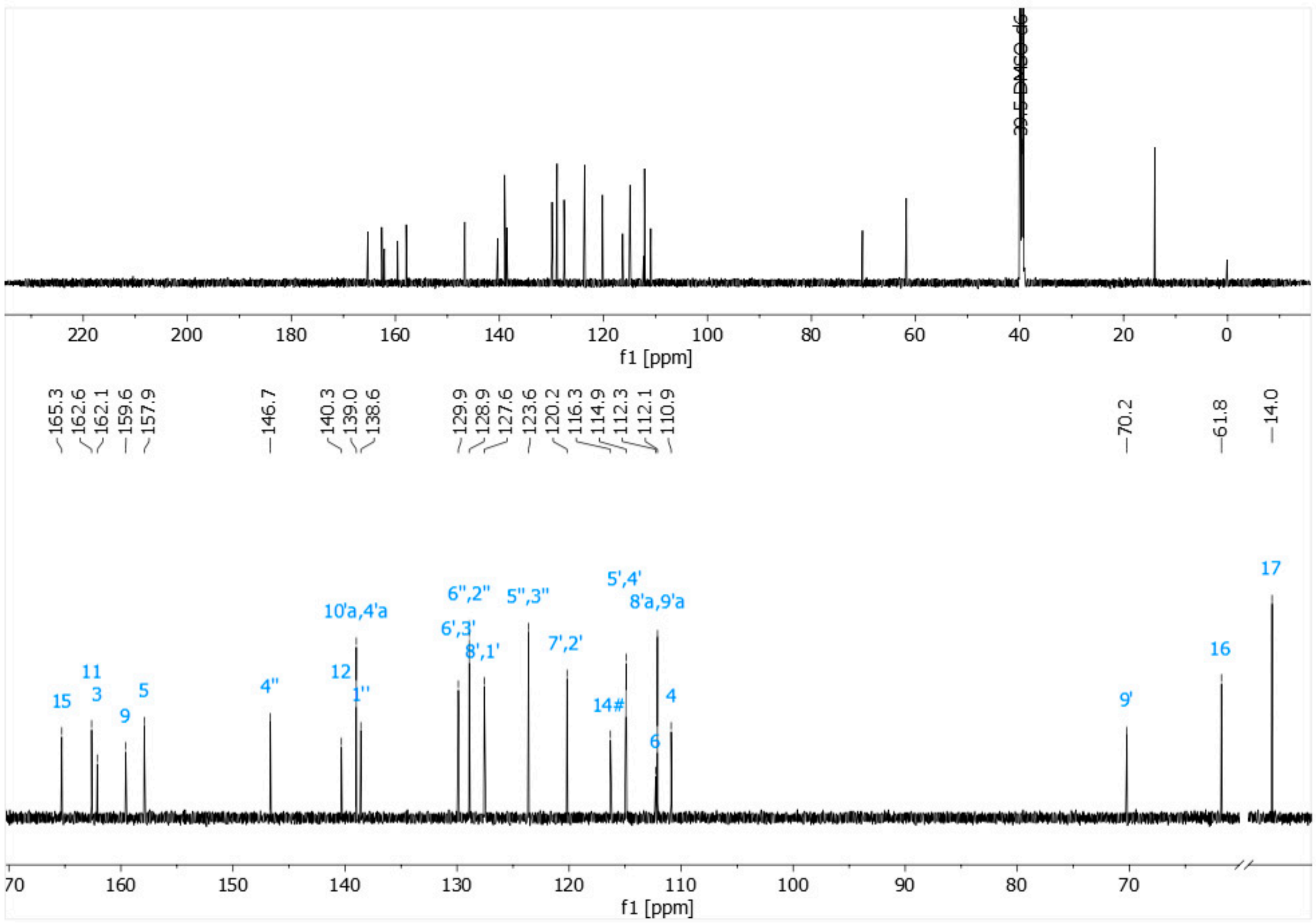

^{13}C NMR (150 MHz, DMSO-d_6) spectrum of derivative **8e**.

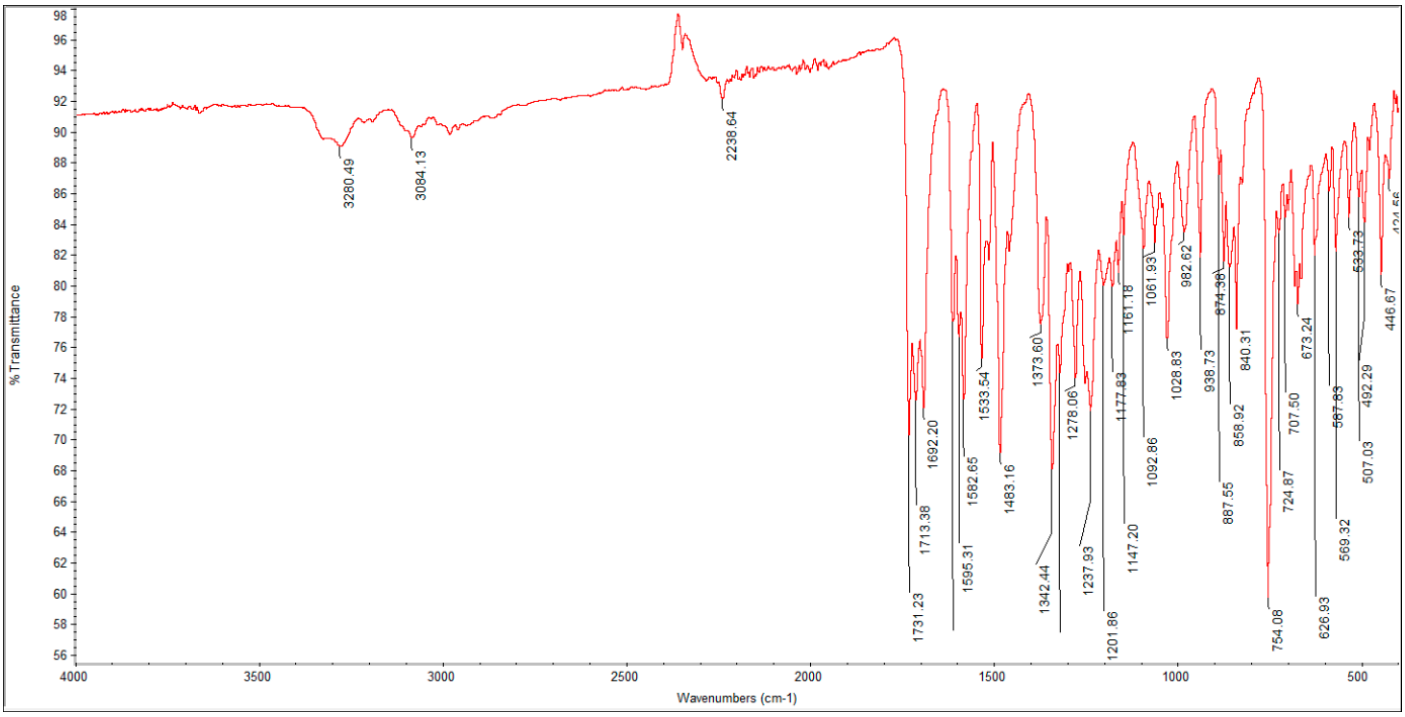

FTIR spectrum of derivative **8e**.

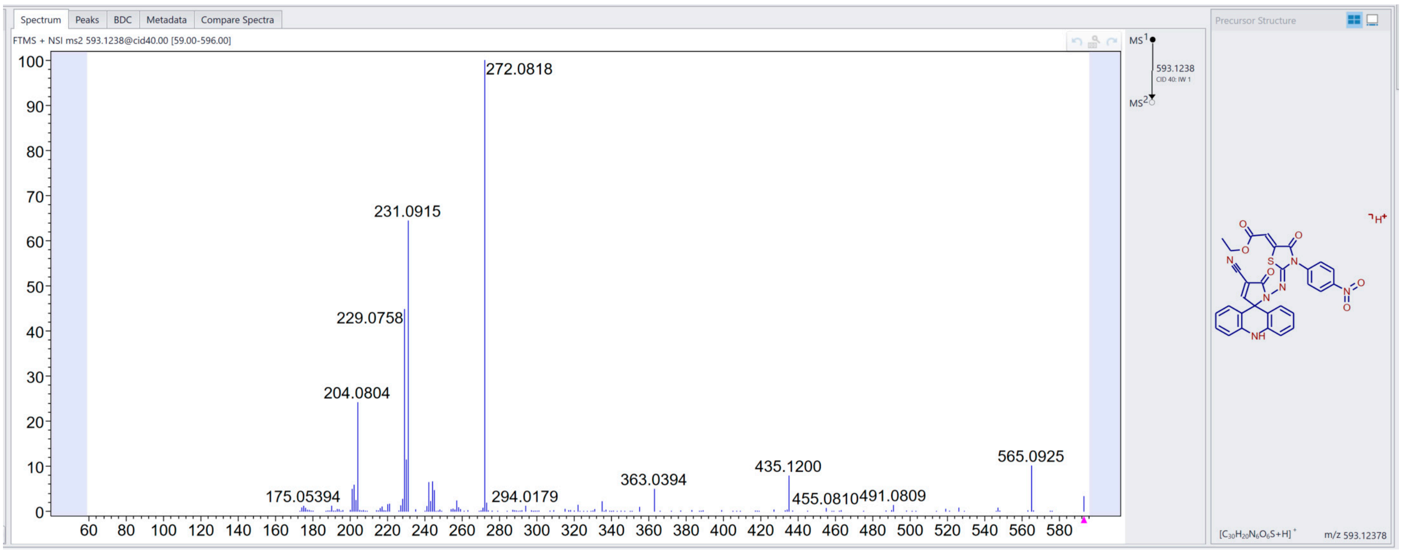

MS2 spectrum of derivative **8e**.

2.16 1'-{[(2*Z*)-5-Methyl-4-oxo-3-phenyl-1,3-thiazolidin-2-ylidene]amino}-5'-oxo-1',5'-dihydro-10*H*-spiro[acridine-9,2'-pyrrole]-4'-carbonitrile (**9a**)

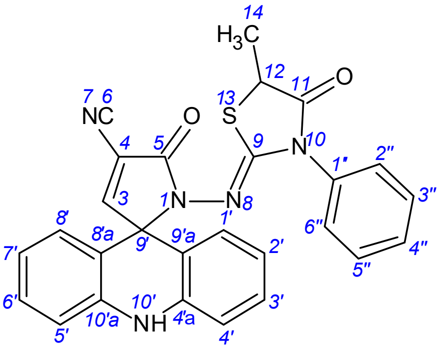

9a

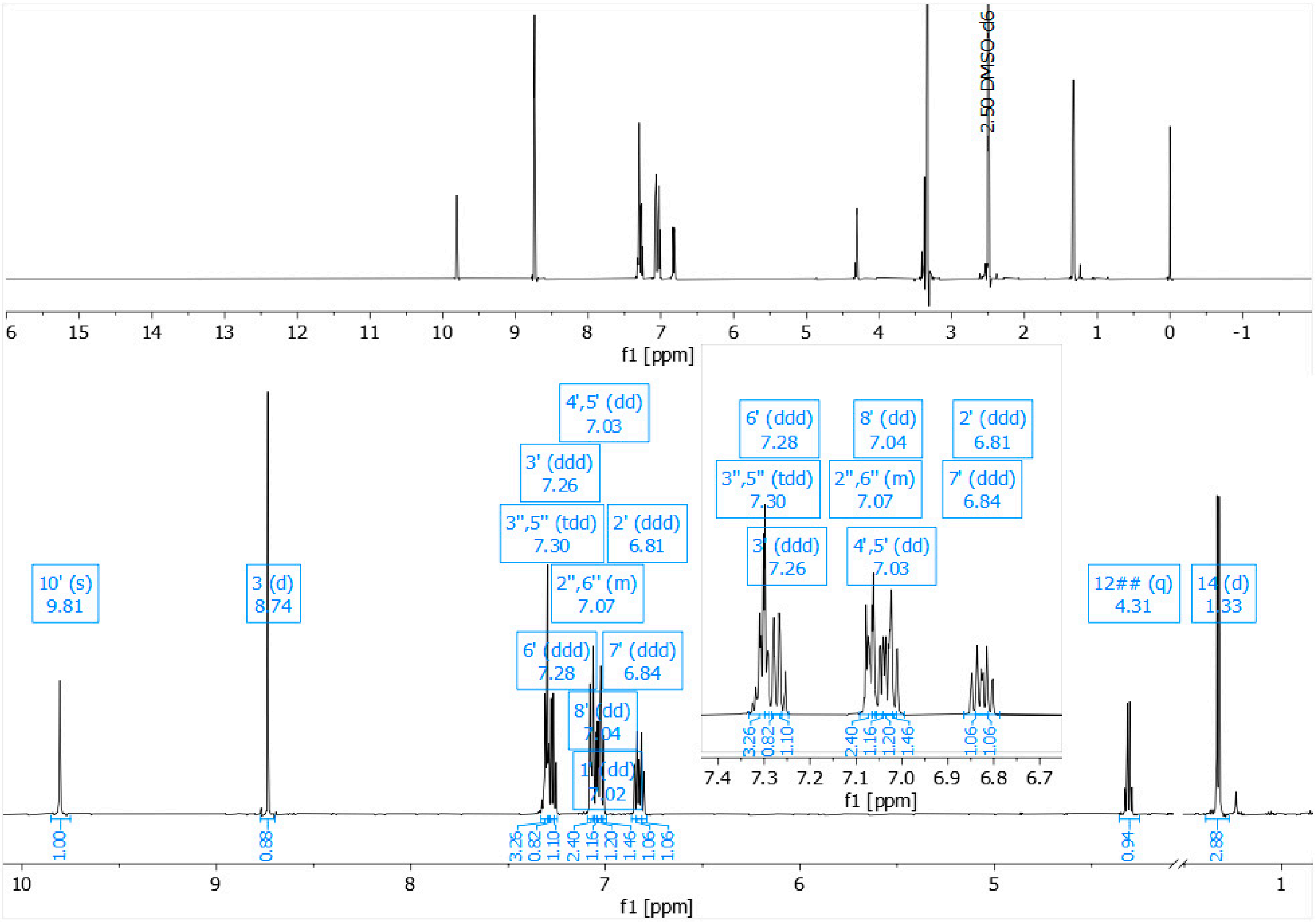

^{1}H NMR (600 MHz, DMSO-d$_6$) spectrum of derivative **9a**.

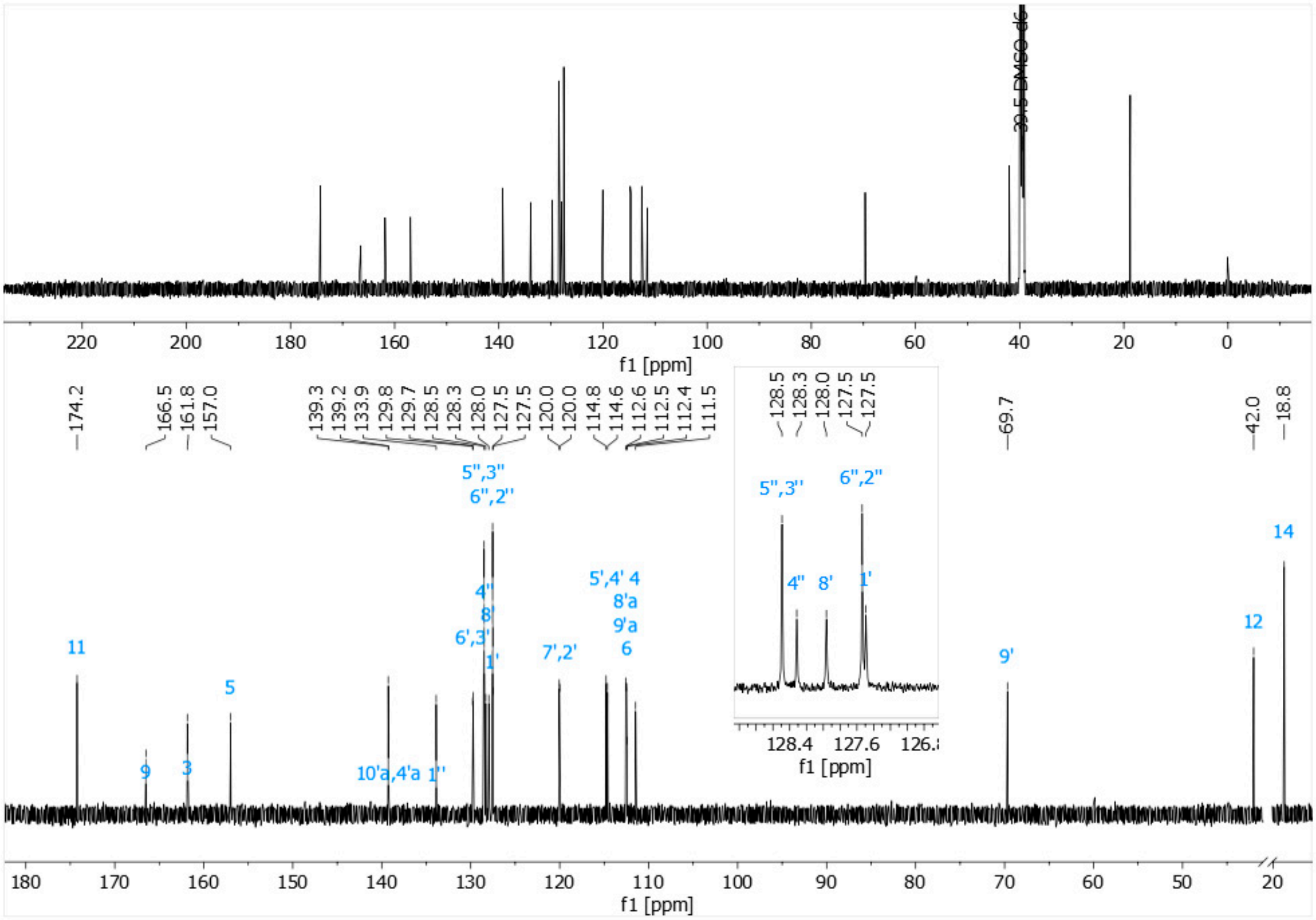

^{13}C NMR (150 MHz, DMSO-d_6) spectrum of derivative **9a**.

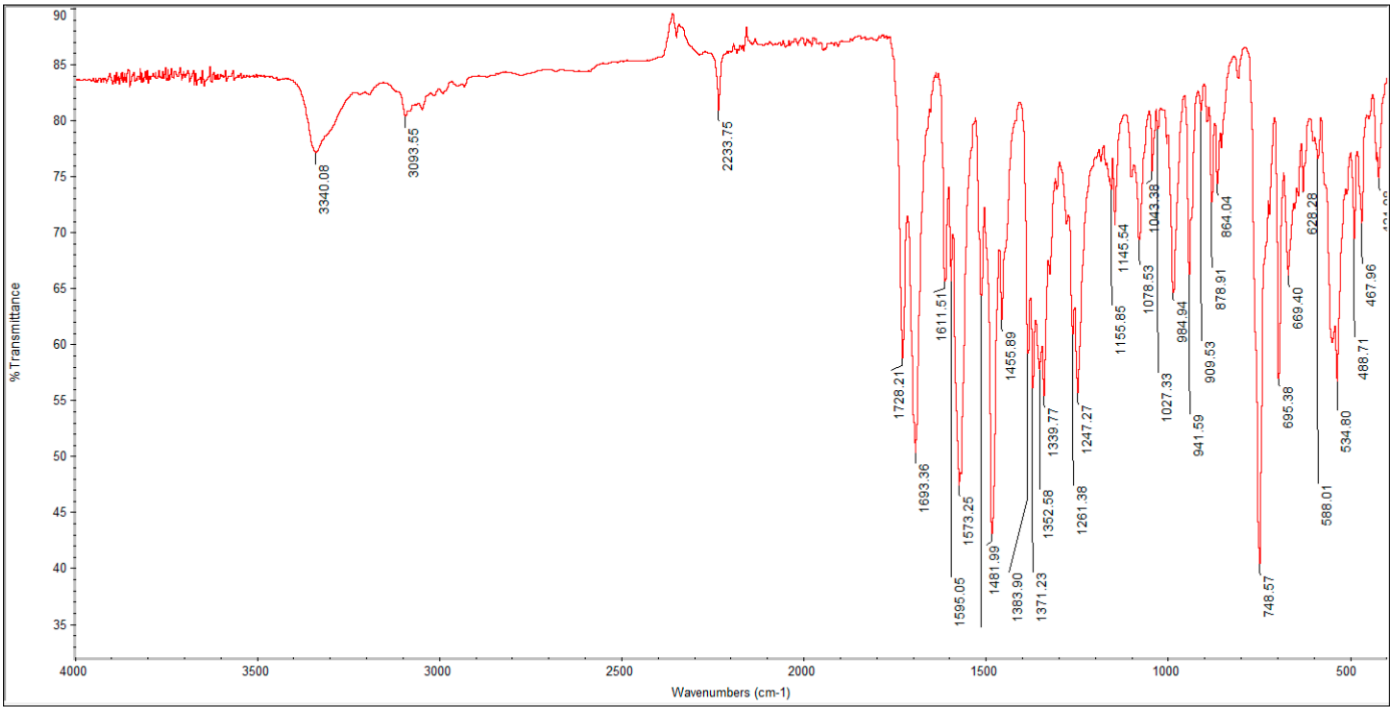

FTIR spectrum of derivative **9a**.

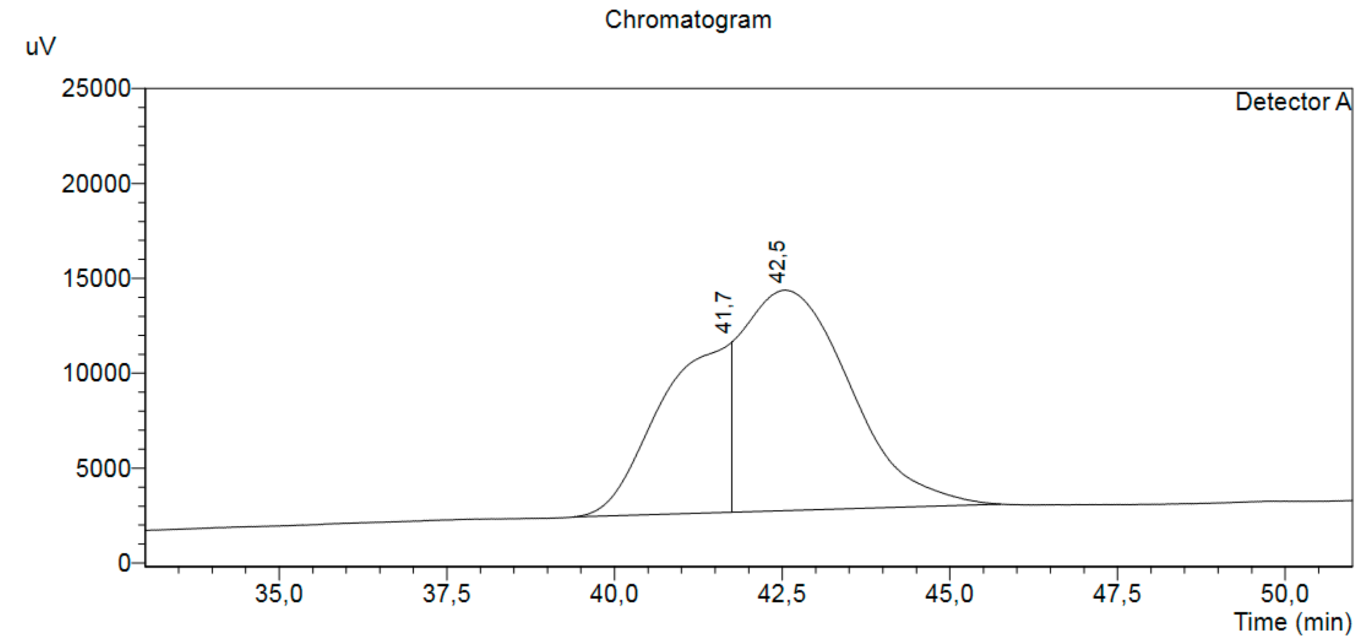

<Peak Table>

Detector A

Peak#	Ret. Time	Area	Height	Conc.	Unit	Area%
1	41,746	653960	8950	33,533		33,533
2	42,544	1296223	11625	66,467		66,467
Total		1950183	20575			100,000

Chiral HPLC chromatogram of compound **9a**. Chiralpak IA column; flow rate of 0.5 mL/min; temperature 25 °C; *n*-hexane/acetone (75/25, *v*/*v*).

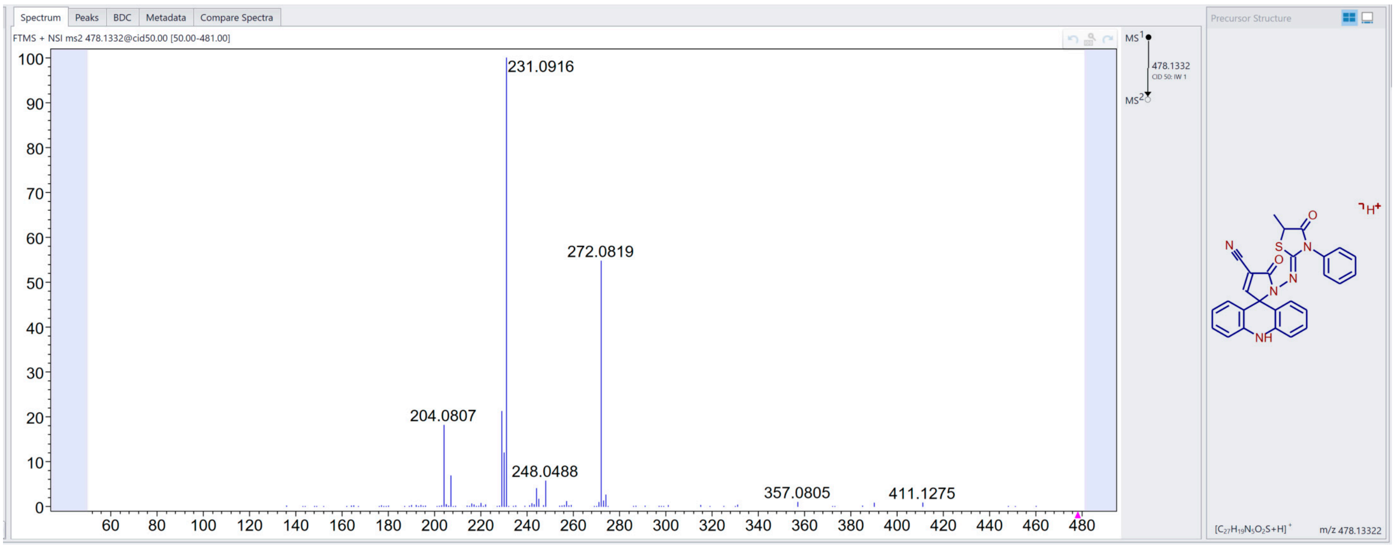

MS2 spectrum of derivative **9a**.

2.17 1'-{[(2*Z*)-3-(4-Methoxyphenyl)-5-methyl-4-oxo-1,3-thiazolidin-2-ylidene]amino}-5'-oxo-1',5'-dihydro-10*H*-spiro[acridine-9,2'-pyrrole]-4'-carbonitrile (9b)

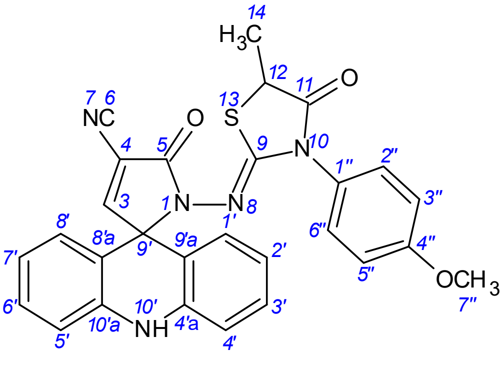

9b

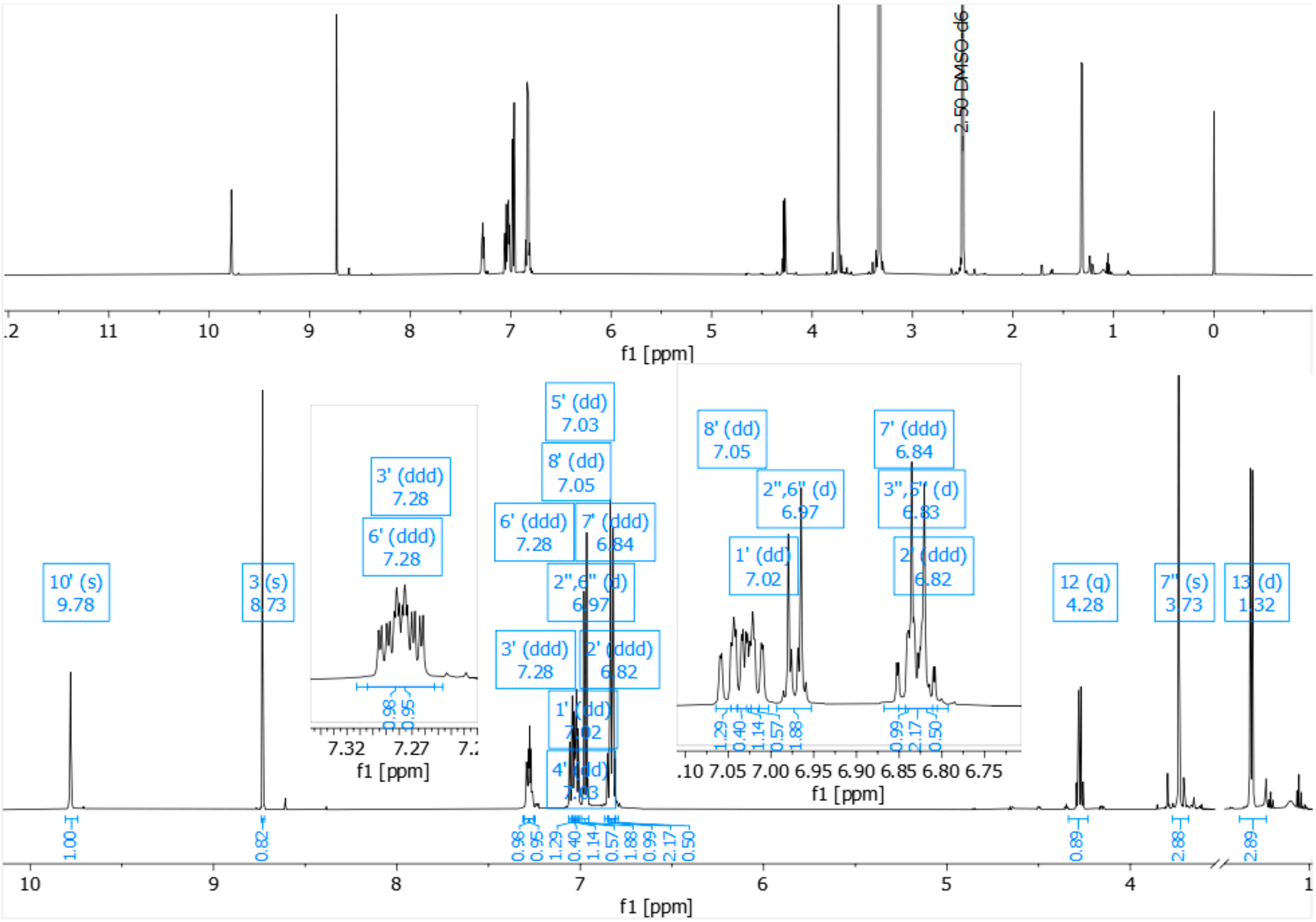

^{1}H NMR (600 MHz, DMSO-d$_6$) spectrum of derivative **9b**.

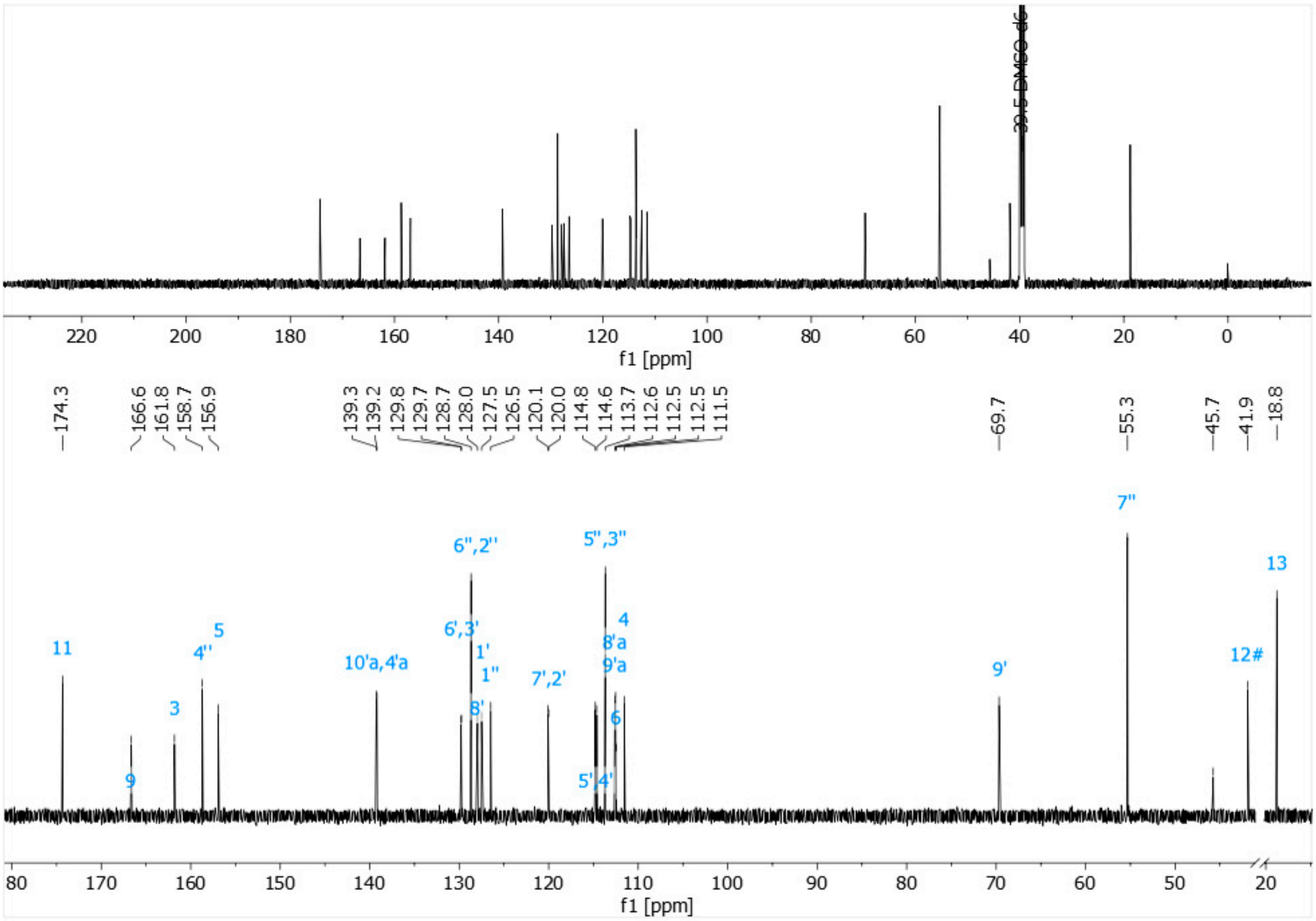

^{13}C NMR (150 MHz, DMSO-d6) spectrum of derivative **9b**.

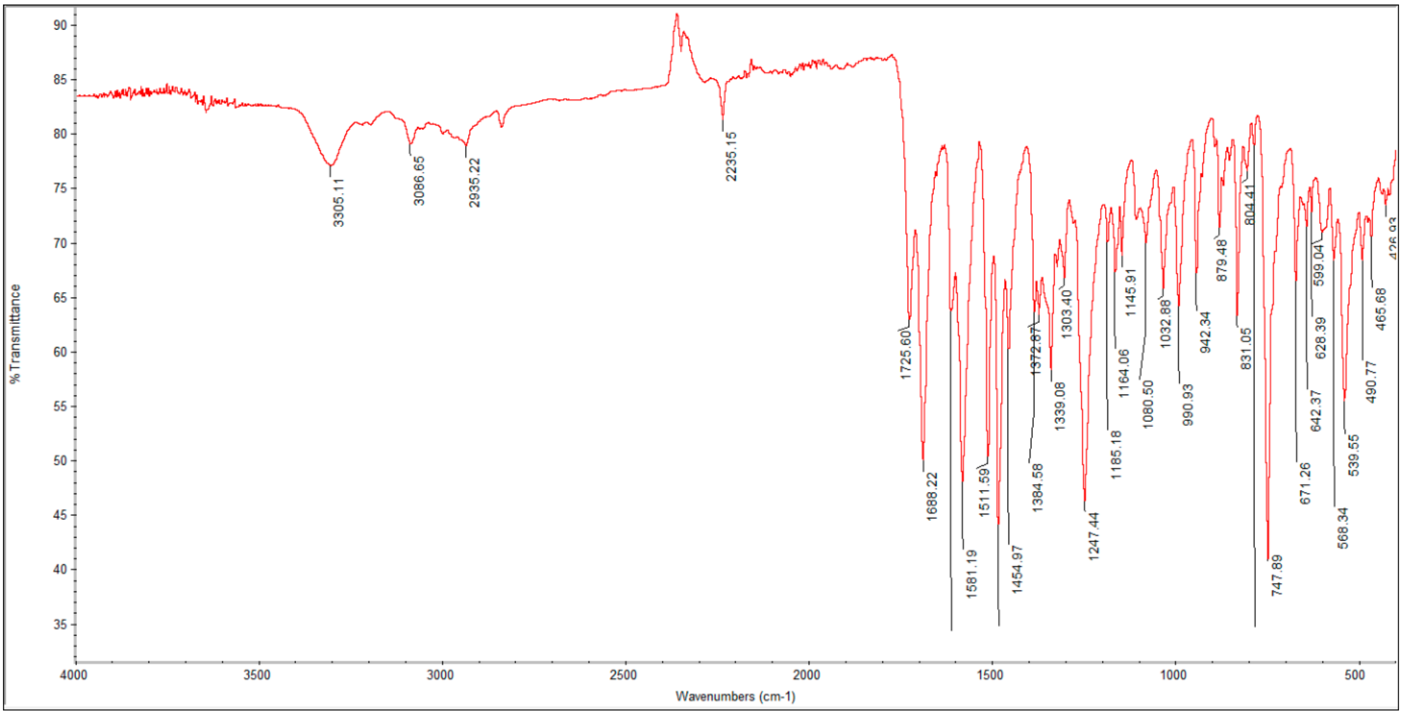

FTIR spectrum of derivative **9b**.

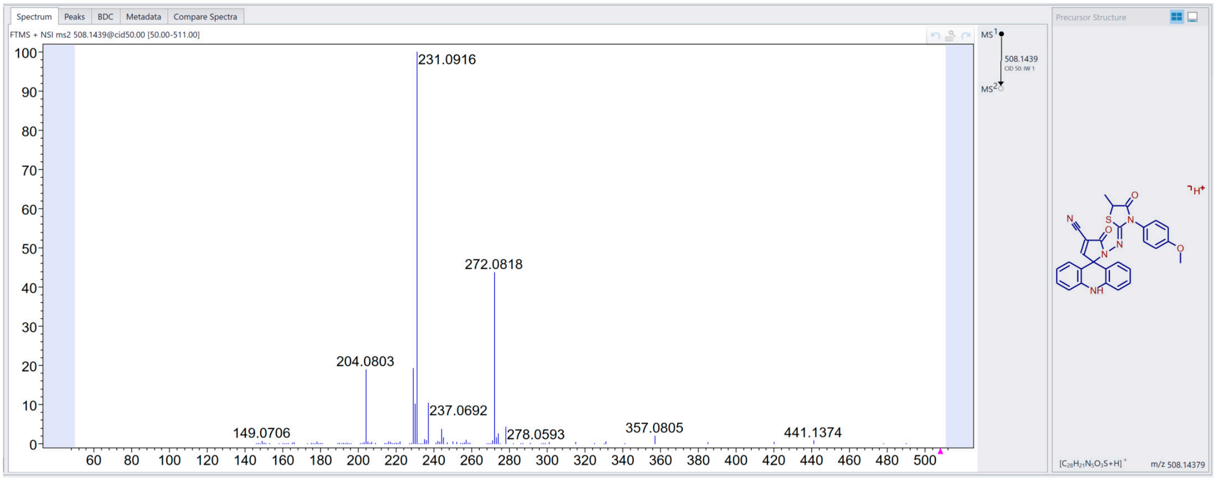

MS2 spectrum of derivative **9b**.

2.18 1'-{[(2Z)-3-(3-Methoxyphenyl)-5-methyl-4-oxo-1,3-thiazolidin-2-ylidene]amino}-5'-oxo-1',5'-dihydro-10H-spiro[acridine-9,2'-pyrrole]-4'-carbonitrile (**9c**)

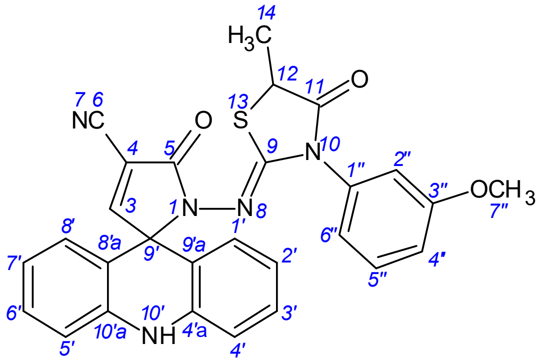

9c

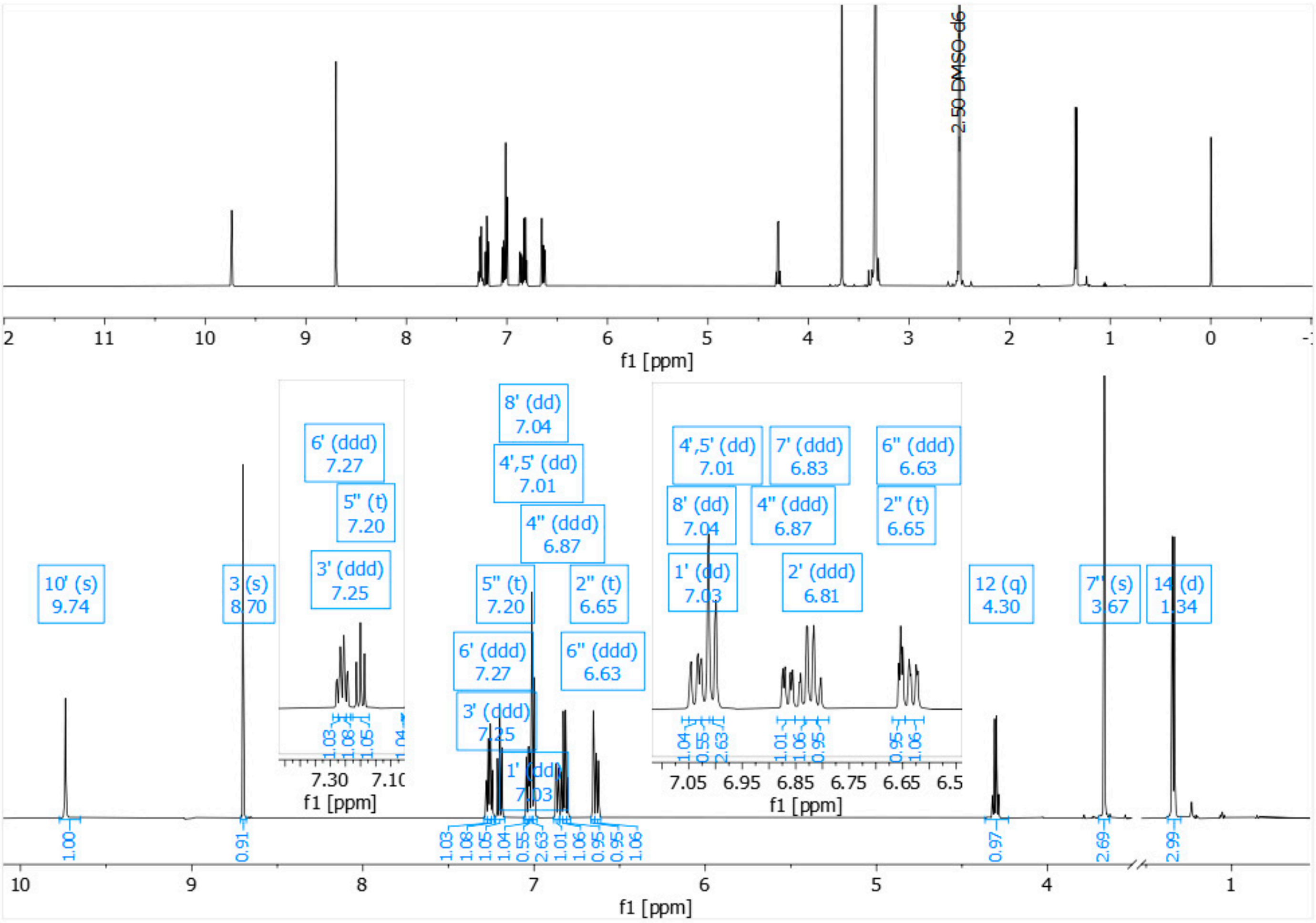

^{1}H NMR (600 MHz, DMSO-d$_6$) spectrum of derivative **9c**.

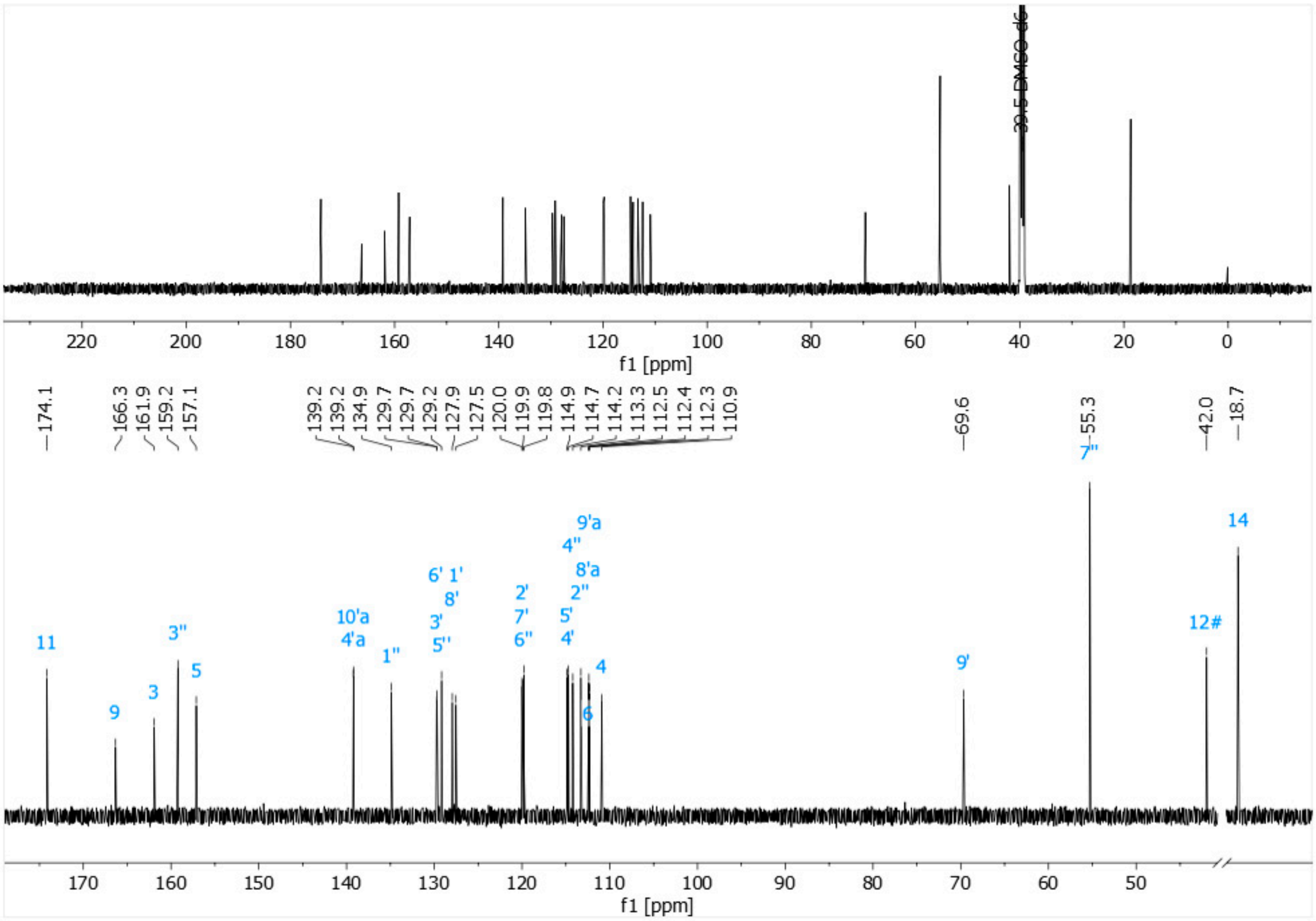

^{13}C NMR (150 MHz, DMSO-d$_6$) spectrum of derivative **9c**.

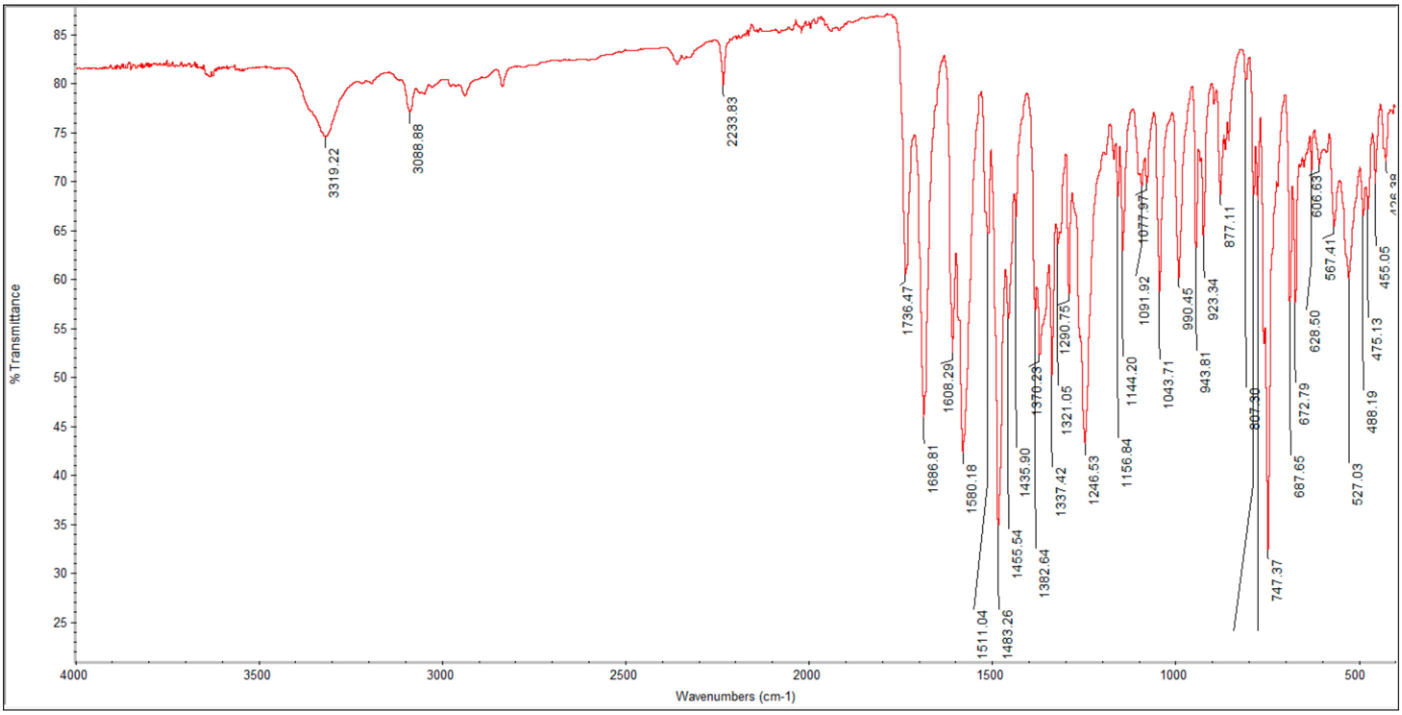

FTIR spectrum of derivative **9c**.

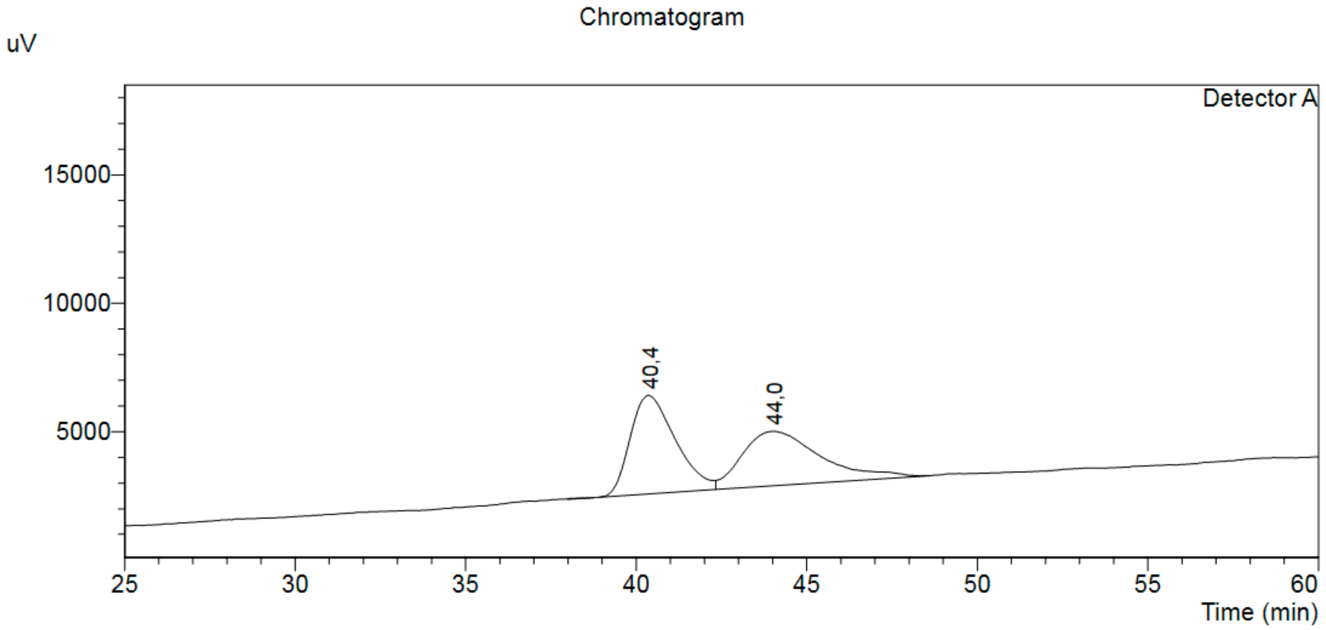

<Peak Table>

Detector A

Peak#	Ret. Time	Area	Height	Conc.	Unit	Area%
1	40,382	354177	3687	51,012		51,012
2	44,023	340130	2090	48,988		48,988
Total		694306	5777			100,000

Chiral HPLC chromatogram of compound **9c**. Chiralpak IA column; flow rate of 0.5 mL/min; temperature 25 °C; *n*-hexane/acetone (85/15, *v*/*v*)

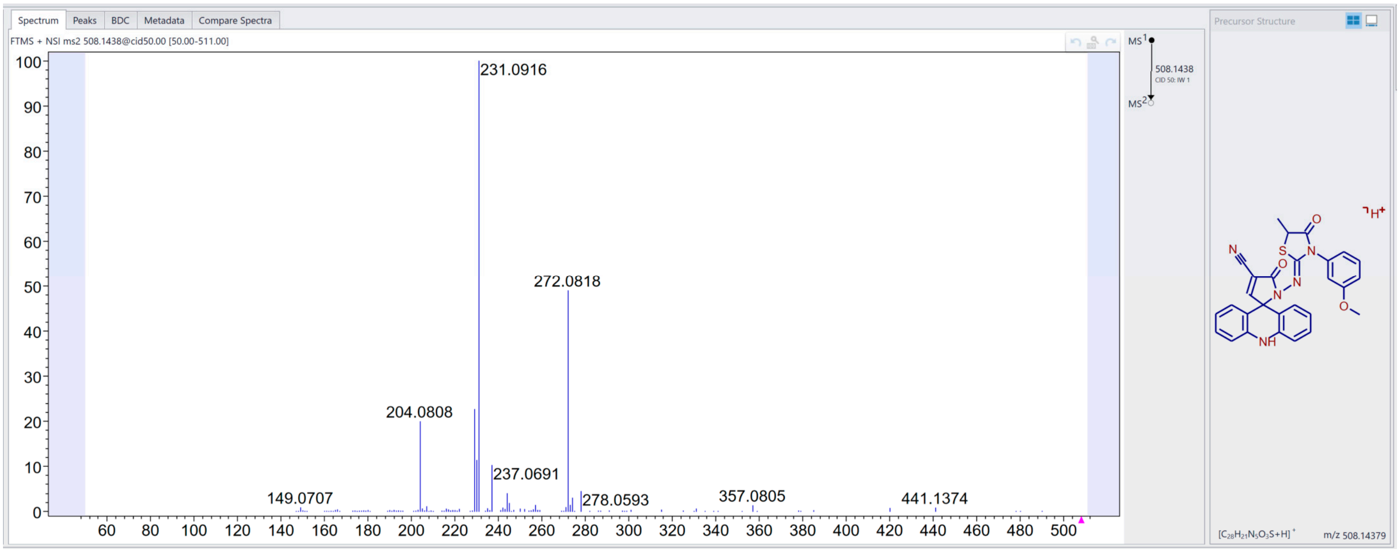

MS2 spectrum of derivative **9c**.

2.19 1'-{[(2*Z*)-3-(4-Fluorophenyl)-5-methyl-4-oxo-1,3-thiazolidin-2-ylidene]amino}-5'-oxo-1',5'-dihydro-10*H*-spiro[acridine-9,2'-pyrrole]-4'-carbonitrile (**9d**)

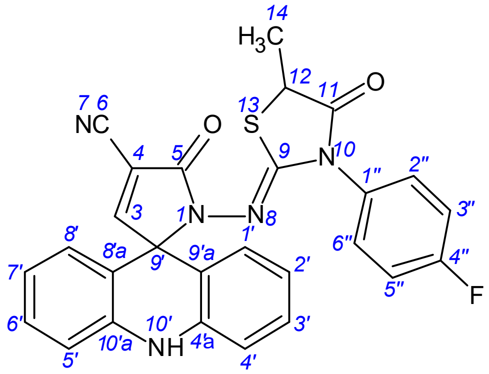

9d

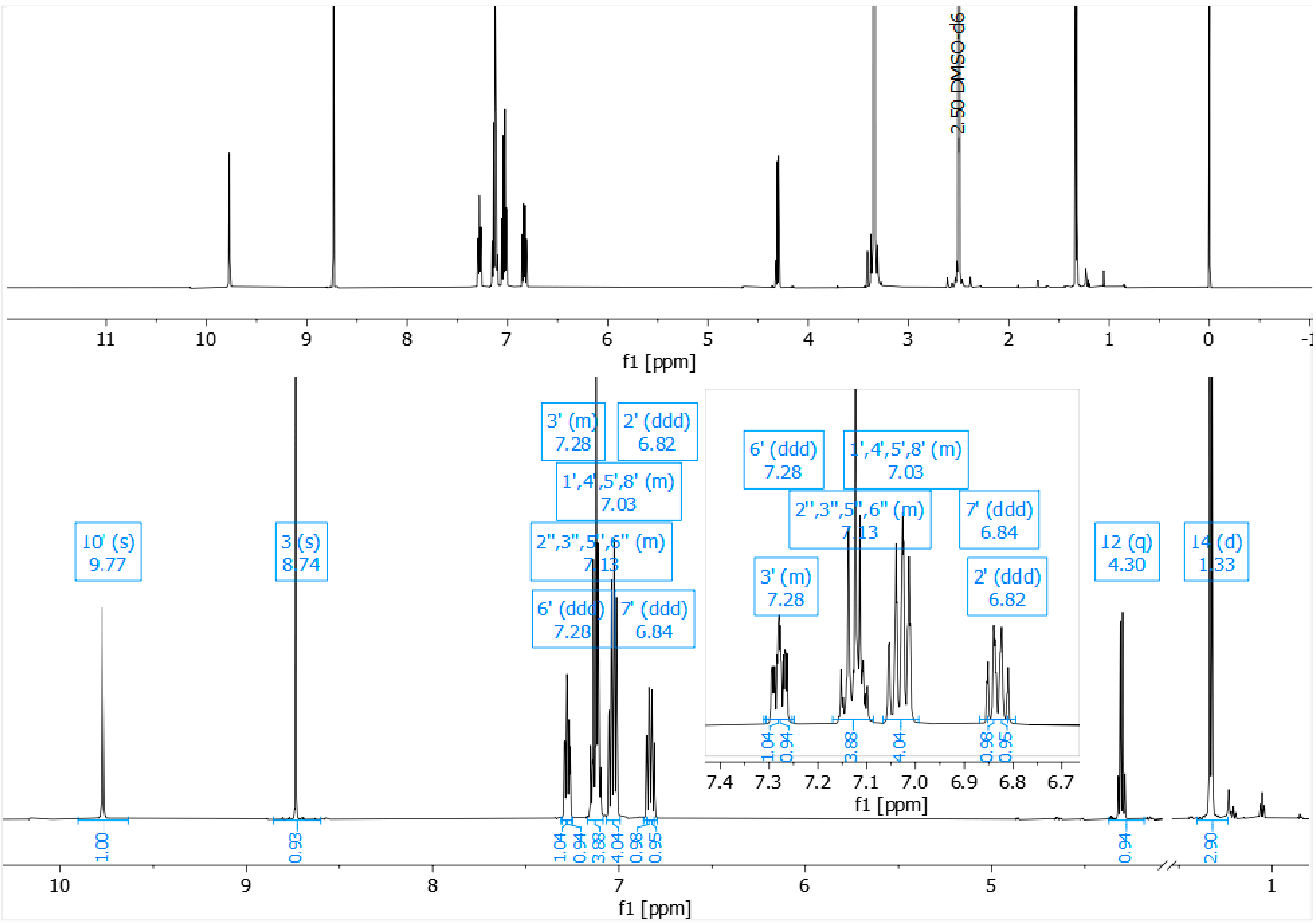

^{1}H NMR (600 MHz, DMSO-d$_6$) spectrum of derivative **9d**.

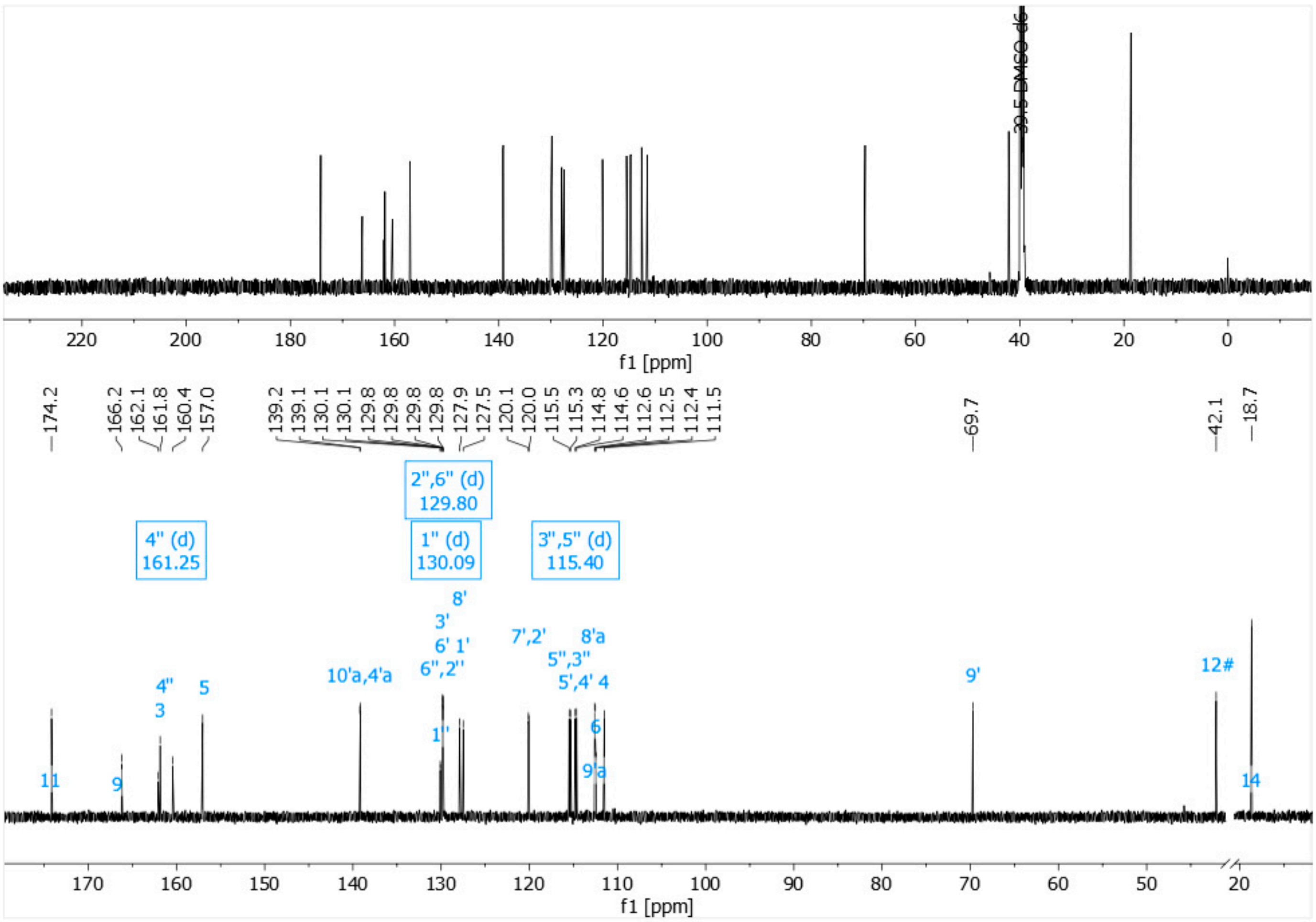

^{13}C NMR (150 MHz, DMSO-d$_6$) spectrum of derivative **9d**.

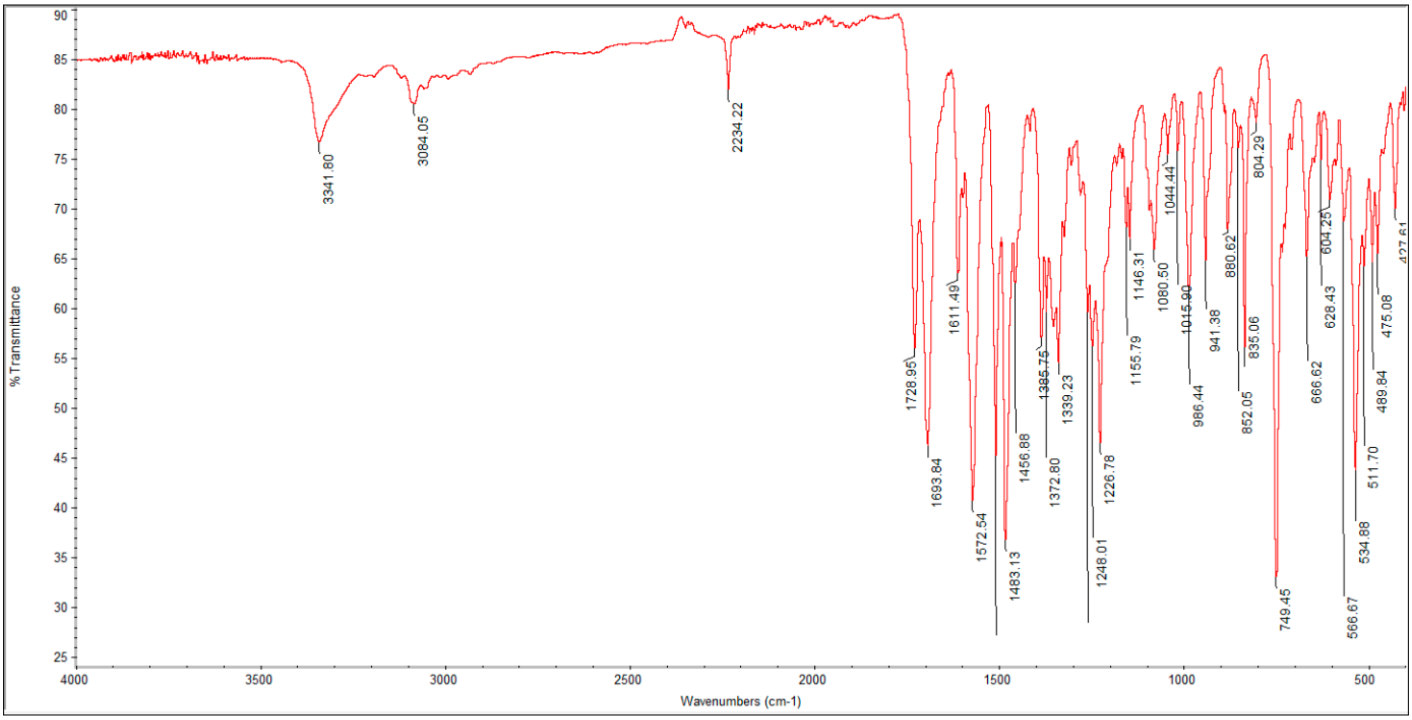

FTIR spectrum of derivative **9d**.

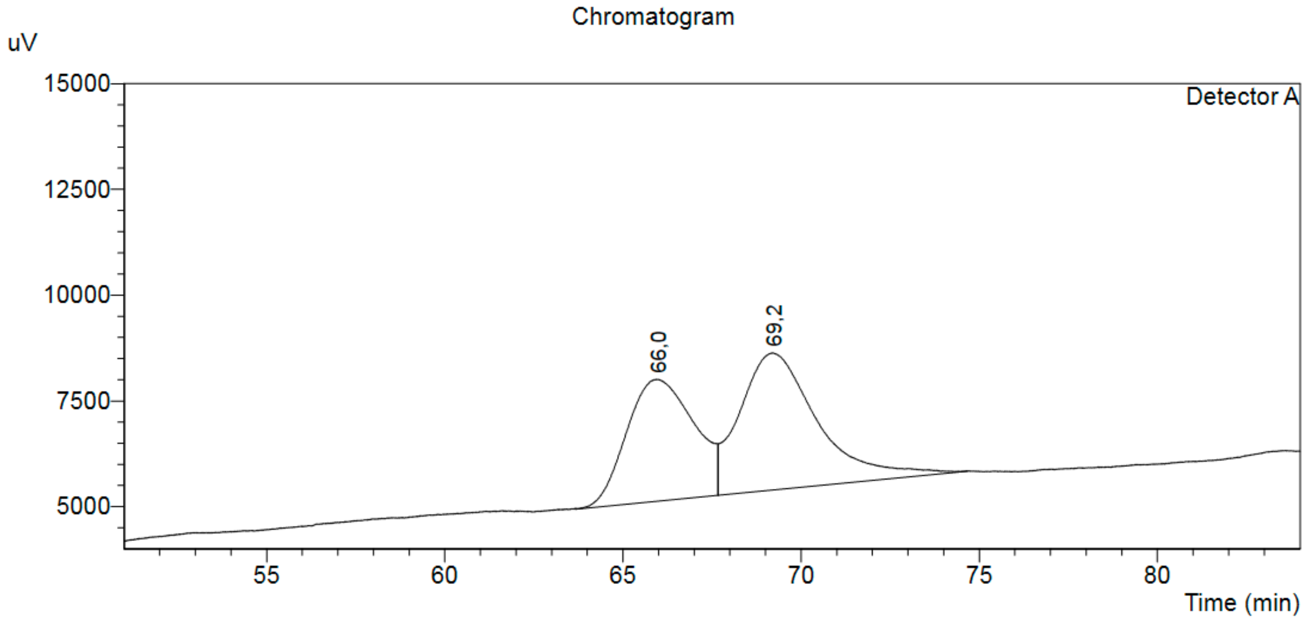

<Peak Table>

Detector A

Peak#	Ret. Time	Area	Height	Conc.	Unit	Mark	Name
1	65,962	378532	2841	43,368			
2	69,209	494304	3215	56,632		V	
Total		872835	6056				

Chiral HPLC chromatogram of compound **9d**. Chiralpak IA column; flow rate of 0.5 mL/min; temperature 25 °C; *n*-hexane/acetone (85/15, *v*/*v*)

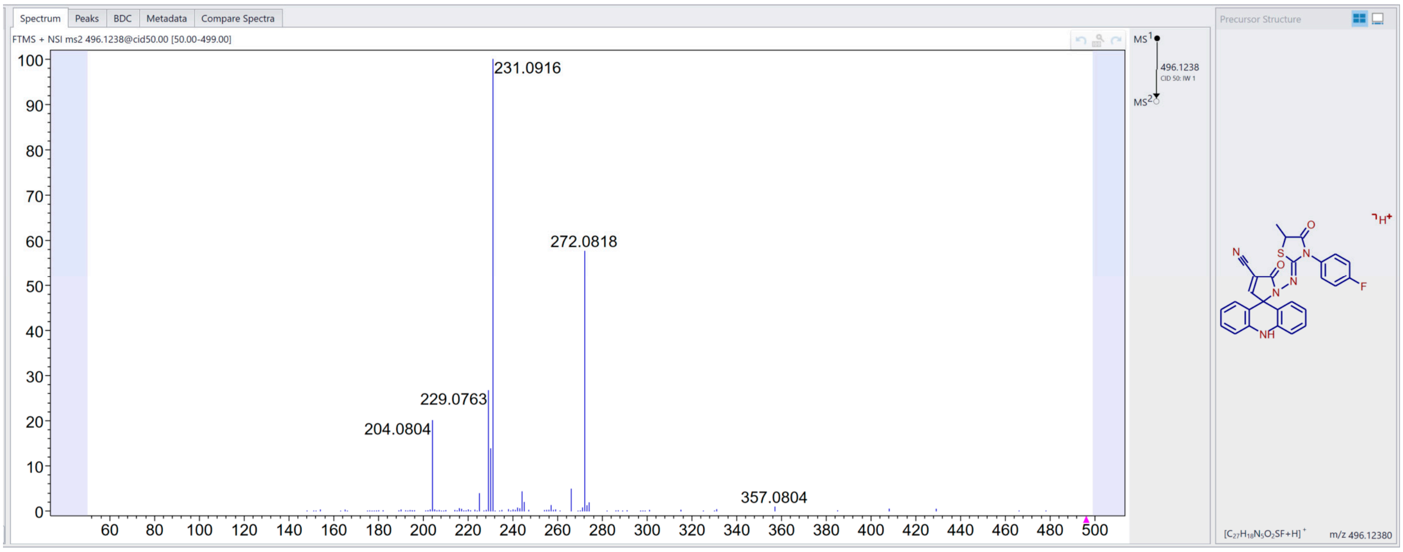

MS2 spectrum of derivative **9d**.

2.20 1'-{[(2*Z*)-5-Methyl-3-(4-nitrophenyl)-4-oxo-1,3-thiazolidin-2-ylidene]amino}-5'-oxo-1',5'-dihydro-10*H*-spiro[acridine-9,2'-pyrrole]-4'-carbonitrile (**9e**)

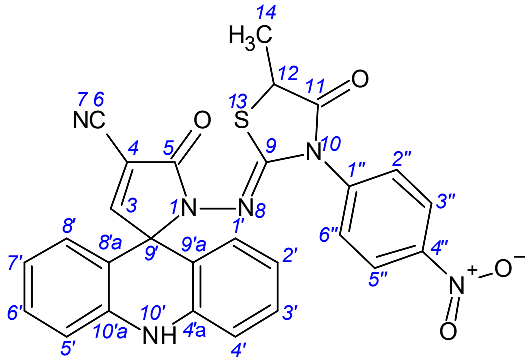

9e

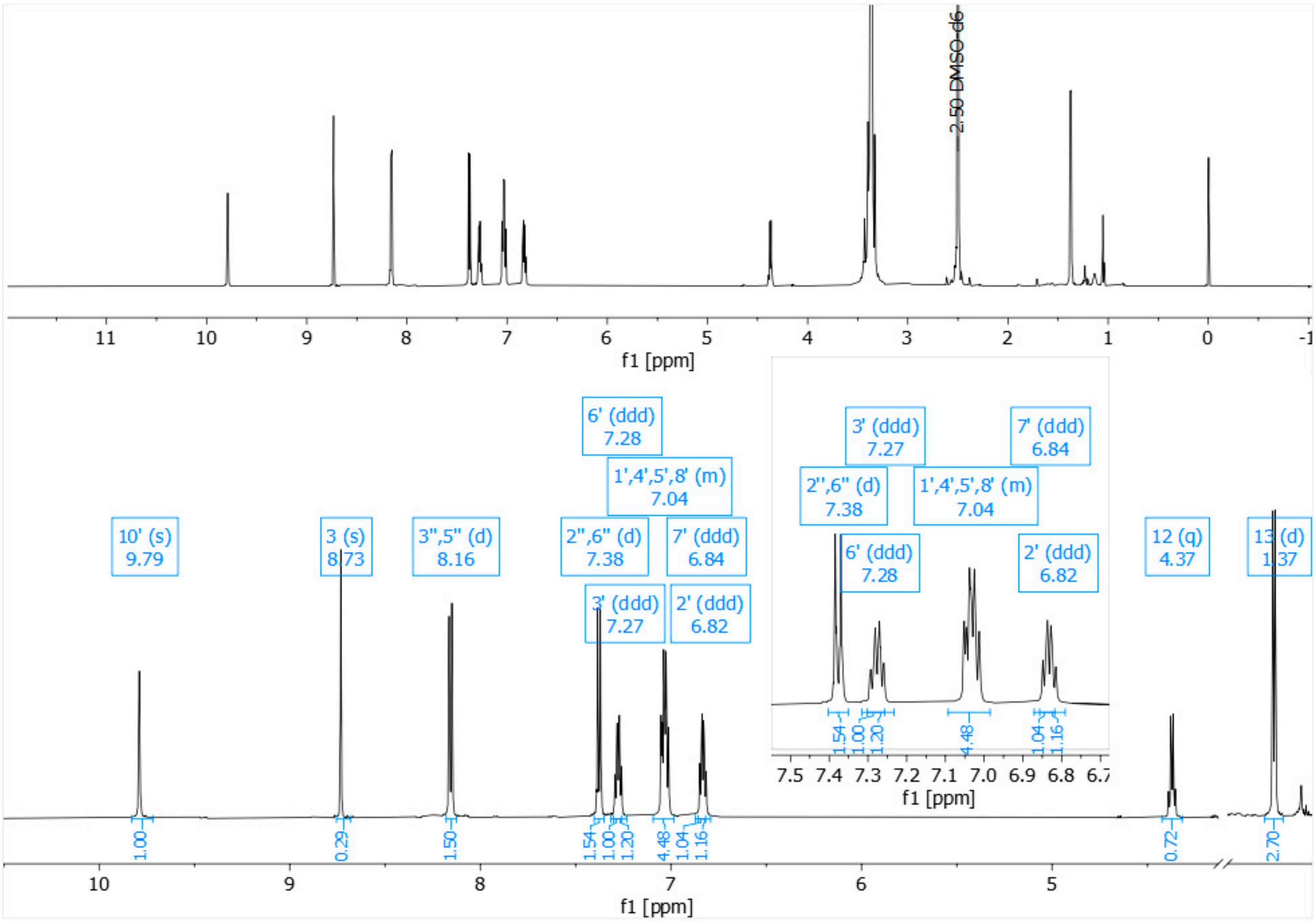

^{1}H NMR (600 MHz, DMSO-d$_6$) spectrum of derivative **9e**.

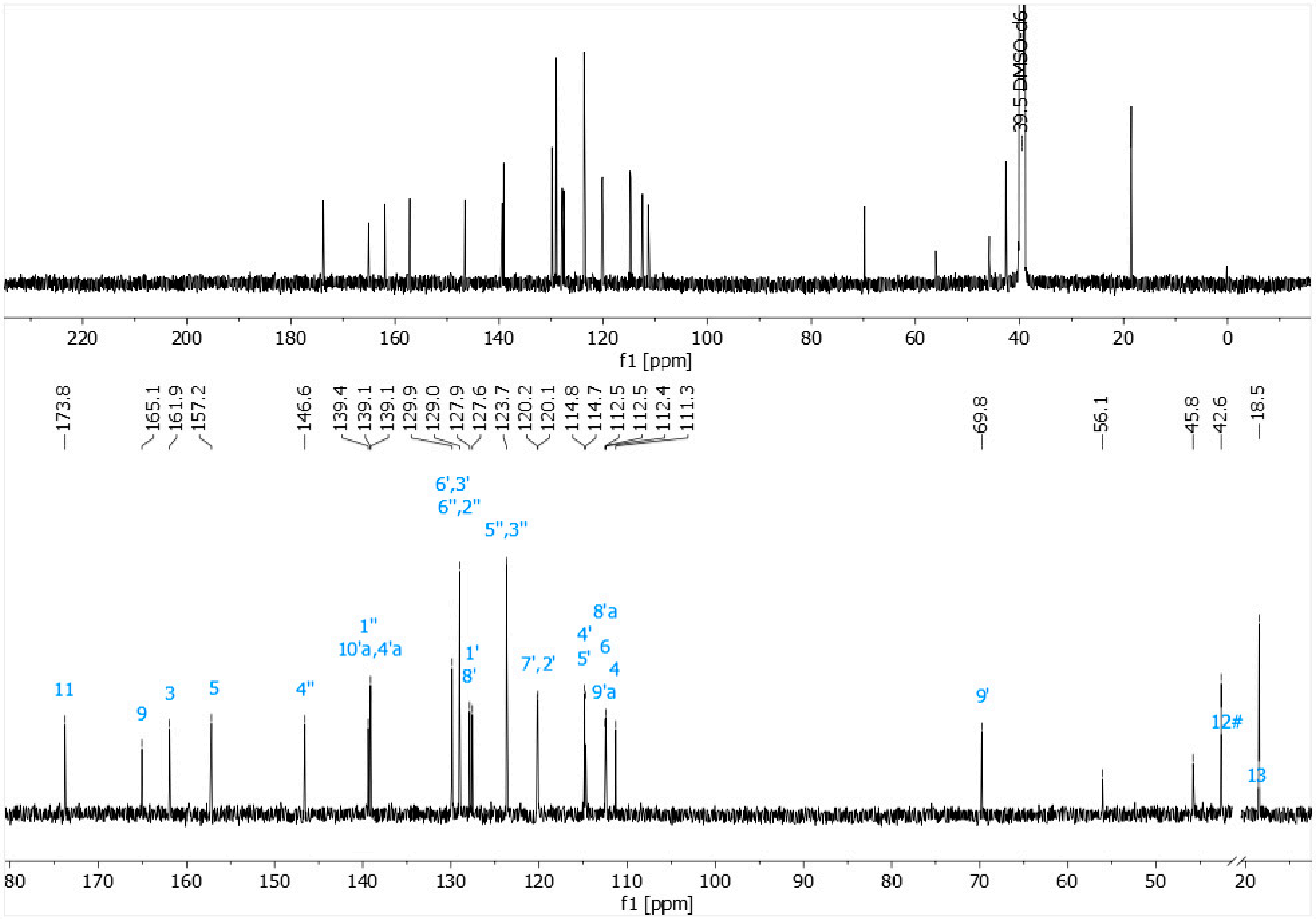

^{13}C NMR (150 MHz, DMSO-d$_6$) spectrum of derivative **9e**.

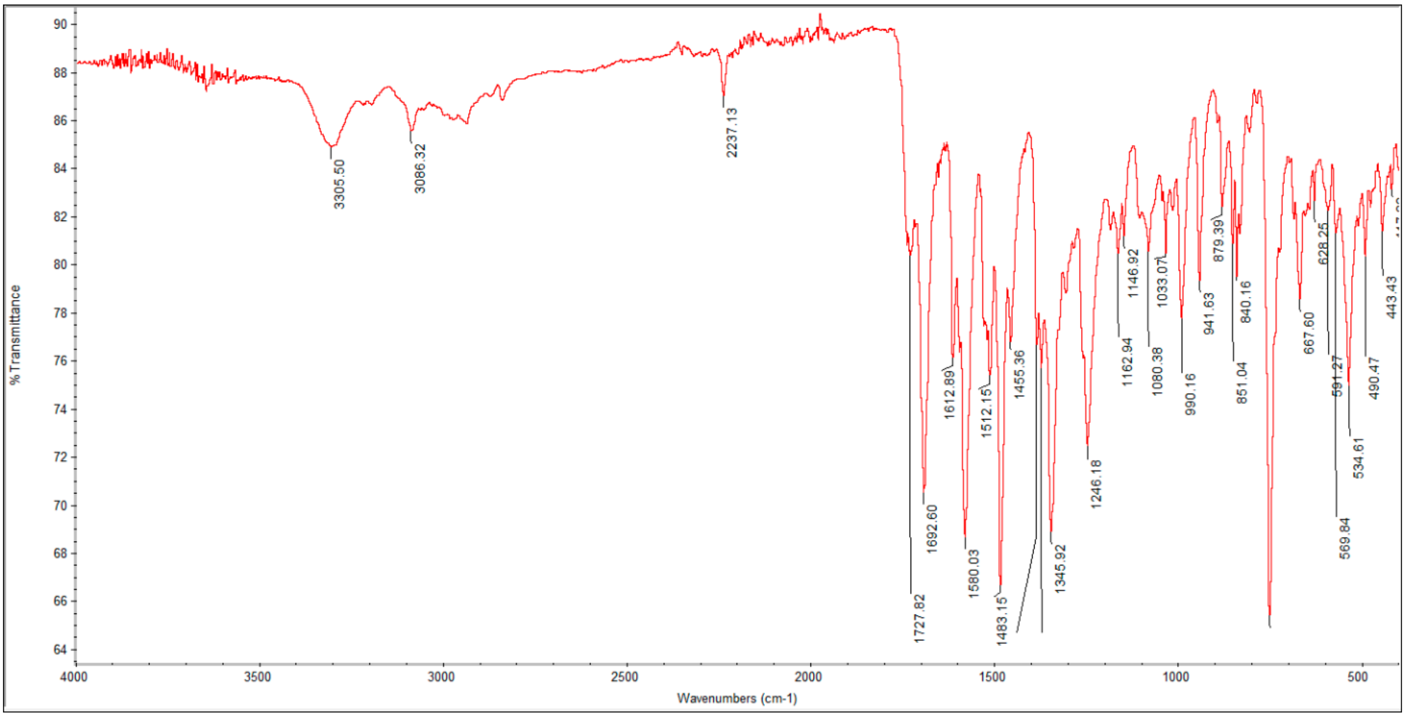

FTIR spectrum of derivative **9e**.

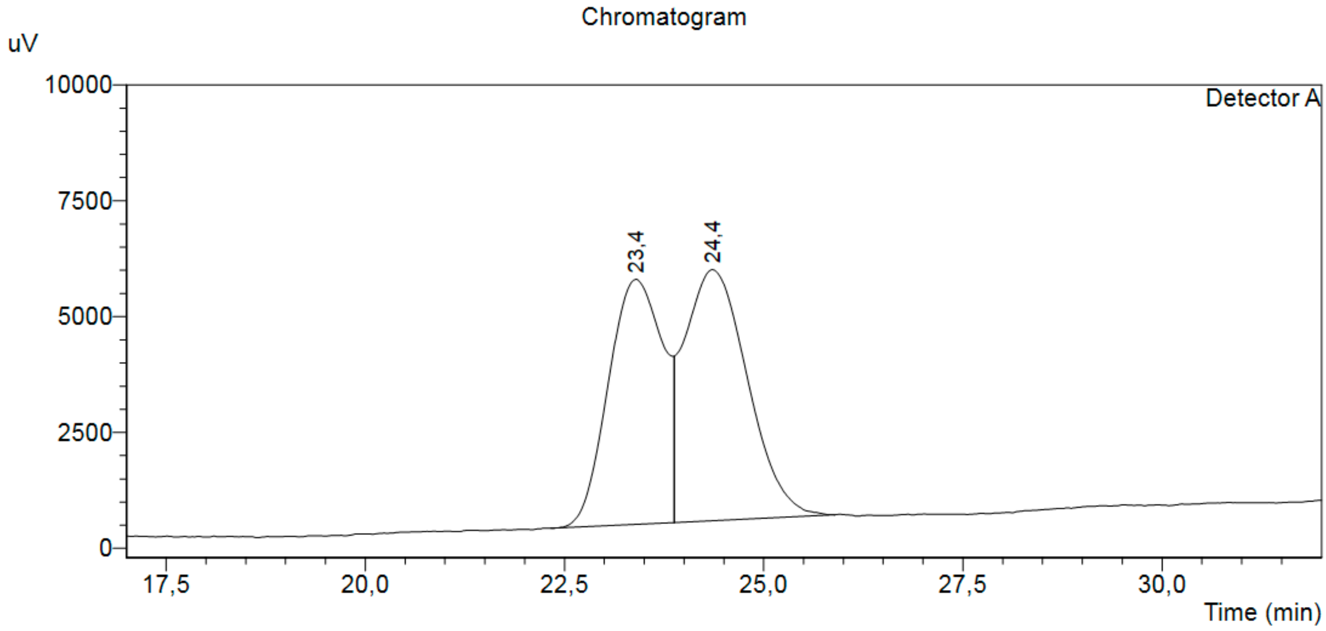

<Peak Table>

Detector A

Peak#	Ret. Time	Area	Height	Conc.	Unit	Area%
1	23,395	253680	5281	45,935		45,935
2	24,358	298576	5414	54,065		54,065
Total		552256	10694			100,000

Chiral HPLC chromatogram of compound **9e**. Chiralpak IA column; flow rate of 0.5 mL/min; temperature 25 °C; *n*-hexane/acetone (75/25, *v*/*v*)

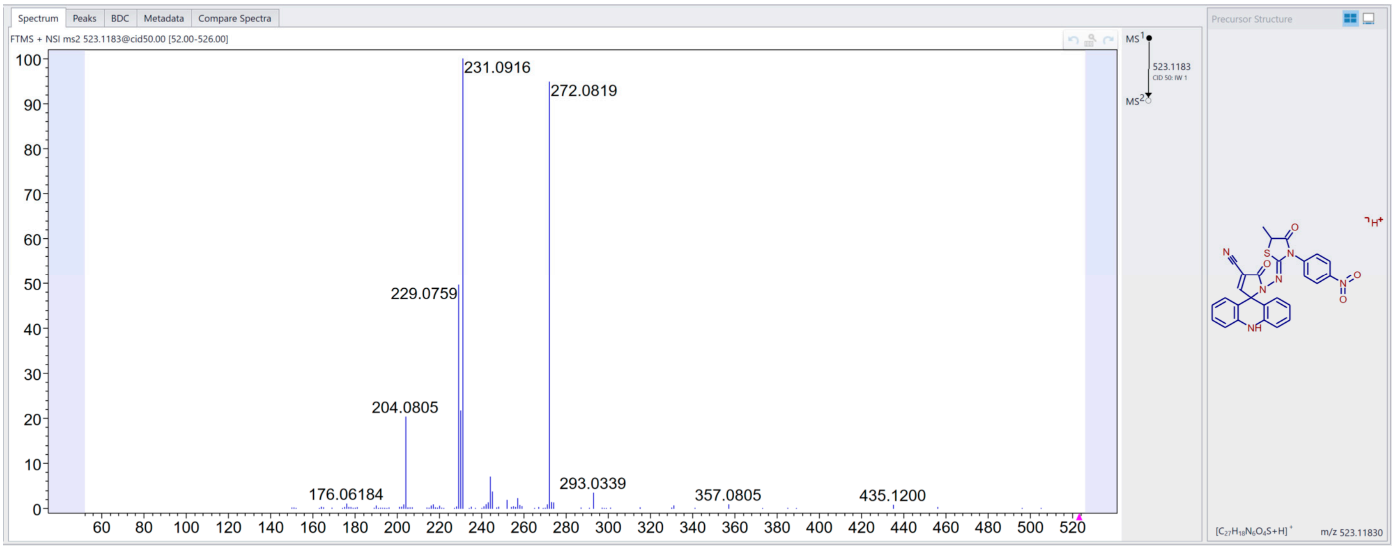

MS2 spectrum of derivative **9e**.

2.21 5'-Oxo-1'-{[(2*Z*)-4-oxo-3-phenyl-5-propyl-1,3-thiazolidin-2-ylidene]amino}-1',5'-dihydro-10*H*-spiro[acridine-9,2'-pyrrole]-4'-carbonitrile (**10a**)

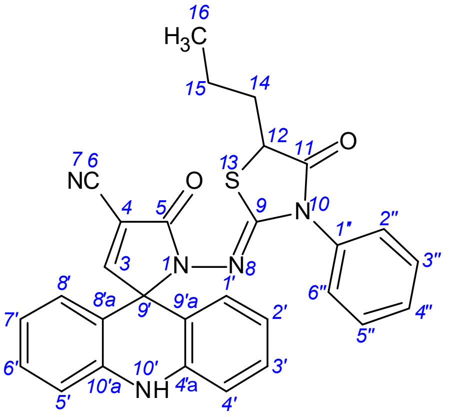

10a

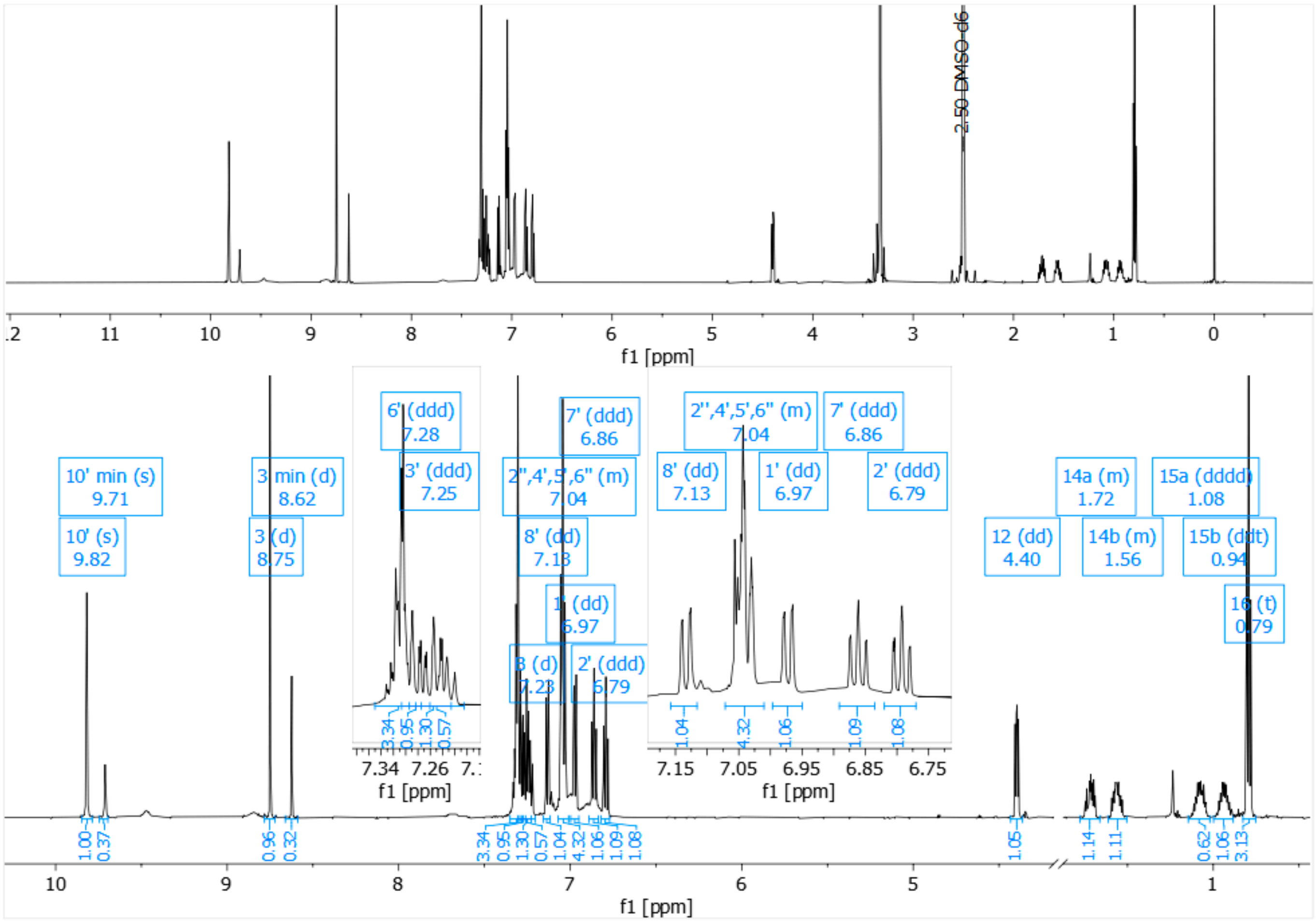

^{1}H NMR (600 MHz, DMSO-d$_6$) spectrum of derivative **10a**.

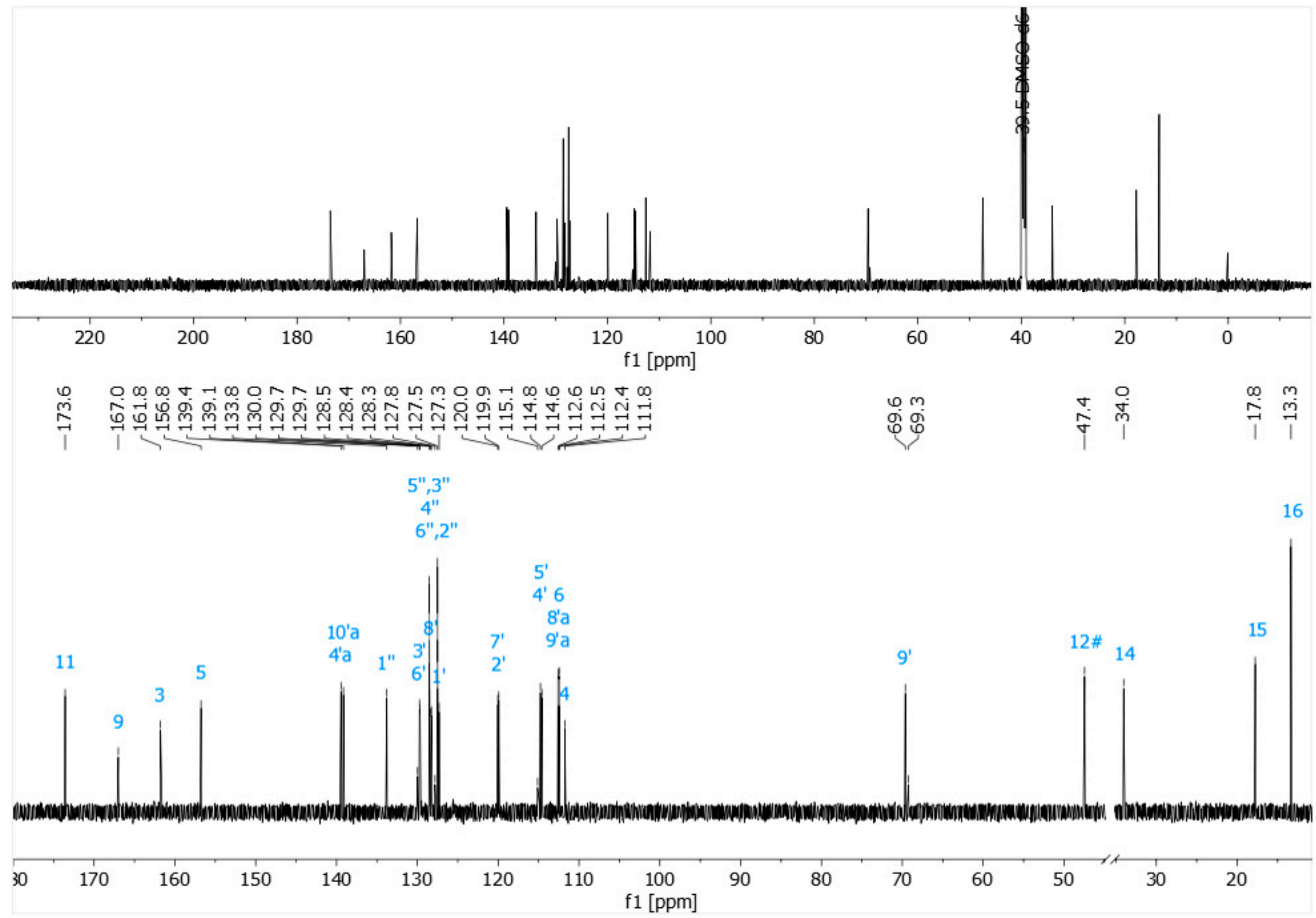

^{13}C NMR (150 MHz, DMSO-d_6) spectrum of derivative **10a**.

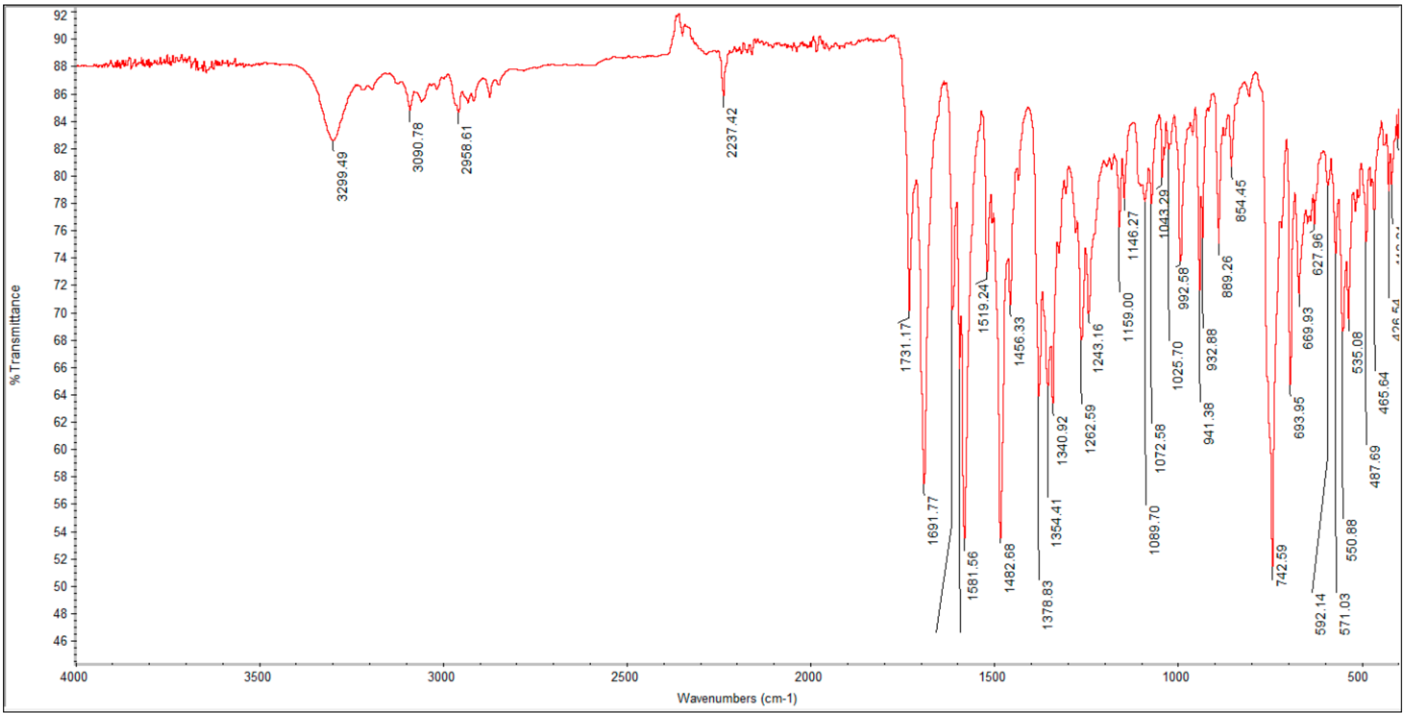

FTIR spectrum of derivative **10a**.

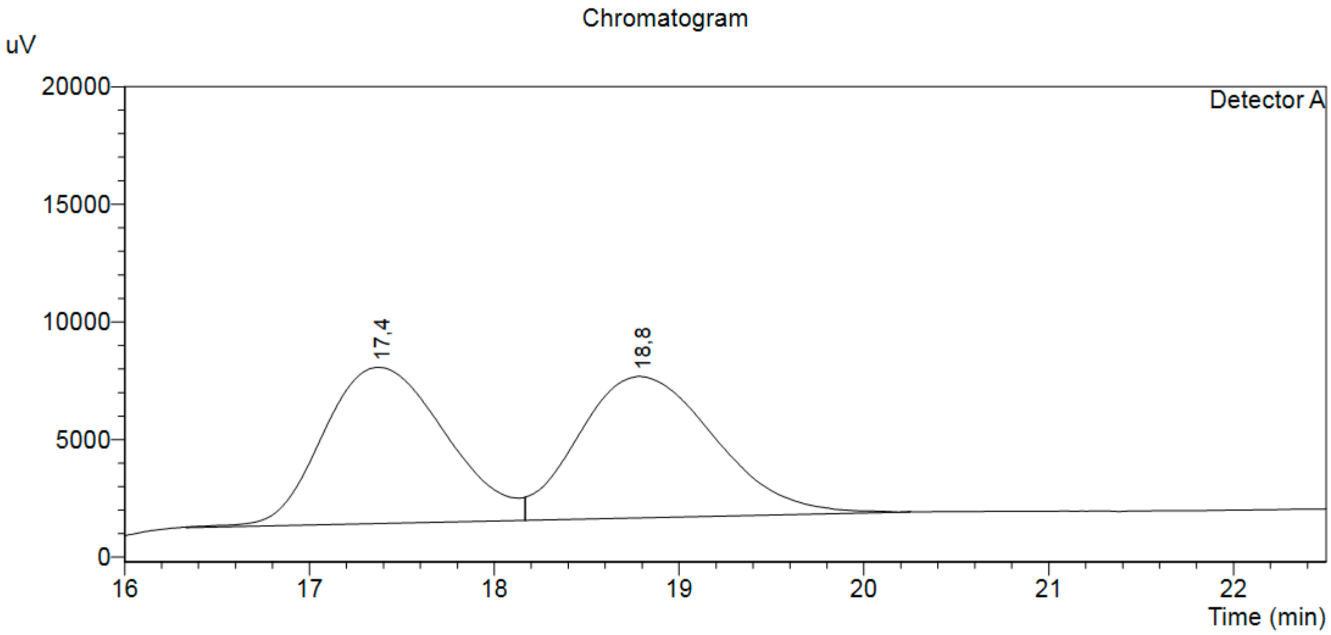

<Peak Table>

Detector A

Peak#	Ret. Time	Area	Height	Conc.	Unit	Area%
1	17,376	306155	6615	49,745		49,745
2	18,788	309290	5991	50,255		50,255
Total		615444	12606			100,000

Chiral HPLC chromatogram of compound **10a**. Chiralpak IA column; flow rate of 0.5 mL/min; temperature 25 °C; *n*-hexane/acetone (75/25, *v*/*v*)

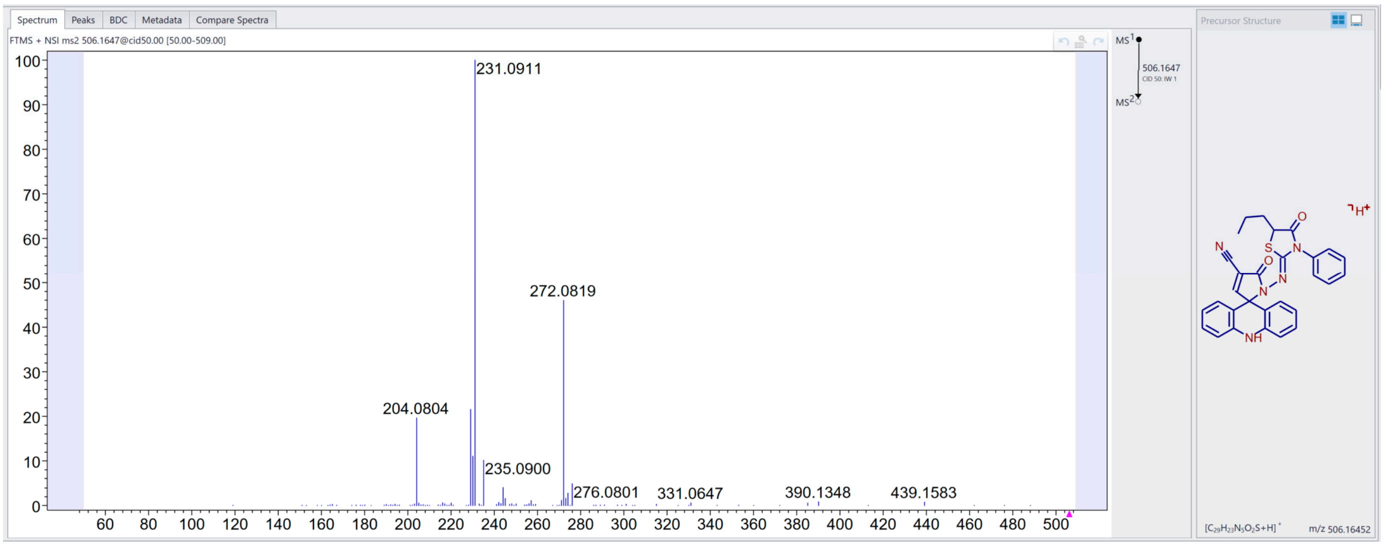

MS2 spectrum of derivative **10a**.

2.22 1'-{[(2*Z*)-3-(4-Methoxyphenyl)-4-oxo-5-propyl-1,3-thiazolidin-2-ylidene]amino}-5'-oxo-1',5'-dihydro-10*H*-spiro[acridine-9,2'-pyrrole]-4'-carbonitrile (10b)

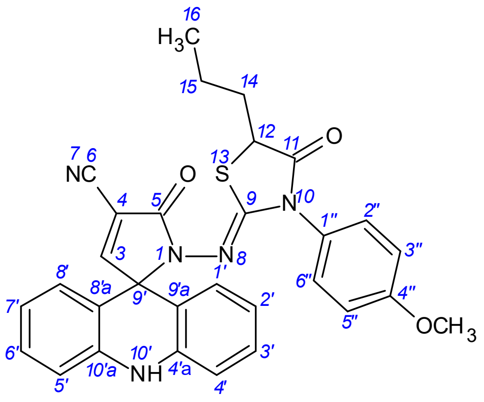

10b

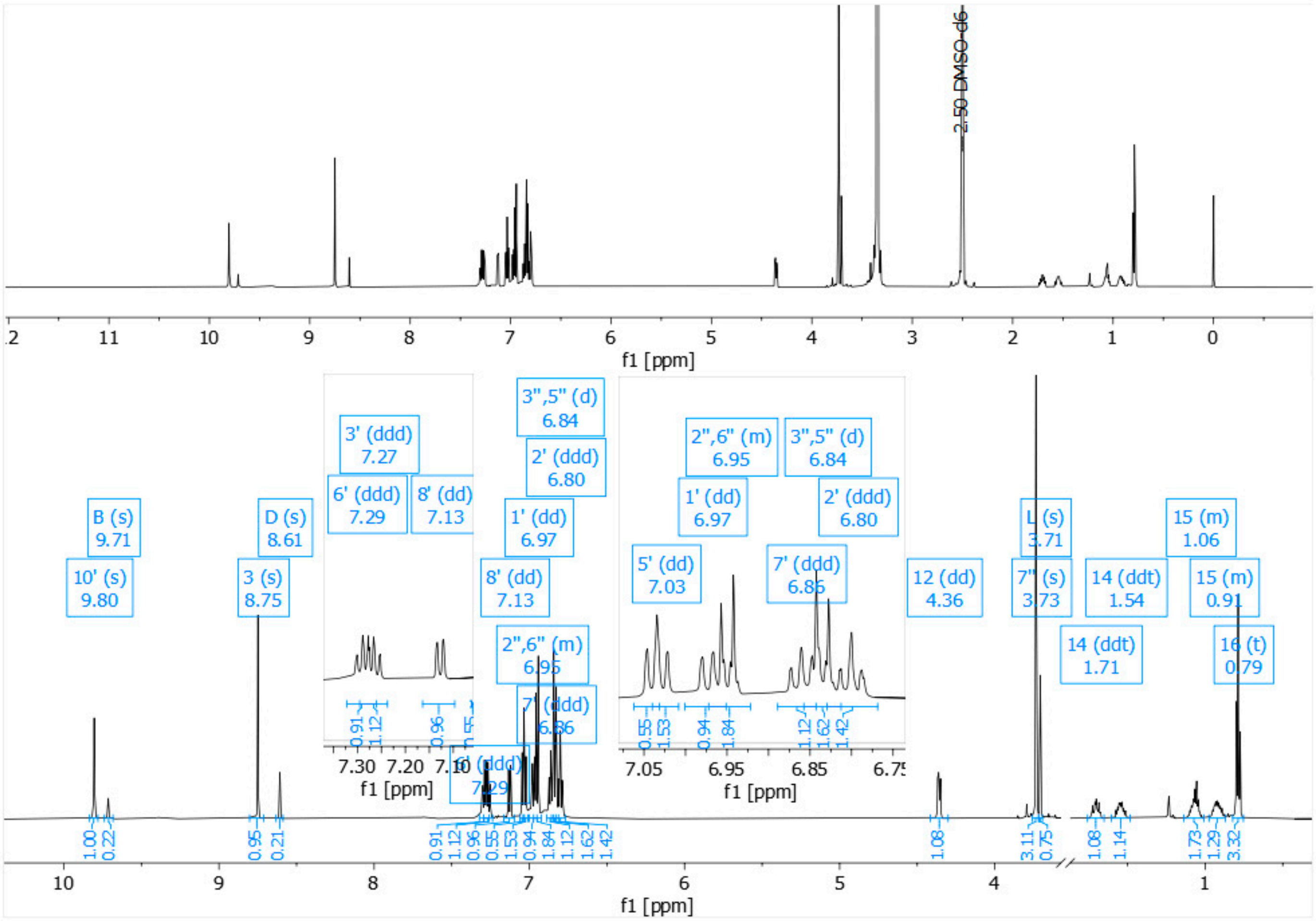

^{1}H NMR (600 MHz, DMSO-d$_6$) spectrum of derivative **10b**.

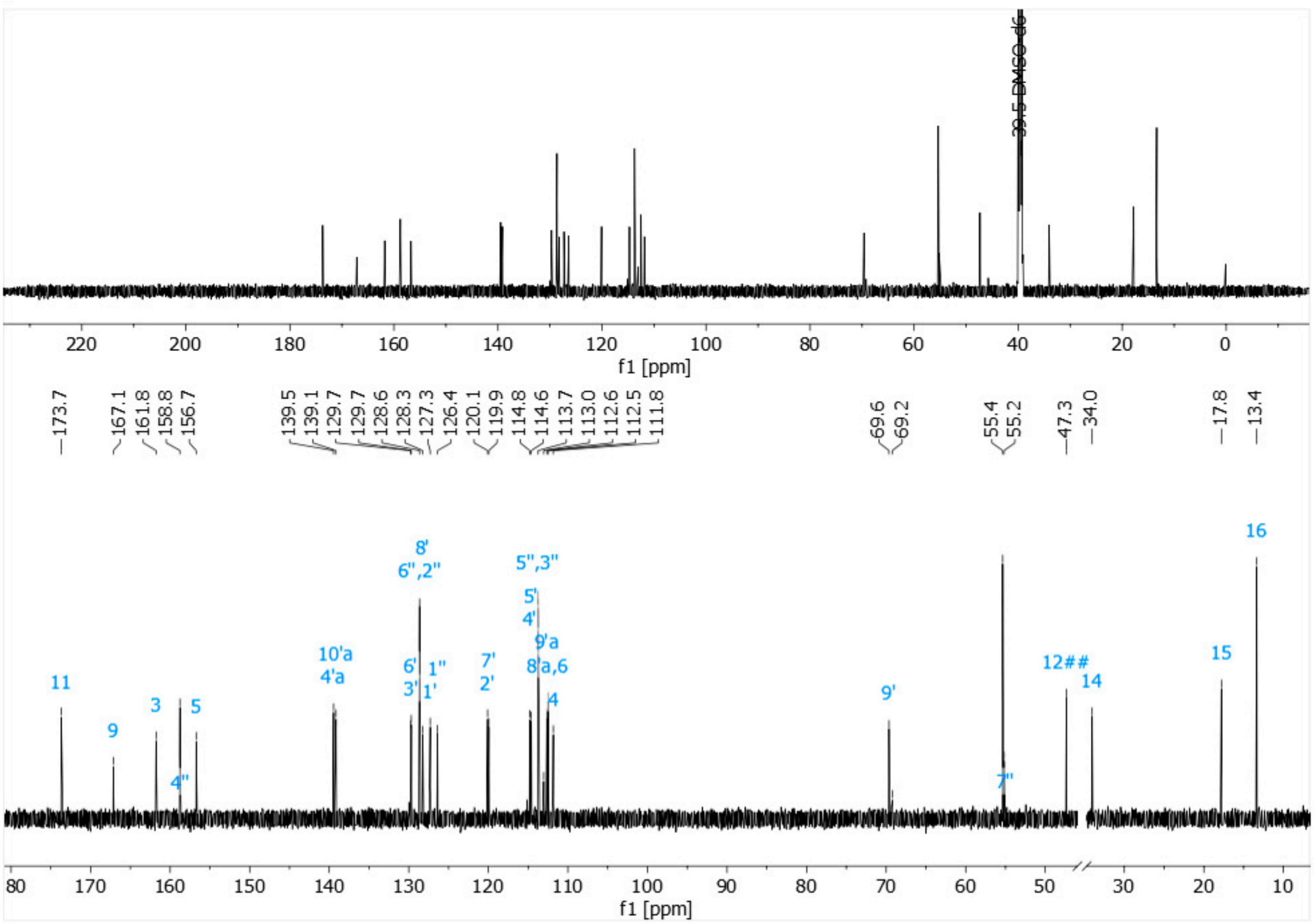

^{13}C NMR (150 MHz, DMSO-d$_6$) spectrum of derivative **10b**.

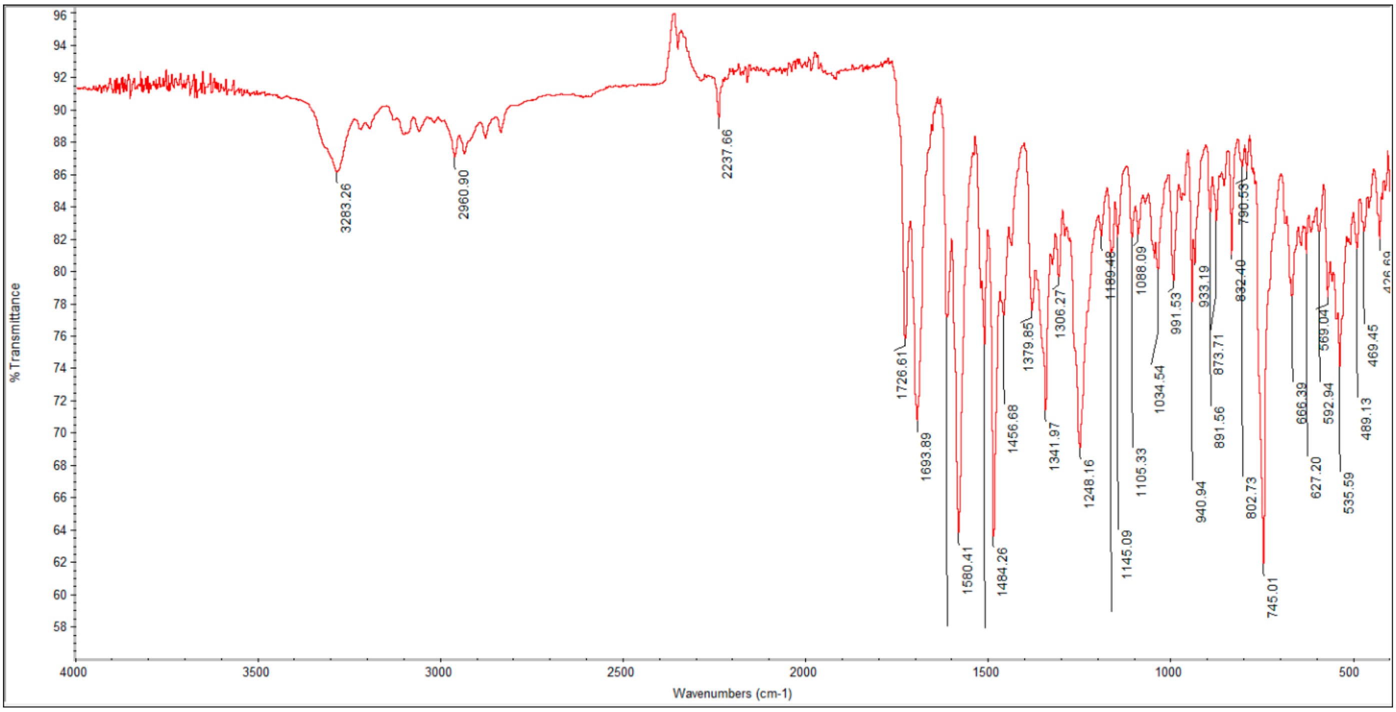

FTIR spectrum of derivative **10b**.

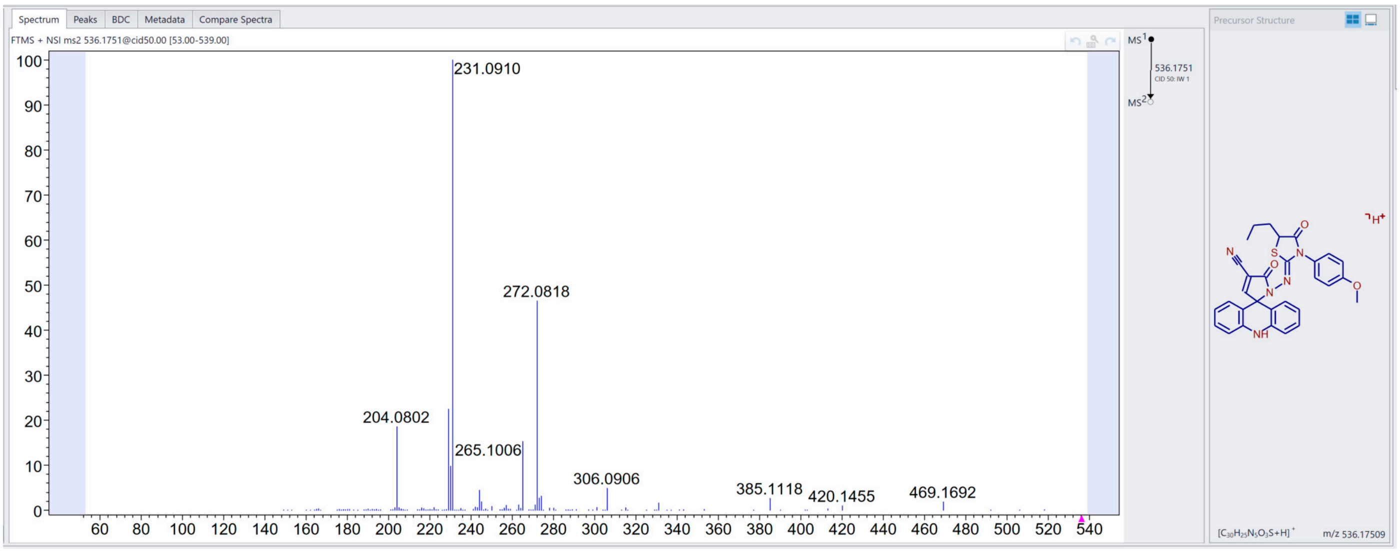

MS2 spectrum of derivative **10b**.

2.23 1'-{[(2Z)-3-(3-Methoxyphenyl)-4-oxo-5-propyl-1,3-thiazolidin-2-ylidene]amino}-5'-oxo-1',5'-dihydro-10H-spiro[acridine-9,2'-pyrrole]-4'-carbonitrile (**10c**)

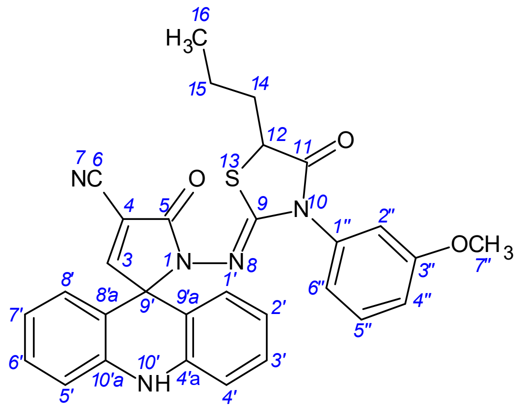

10c

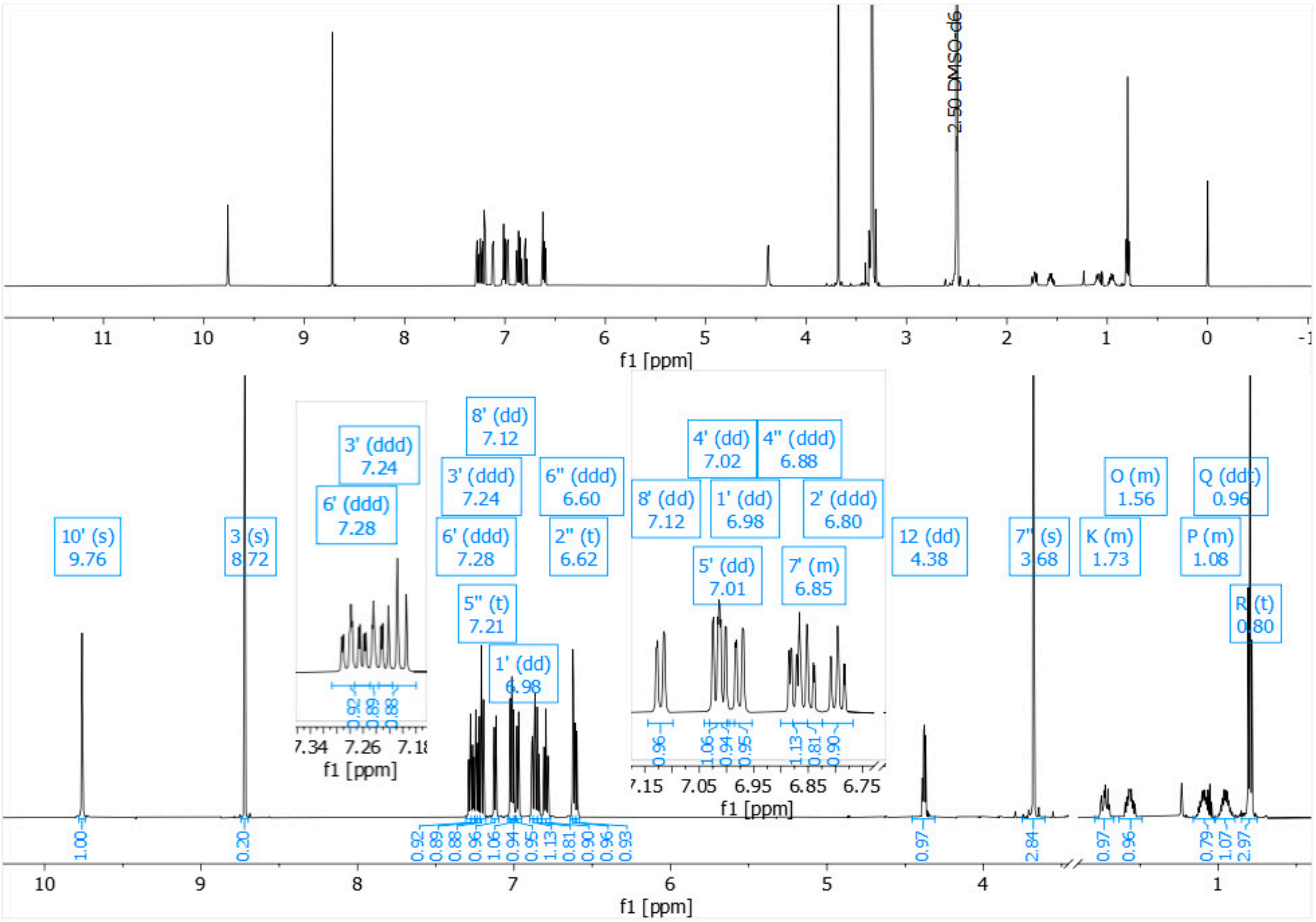

^{1}H NMR (600 MHz, DMSO-d_6) spectrum of derivative **10c**.

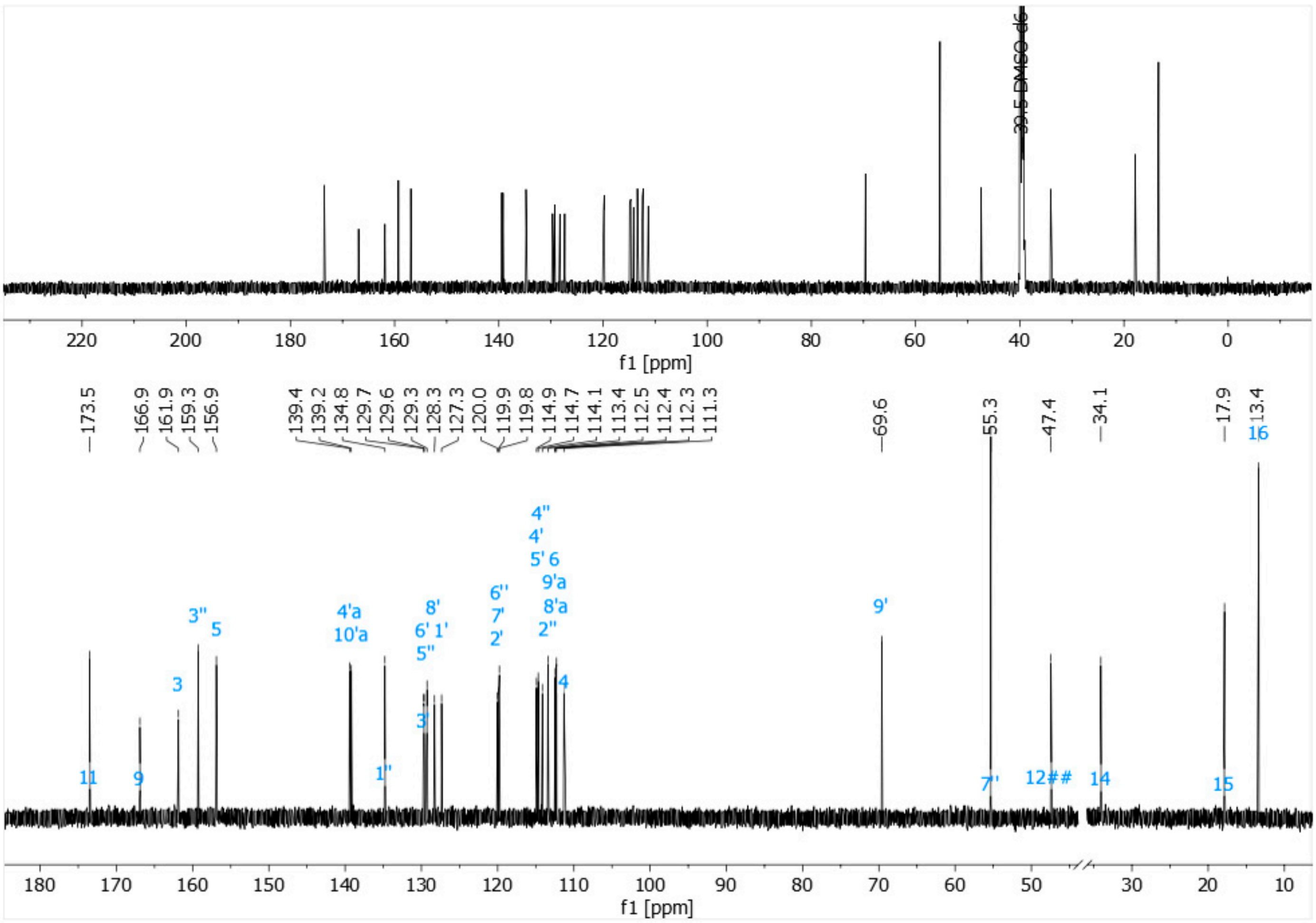

^{13}C NMR (150 MHz, DMSO-d_6) spectrum of derivative **10c**.

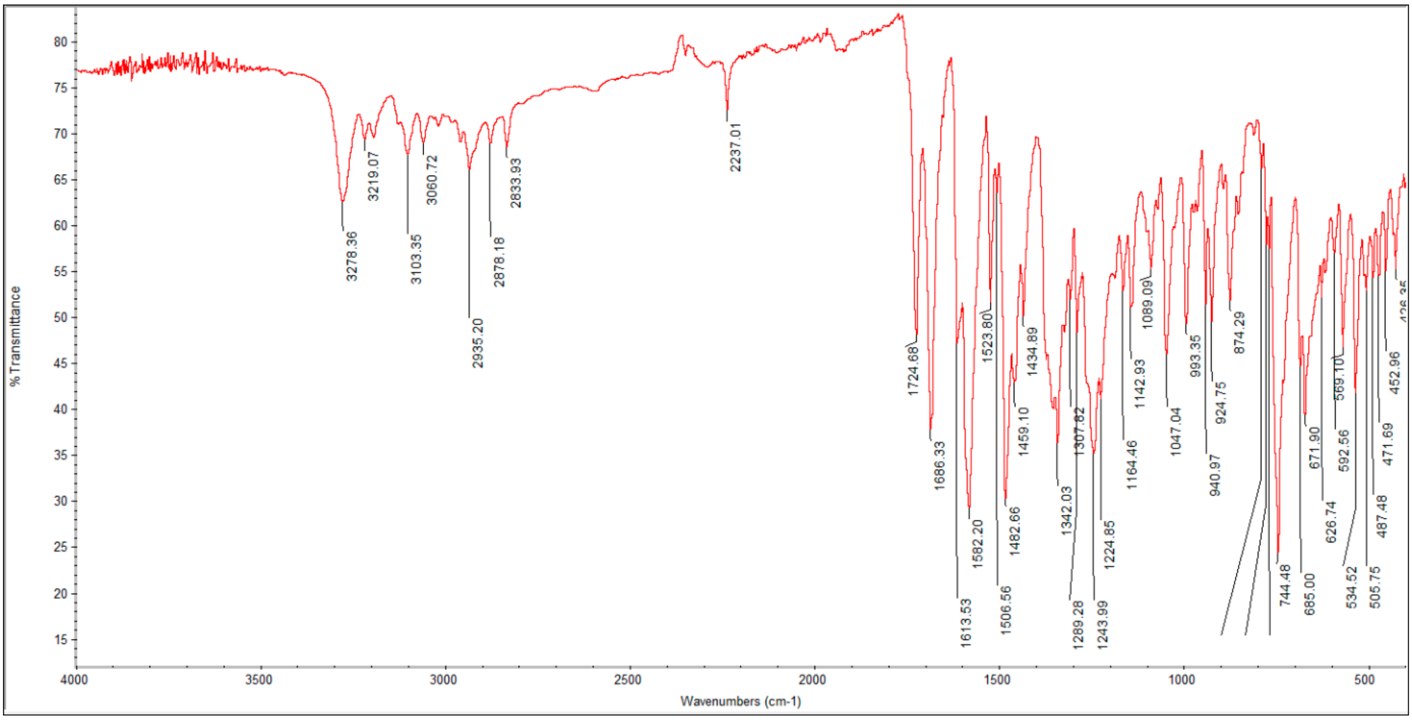

FTIR spectrum of derivative **10c**.

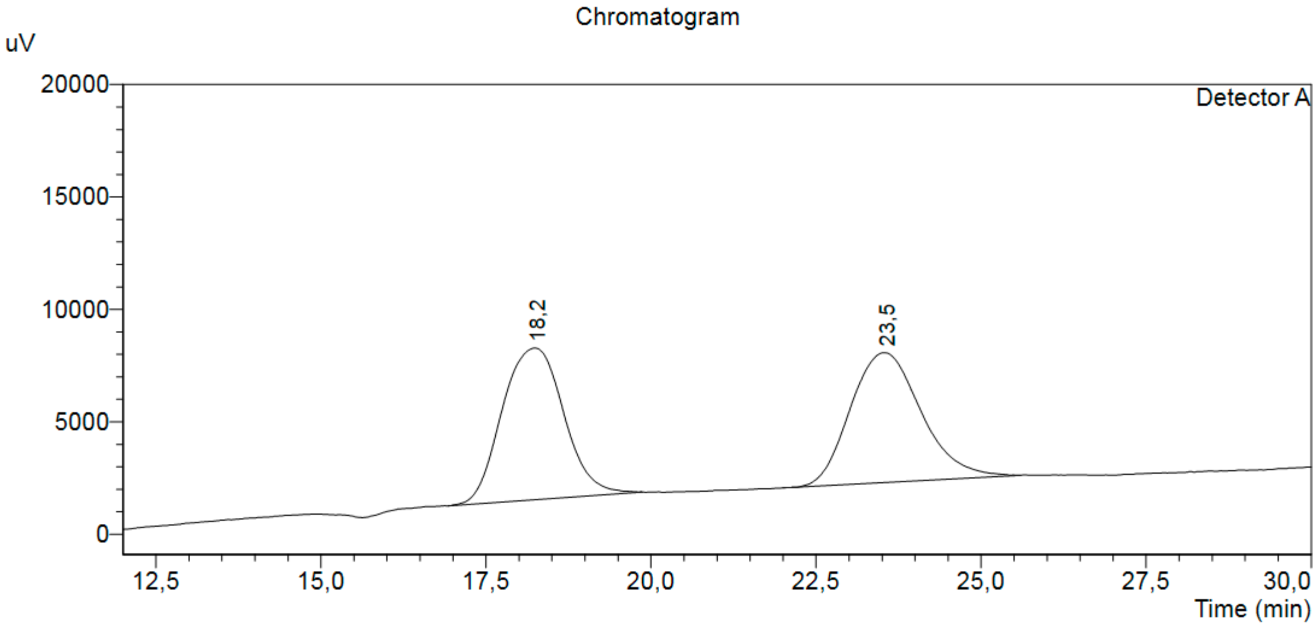

<Peak Table>

Detector A

Peak#	Ret. Time	Area	Height	Conc.	Unit	Area%
1	18,236	438502	6731	50,411		50,411
2	23,533	431355	5768	49,589		49,589
Total		869857	12499			100,000

Chiral HPLC chromatogram of compound **10c**. Chiralpak IA column; flow rate of 0.5 mL/min; temperature 25 °C; *n*-hexane/acetone (75/25, *v*/*v*)

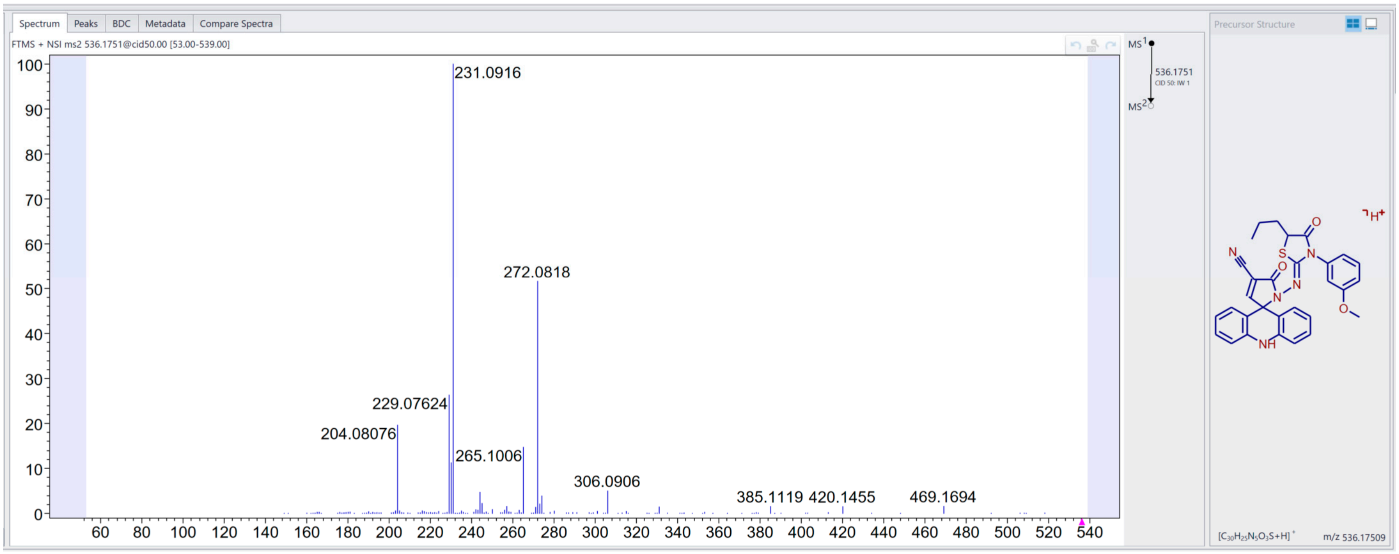

MS2 spectrum of derivative **10c**.

2.24 1'-{[(2*Z*)-3-(4-Fluorophenyl)-4-oxo-5-propyl-1,3-thiazolidin-2-ylidene]amino}-5'-oxo-1',5'-dihydro-10*H*-spiro[acridine-9,2'-pyrrole]-4'-carbonitrile (**10d**)

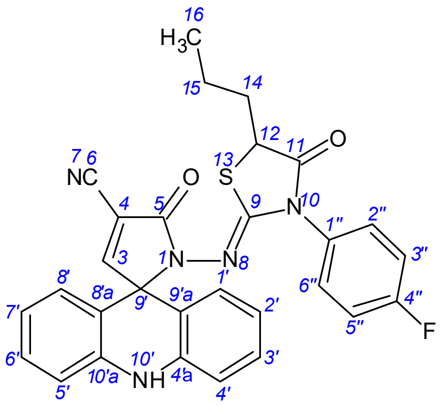

10d

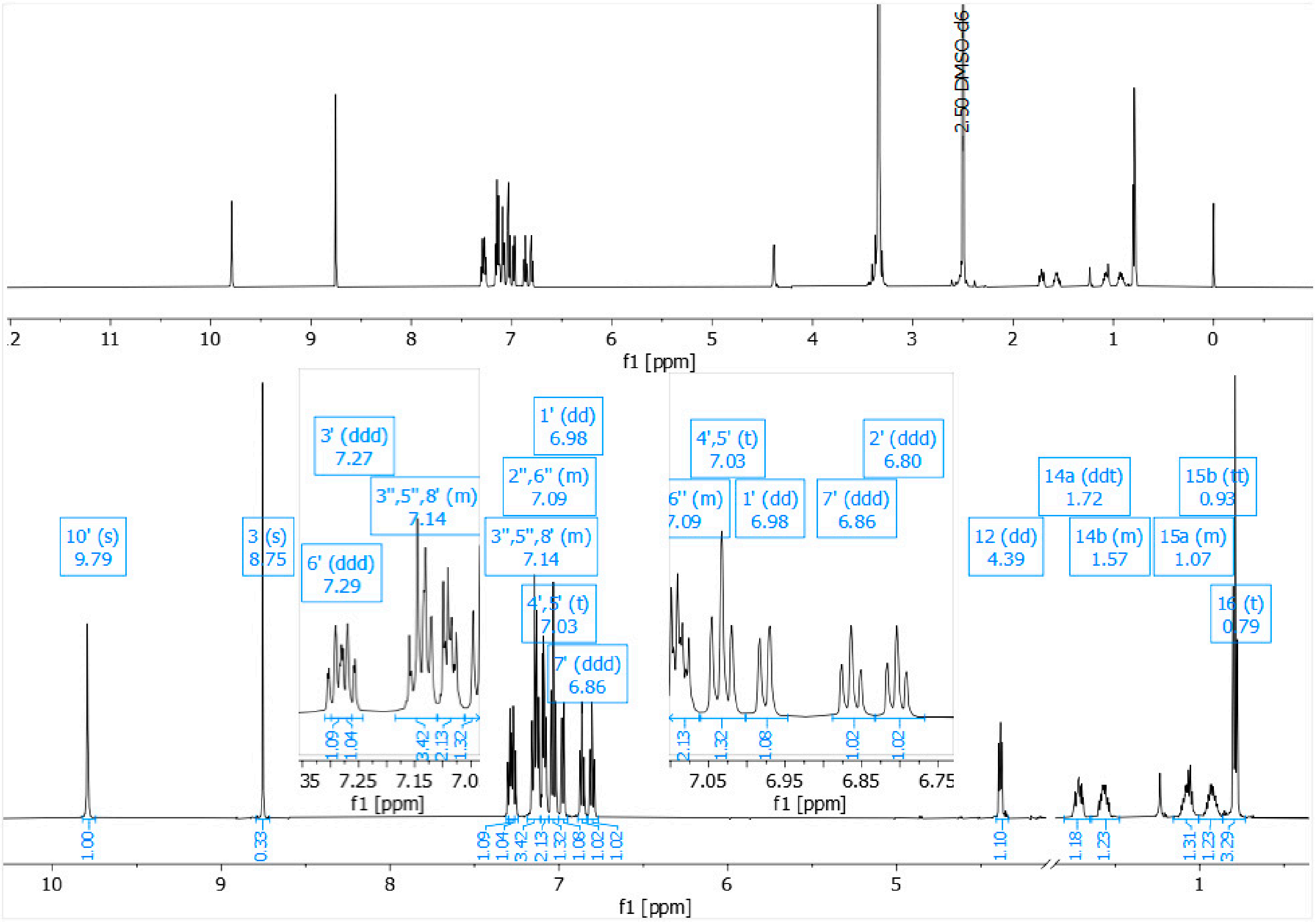

^{1}H NMR (600 MHz, DMSO-d_6) spectrum of derivative **10d**.

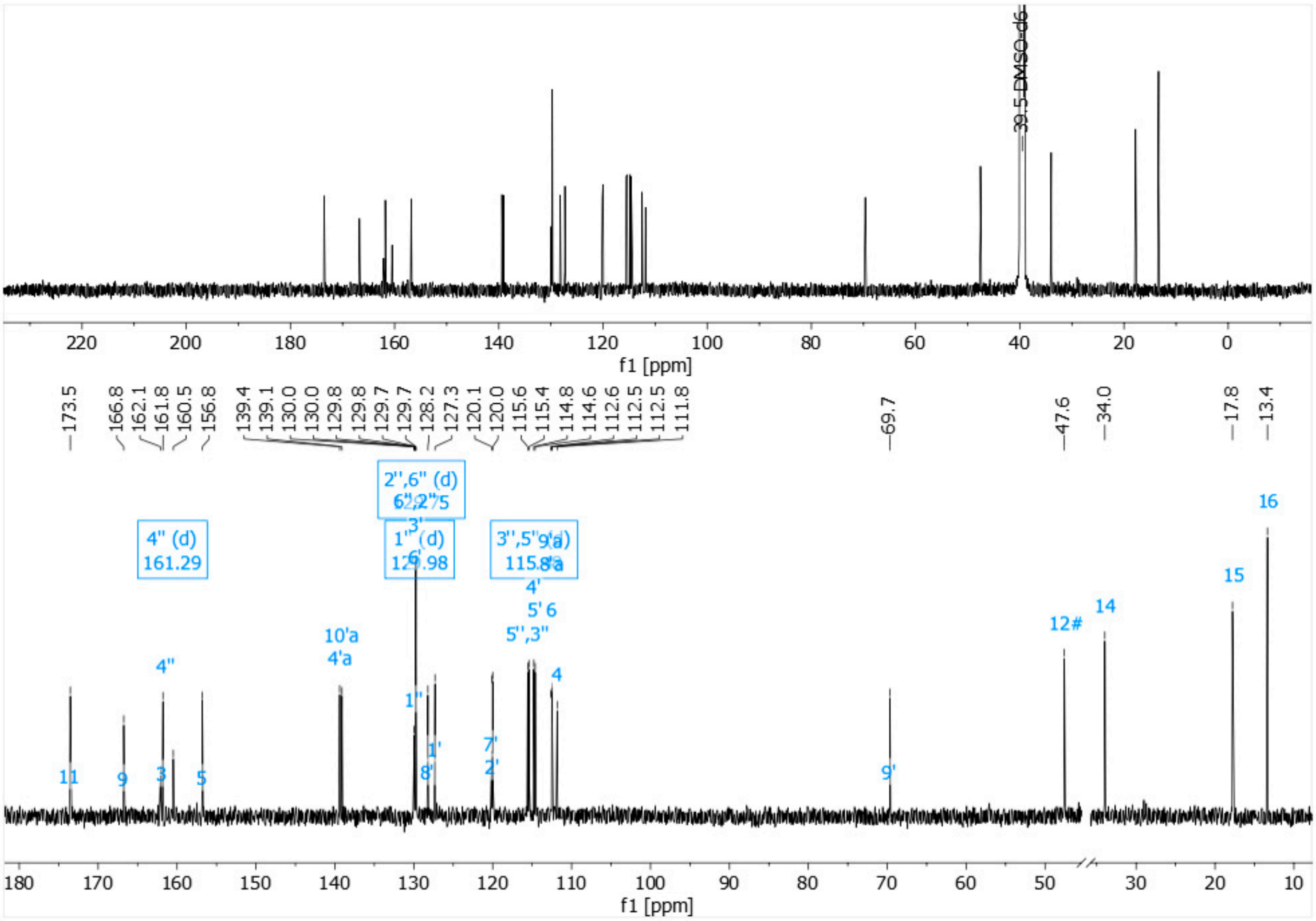

^{13}C NMR (150 MHz, DMSO-d$_6$) spectrum of derivative **10d**.

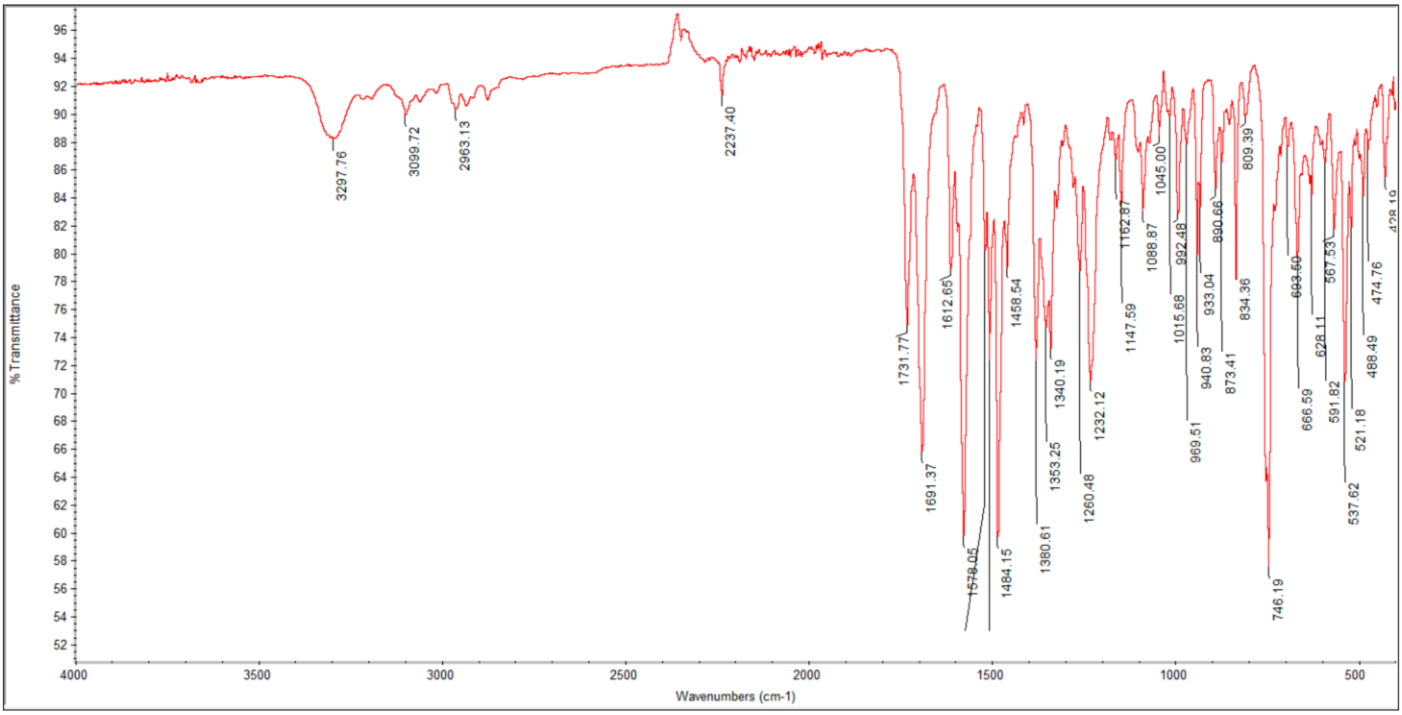

FTIR spectrum of derivative **10d**.

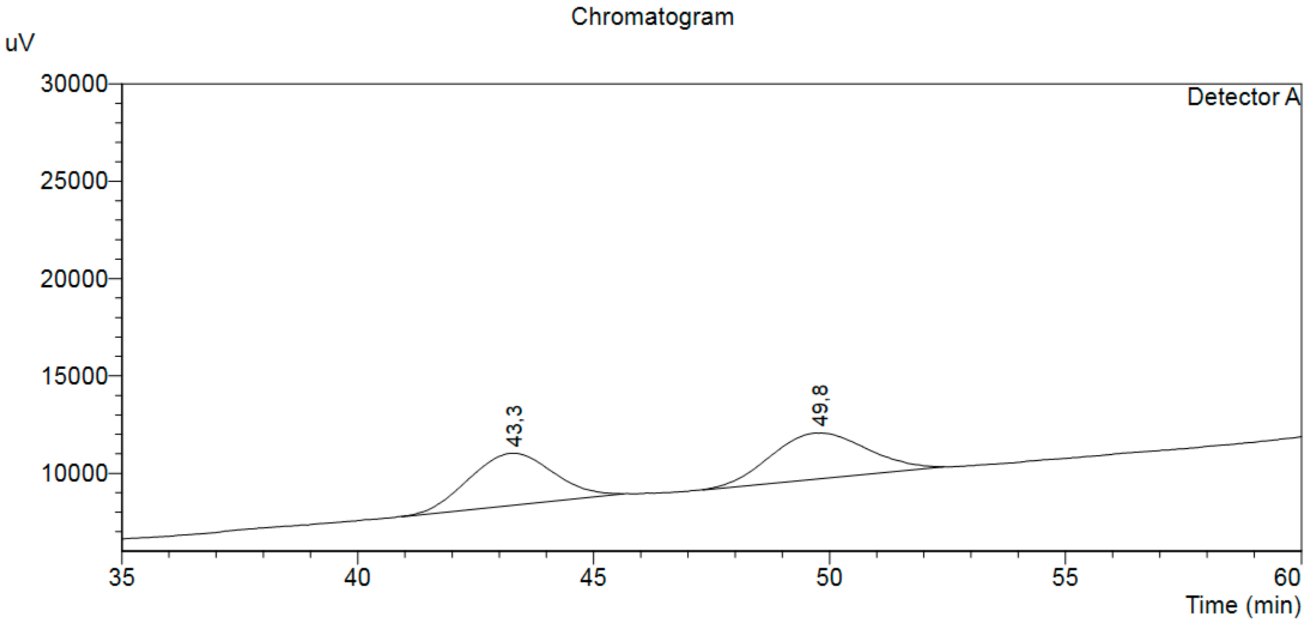

<Peak Table>

Detector A

Peak#	Ret. Time	Area	Height	Conc.	Unit	Area%
1	43,296	331678	2660	49,784		49,784
2	49,773	334556	2349	50,216		50,216
Total		666233	5009			100,000

Chiral HPLC chromatogram of compound **10d**. Chiralpak IA column; flow rate of 0.5 mL/min; temperature 25 °C; *n*-hexane/acetone (85/15, *v*/*v*)

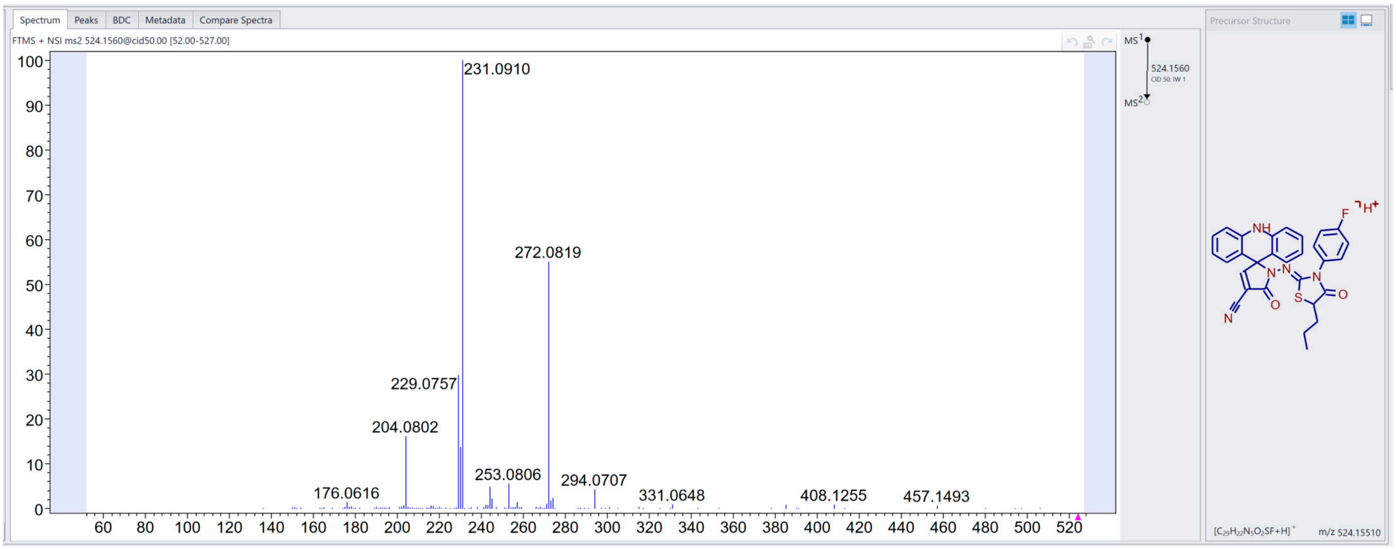

MS2 spectrum of derivative **10d**.

2.25 1'-{[(2*Z*)-3-(4-Nitrophenyl)-4-oxo-5-propyl-1,3-thiazolidin-2-ylidene]amino}-5'-oxo-1',5'-dihydro-10*H*-spiro[acridine-9,2'-pyrrole]-4'-carbonitrile (10e)

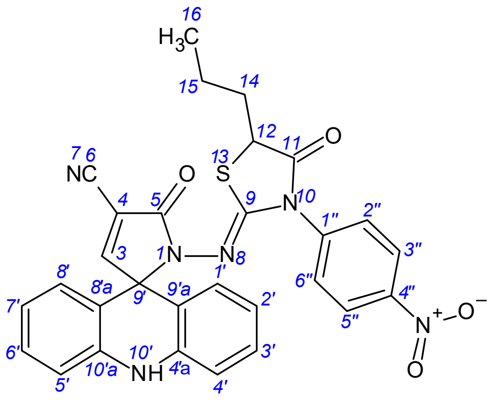

10e

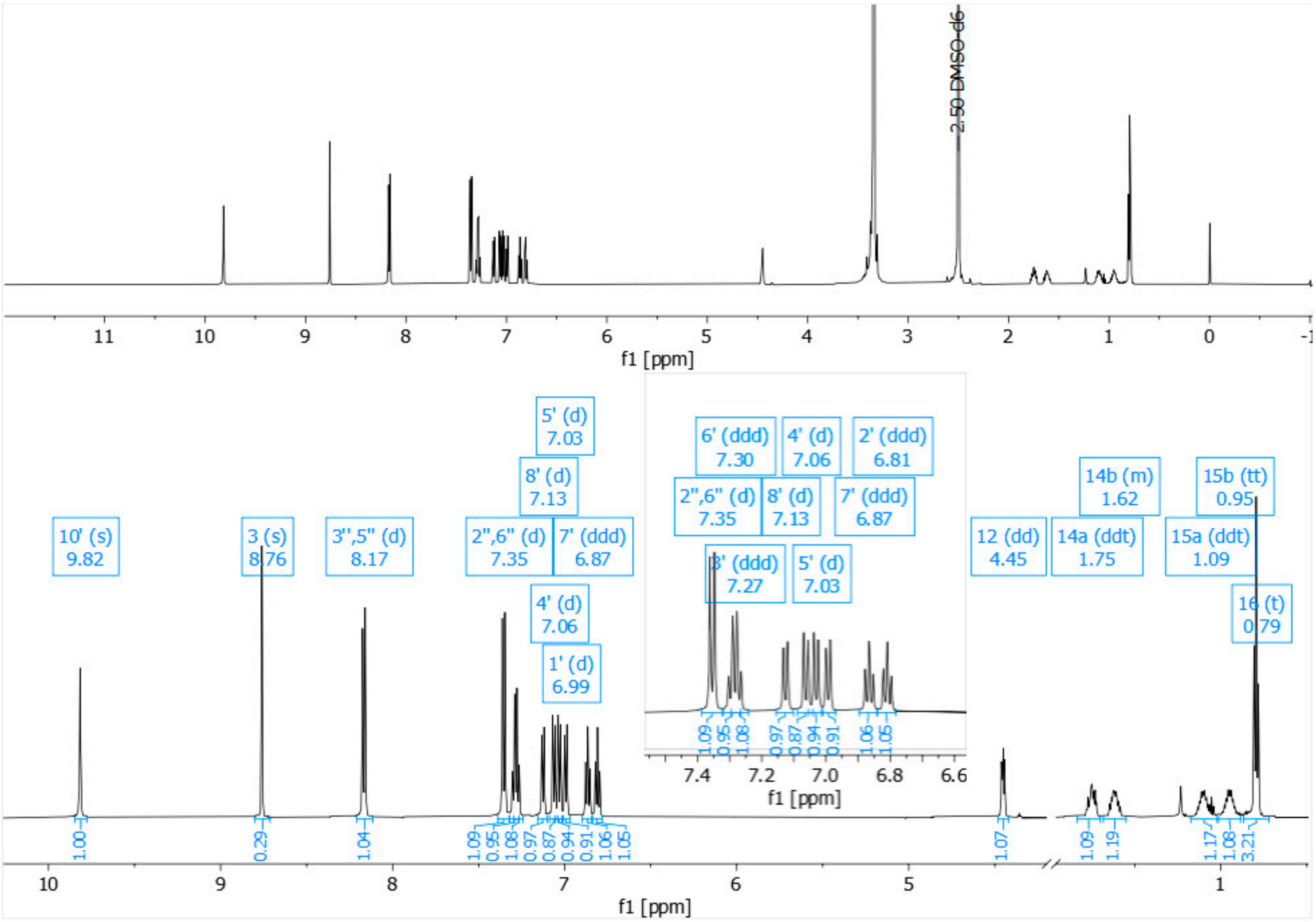

^{1}H NMR (600 MHz, DMSO-d$_6$) spectrum of derivative **10e**.

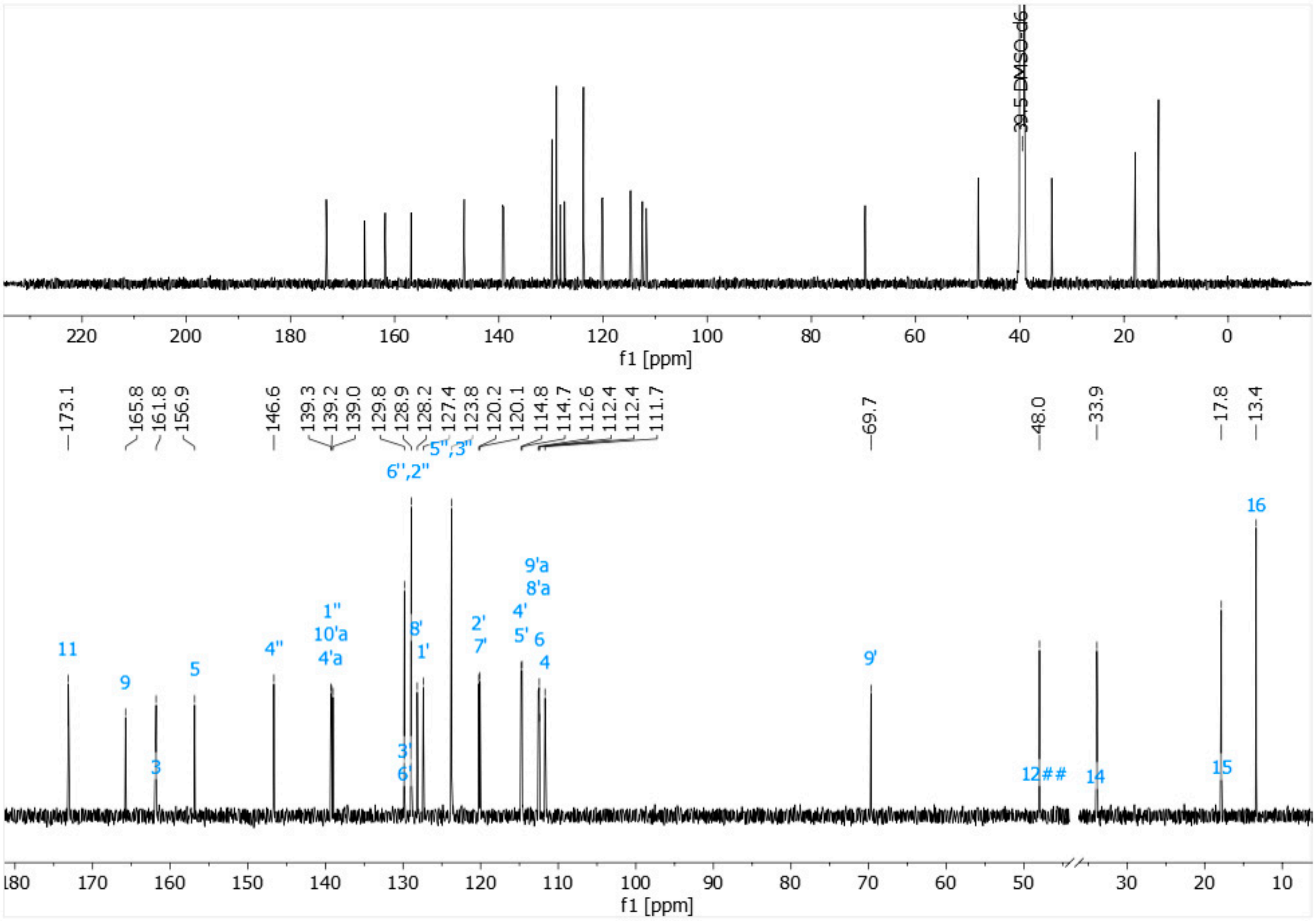

^{13}C NMR (150 MHz, DMSO-d_6) spectrum of derivative **10e**.

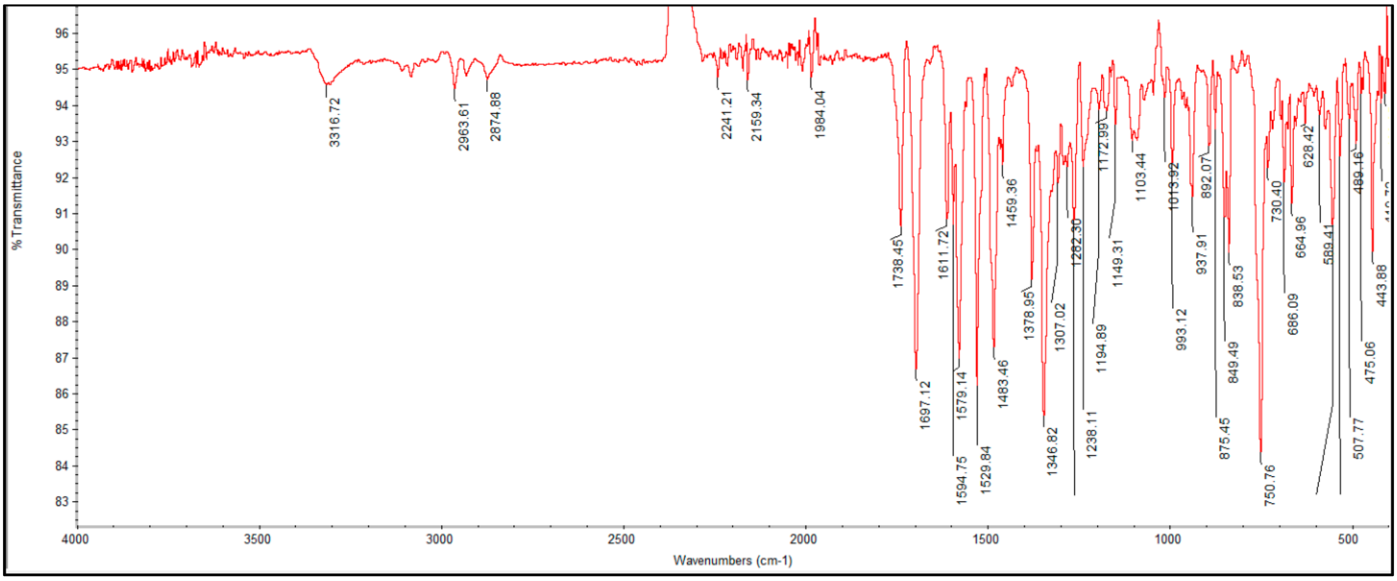

FTIR spectrum of derivative **10e**.

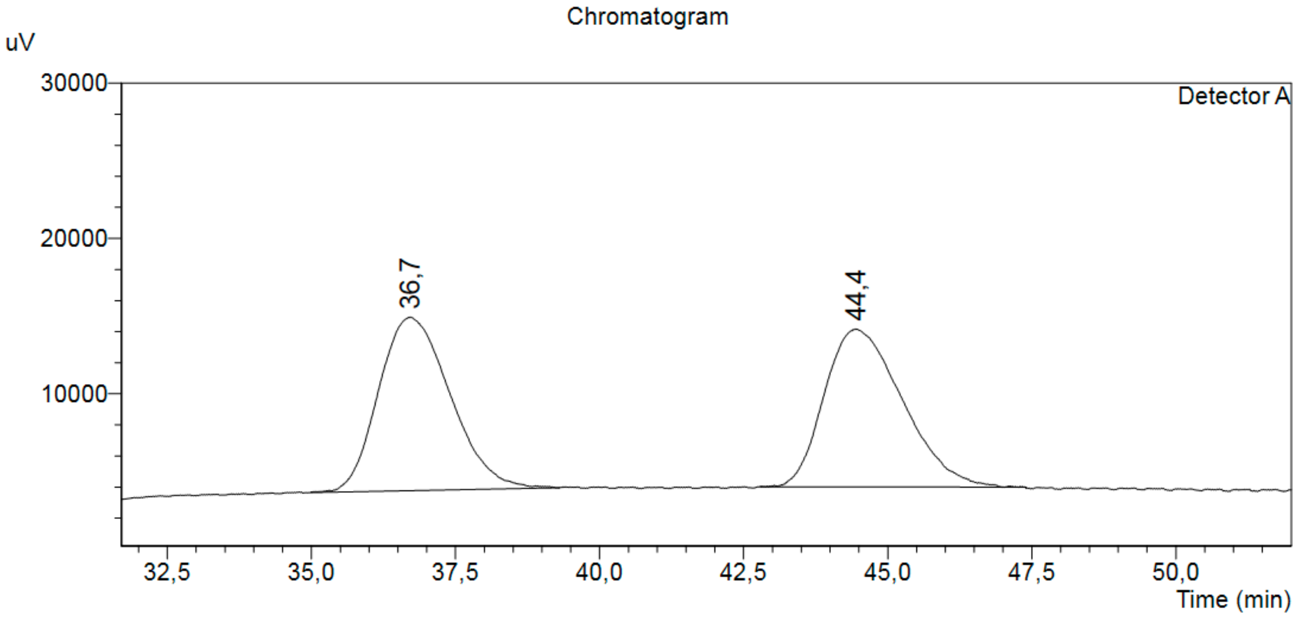

<Peak Table>

Detector A

Peak#	Ret. Time	Area	Height	Conc.	Unit	Area%
1	36,713	965921	11103	50,198		50,198
2	44,447	958295	10137	49,802		49,802
Total		1924216	21239			100,000

Chiral HPLC chromatogram of compound **10e**. Chiralpak IA column; flow rate of 0.5 mL/min; temperature 25 °C; *n*-hexane/acetone (75/25, *v*/*v*)

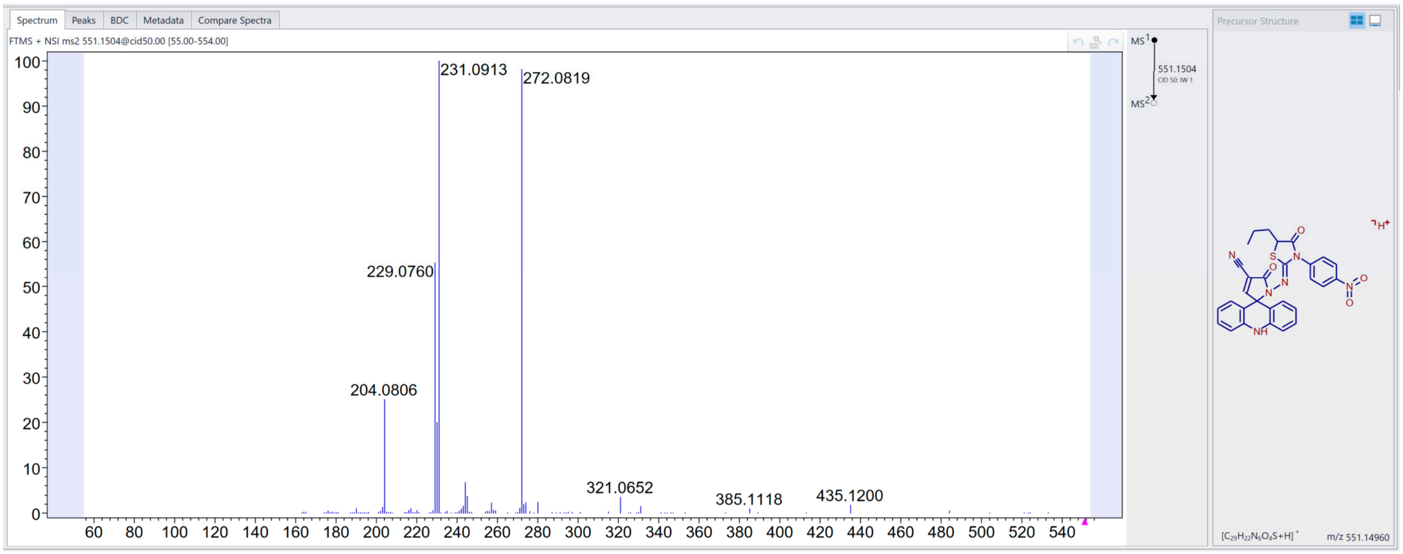

MS2 spectrum of derivative **10e**.
